# A near-chromosome-scale genome assembly of the gemsbok (*Oryx gazella*): an iconic antelope of the Kalahari desert

**DOI:** 10.1093/gigascience/giy162

**Published:** 2019-01-16

**Authors:** Marta Farré, Qiye Li, Yang Zhou, Joana Damas, Leona G Chemnick, Jaebum Kim, Oliver A Ryder, Jian Ma, Guojie Zhang, Denis M Larkin, Harris A Lewin

**Affiliations:** 1 Department of Comparative Biomedical Sciences, Royal Veterinary College, University of London, UK; 2 State Key Laboratory of Genetic Resources and Department of Comparative Biomedical Sciences Evolution, Kunming Institute of Zoology, Chinese Academy of Sciences, Kunming 650223, China; 3 China National Genebank, BGI-Shenzhen, Dapeng New District, Shenzhen 518120, China; 4 Centre for Social Evolution, Department of Biology, Universitetsparken 15, University of Copenhagen, DK-2100 Copenhagen, Denmark; 5 Institute for Conservation Research, San Diego Zoo, Escondido, California, USA; 6 Department of Biomedical Science and Engineering, Konkuk University, Seoul 05029, South Korea; 7 Computational Biology Department, School of Computer Science, Carnegie Mellon University, USA; 8 The UC Davis Genome Center, Department of Evolution and Ecology, College of Biological Sciences, and the Department of Reproduction and Population Health, School of Veterinary Medicine, University of California, Davis, USA

**Keywords:** gemsbok, *Oryx gazella*, assembly, annotation, ruminant, drought

## Abstract

**Background:**

The gemsbok (*Oryx gazella*) is one of the largest antelopes in Africa. Gemsbok are heterothermic and thus highly adapted to live in the desert, changing their feeding behavior when faced with extreme drought and heat. A high-quality genome sequence of this species will assist efforts to elucidate these and other important traits of gemsbok and facilitate research on conservation efforts.

**Findings:**

Using 180 Gbp of Illumina paired-end and mate-pair reads, a 2.9 Gbp assembly with scaffold N50 of 1.48 Mbp was generated using SOAPdenovo. Scaffolds were extended using Chicago library sequencing, which yielded an additional 114.7 Gbp of DNA sequence. The HiRise assembly using SOAPdenovo + Chicago library sequencing produced a scaffold N50 of 47 Mbp and a final genome size of 2.9 Gbp, representing 90.6% of the estimated genome size and including 93.2% of expected genes according to Benchmarking Universal Single-Copy Orthologs analysis. The Reference-Assisted Chromosome Assembly tool was used to generate a final set of 47 predicted chromosome fragments with N50 of 86.25 Mbp and containing 93.8% of expected genes. A total of 23,125 protein-coding genes and 1.14 Gbp of repetitive sequences were annotated using *de novo* and homology-based predictions.

**Conclusions:**

Our results provide the first high-quality, chromosome-scale genome sequence assembly for gemsbok, which will be a valuable resource for studying adaptive evolution of this species and other ruminants.

## Background Information

**Figure 1:**
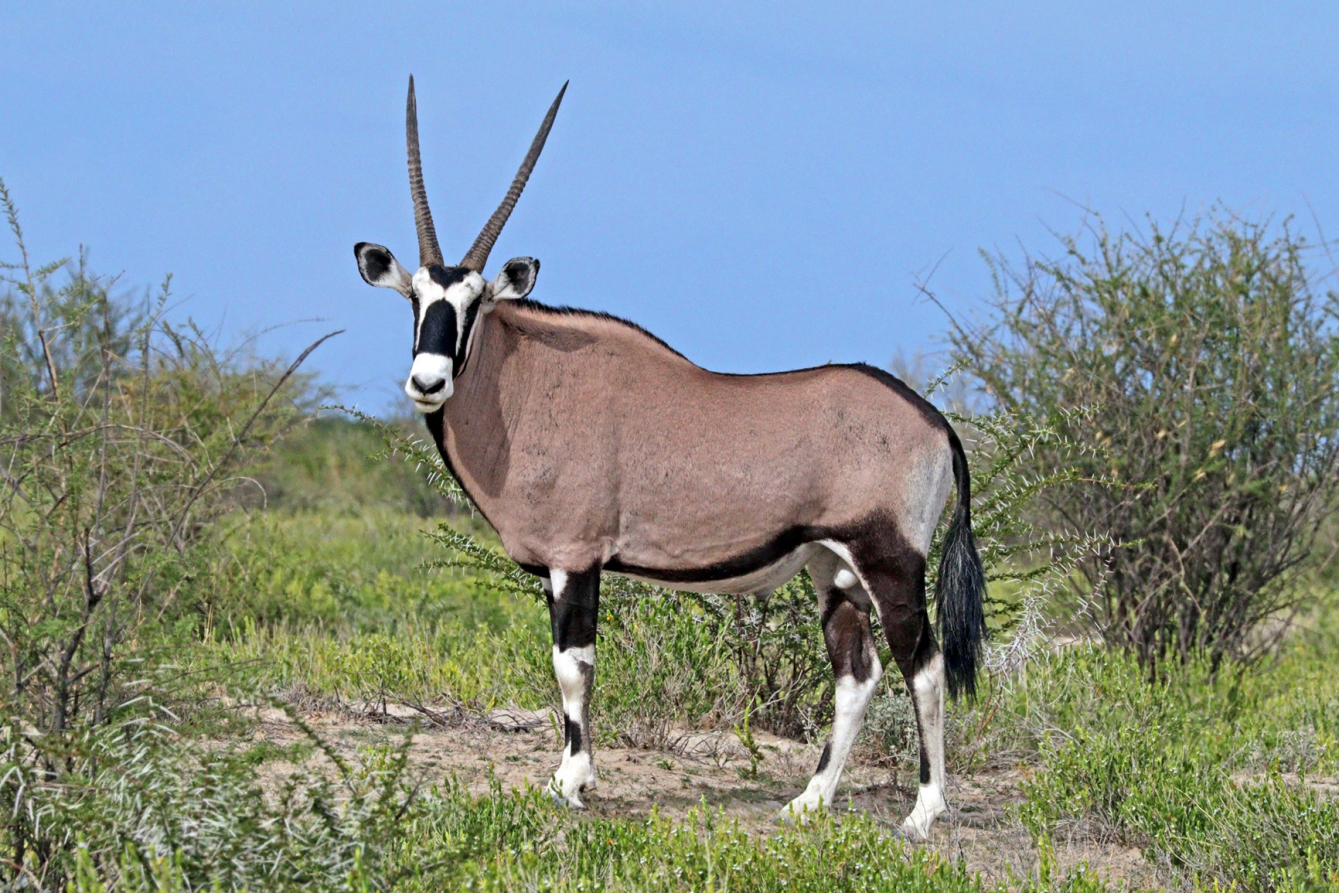
A gemsbok (*Oryx gazella*) male at Etosha National Park (Namibia). Picture from Charles J Sharp QS: P170, Q54800218, Gemsbok (Oryx gazella) male, CC BY-SA 4.0.

The gemsbok (*Oryx gazella*,NCBI:txid9958) is the largest antelope in the genus *Oryx* and a member of the Hippotraginae tribe of ruminants [1] (Fig. 1). The gemsbok's biogeographical distribution includes Botswana and Namibia, traditionally inhabiting the Kalahari and Karoo deserts in southern Africa [2]. The climate of these regions is highly seasonal, with cool winters (10°C–15°C) and hot summers (43°C–46°C) when most of the annual rainfall occurs (90–100 mm). High evaporation rates and low precipitation result in a semi-arid climate in both deserts [3]. Living in such extreme environments, gemsbok have evolved to be highly adapted to drought and extreme heat by minimizing water demand and loss. All of the species in the *Oryx* genus are heterotherms, i.e., they can increase their body temperature from ∼36°C to ∼45°C in order to delay evaporative cooling [4]. *Oryx* species can also change their feeding behavior from grazing to browsing and digging when faced by extreme environmental conditions [5]. Male and female gemsbok are characterized by their low sexual dimorphism, with both sexes having horns and other shared secondary sexual traits [6], making them highly sought after by trophy hunters.

The gemsbok karyotype has 2n = 56 chromosomes, with two Robertsonian translocations compared to cattle [7]. Gemsbok populations have high genetic diversity [8], consistent with other African bovids [9, 10]. Here, we report a chromosome-scale gemsbok genome sequence that will be useful for elucidating the unique adaptations that allow gemsbok to live in arid climates. Several of the large scaffolds are chromosome length or near chromosome length, which will facilitate detailed studies of genome evolution in ruminants. The high-quality, chromosome-scale assembly of the gemsbok contributes to the goals of the Genome 10K Project [11] and the Earth BioGenome Project [12].

## Data Description

### Library construction, sequencing, and filtering

Genomic DNA was extracted from a captive born female gemsbok from the San Diego Safari Park (US) using heart muscle collected at necropsy (NCBI BioSample ID SAMN09604855). High-molecular-weight genomic DNA was obtained using the phenol/chloroform protocol as previously described [13]. Isolated genomic DNA was then used to construct four short-insert sequencing libraries (170, 250, 500, and 800 bp) and eight long-insert libraries (2 Kbp x 2, 5 Kbp x 2, 10 Kbp x 2, and 20 Kbp x 2) following standard protocols provided by Illumina (San Diego, CA). Then, sequencing of the short- and long-insert size libraries was performed using the Illumina Hiseq 2000 platform to generate 301.39 Gbp of raw data (Supplementary Table S1). Reads were trimmed based on low base quality, and reads with more than 5% of uncalled (“N”) bases were removed, providing 179.64 Gbp of filtered read data for genome assembly.

Two Chicago libraries were generated (Dovetail Genomics, Santa Cruz, CA) as previously described [14]. Briefly, high-molecular-weight DNA was assembled into chromatin *in vitro* and then chemically cross-linked before being restriction digested. The overhangs were filled in with a biotinylated nucleotide, and the chromatin was incubated in a proximity-ligation reaction. The cross-links were then reversed, and the DNA was purified from chromatin. After sequencing these libraries on the Illumina Hiseq 4000 platform, we obtained ∼382 million 150 bp read pairs.

### Evaluation of genome size

We used *k*-mer analysis to estimate the size of gemsbok's genome. A *k*-mer refers to an artificial sequence division of K nucleotides iteratively from sequencing reads. A raw sequence read with L bp contains (L-K+1) *k*-mers if the length of each *k*-mer is K bp. The frequency of each *k*-mer can be calculated from the genome sequence reads. Typically, *k*-mer frequencies plotted against the sequence depth gradient follow a Poisson distribution in any given dataset, whereas sequencing errors may lead to a higher representation of low frequencies. The genome size, G, can then be calculated from the formula G = K_num/K_depth, where the K_num is the total number of *k*-mers and K_depth denotes the depth of coverage of the *k*-mer with the highest frequency. In gemsbok, K was 17, K_num was 85,155,457,485, and the K_depth was 26. Therefore, we estimated the genome size of *O. gazella* to be 3.2 Gbp. The filtered reads provided approximately 61.9-fold mean coverage of the genome, while the Chicago library represented 72.7-fold genome coverage.

### Genome assembly

**Figure 2:**
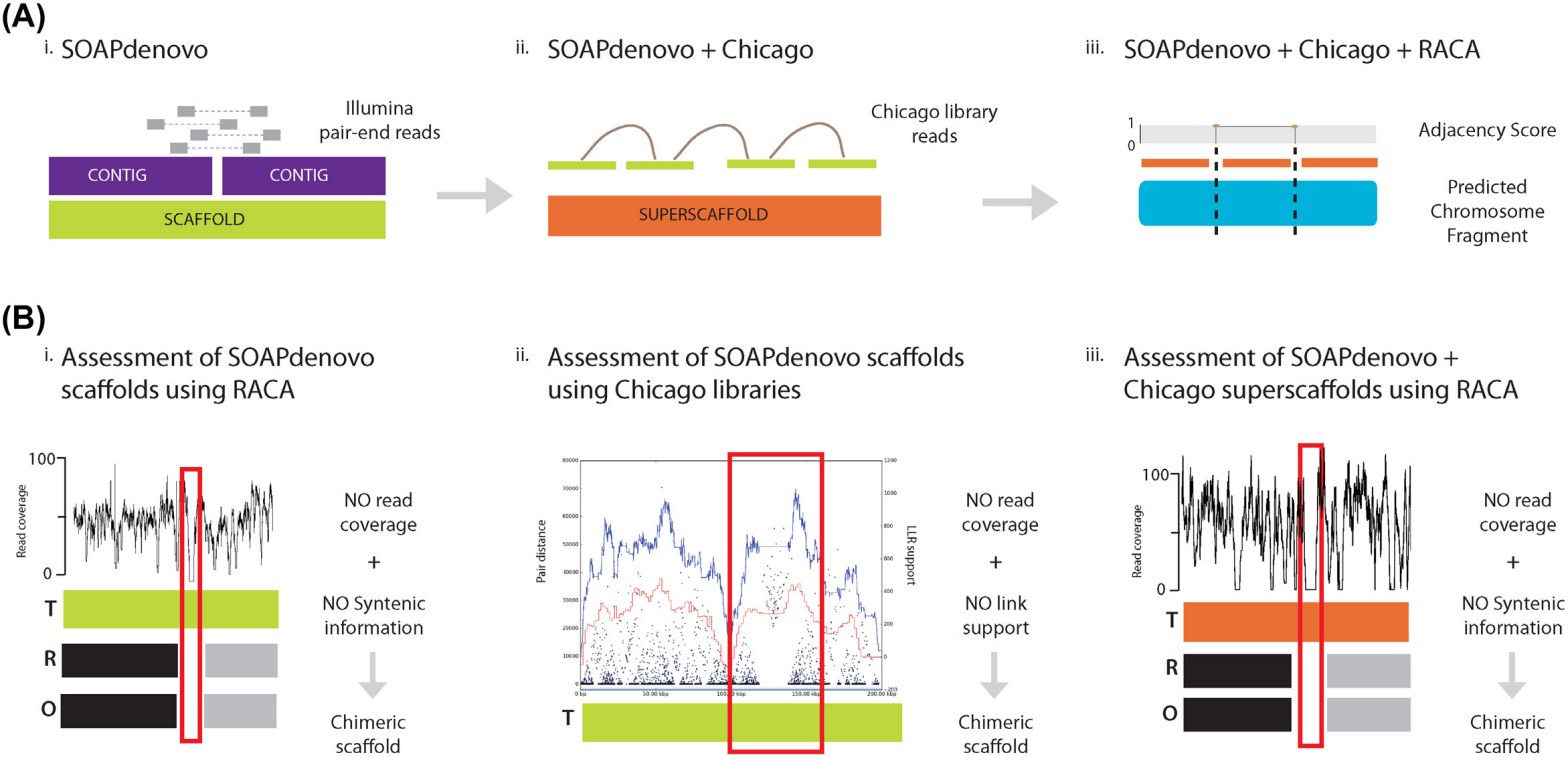
Overview of the approach to generate a chromosome-level gemsbok genome assembly. **(A)** Illumina paired-end and mate-pair reads were assembled into contigs (purple) and then into scaffolds (green) using SOAPdenovo (i). These scaffolds were merged into superscaffolds (orange) using Dovetail Chicago methodology (ii) [11]. Finally, Reference-Assisted Chromosome Assembly tool (RACA) [13] was applied to produce chromosomal fragments (blue) from the superscaffolds (iii). **(B)** To reveal potential chimeric scaffolds, we used the information provided by RACA to identify regions with low read coverage and no syntenic information (demarcated with a red box) in scaffolds (i) or in superscaffolds (iii). The HiRise scaffolder used Chicago libraries sequencing data to pinpoint potentially chimeric regions (shown in the red box) with low read coverage and a substantial reduction of link support (ii). R: reference, T: target and O: outgroup genomes.

We used SOAPdenovo, version 2.04, (SOAP, RRID:SCR_000689), to construct contigs and scaffolds following previously published protocols [15]. The gemsbok genome assembly was 2.90 Gbp long, including 177.88 Mbp (6.13%) of unknown bases. The contig N50 and scaffold N50 sizes were 17.25 Kbp and 1.48 Mbp, respectively (Table 1, Fig. 2A). To assess assembly quality, approximately 98 Gbp (representing genome coverage of 34x) high-quality short-insert size reads were aligned to the assembly using Burrows-Wheeler Aligner (RRID:SCR_010910), with parameters of -t 1 -I [16]. A total of 95.3% reads could be mapped, covering 97.8% of the assembly excluding gaps; 82.1% of these reads were properly paired with an expected insert size associated with the different libraries.

**Table 1:** Assembly statistics of *Oryx gazella* genome

	SOAPdenovo	SOAPdenovo + Chicago	SOAPdenovo + RACA	SOAPdenovo + Chicago + RACA
Input assembly	NA	SOAPdenovo	SOAPdenovo	SOAPdenovo + Chicago
Total length (Mbp)	2,900.52	2,905.93	2,648.75	2,740.44
N50 (Mbp)	1.48	47.03	80.57	86.25
No. scaffolds/PCFs	1,223,903	1,218,509	49	47
No. input scaffolds broken	–	16	12	25

To increase the contiguity of the assembly we used sequence information from the Chicago libraries and the HiRise (version 2.0) scaffolder (Fig. 2a) [14]. A total of 5411 new joins were produced, resulting in a superscaffold N50 of 47.03 Mbp (Table 1).

In parallel, we assembled the gemsbok genome with the Reference-Assisted Chromosome Assembly tool (RACA) [17] using the original SOAPdenovo assembly and raw sequence reads as input (Fig. 2A). Using comparative genomic information and paired-end read mapping to target genome scaffolds, RACA orders and orients scaffolds of a target species into predicted chromosome fragments (PCFs). Only scaffolds longer than 10 Kbp were included in the assembly, accounting for 95% of its length. The cattle (bosTau6) and human (hg19) genomes were used as reference and outgroup, respectively, and all the Illumina paired-end and mate-pair libraries were used in the RACA assembly. Briefly, read libraries were aligned to SOAPdenovo scaffolds using Bowtie2, and syntenic fragments were constructed at 150 Kbp resolution after aligning cattle and gemsbok scaffolds using lastZ and UCSC Kent utilities [18] as previously described [17, 19]. A total of 49 PCFs were reconstructed, of which 21 were homologous to complete cattle chromosomes, and a final PCF N50 of 80.57 Mbp was achieved (Table 1). More than 97% of the scaffold joins introduced in the SOAPdenovo + Chicago assembly were concordant with the RACA assembly, showing a high agreement between both methodologies.

#### Evaluation of SOAPdenovo assembly

To further evaluate the structure of the SOAPdenovo scaffolds, we used the information provided by RACA (Fig. 2B). The RACA evaluation allowed identification of problematic regions in scaffolds with low read physical coverage and not supported by syntenic information from either the reference or the outgroup genomes. As we showed previously [17, 19], 20 to 60% of the flagged problematic scaffolds are chimeric and, therefore, not existent in the genome. In gemsbok, only 12 SOAPdenovo scaffolds were identified as putatively chimeric after running RACA (Table 1).

The HiRise assembler also pinpointed putatively chimeric SOAPdenovo scaffolds using the Chicago libraries sequence information (Fig. 2B). A total of 17 regions in 16 SOAPdenovo scaffolds were identified in this manner. Among the 16 problematic SOAPdenovo scaffolds identified using Chicago library sequence information, 4 were also flagged by RACA, while 4 SOAPdenovo scaffolds were not included in the RACA assembly because they were smaller than 10 Kbp. Seven SOAPdenovo scaffolds were broken in the SOAPdenovo + Chicago assembly, but one of the fragments was below the 150 Kbp resolution chosen to run RACA and therefore not reported in the RACA output. Only two complete disagreements between the SOAPdenovo + Chicago and SOAPdenovo + RACA assemblies were identified.

#### Evaluation of SOAPdenovo + Chicago assembly

To assess the SOAPdenovo + Chicago assembly, RACA was used to identify putative chimeric superscaffolds (Fig. 2B). Because there is no physical or genetic map for gemsbok, we were not able to verify the scaffold adjacencies in PCFs predicted by RACA; therefore, the PCFs were used as a tool to evaluate the SOAPdenovo + Chicago assembly. In this assessment, cattle and human genomes served as the reference and outgroup, respectively, and the SOAPdenovo + Chicago assembly as input. A total of 47 PCFs were reconstructed with N50 of 86.25 Mbp (Table 1), representing 94.5% of the original SOAPdenovo assembly. Nineteen PCFs were orthologous to complete cattle chromosome. Two PCFs corresponding to one complete cattle chromosome were fused to fragments of other chromosomes, and 17 PCFs represented complete independent chromosomes. One PCF represented the complete cattle chromosome 3 in the SOAPdenovo + RACA assembly, while in the SOAPdenovo + Chicago + RACA it was broken into two pieces corresponding to the region with the lowest adjacency score in the SOAPdenovo + RACA assembly. Another PCF was orthologous to cattle chromosome 11, but in the new assembly it was fragmented into two PCFs, one of ∼186 Kbp containing sequence not present in the SOAPdenovo + RACA assembly.

More than 98% of the scaffold joins introduced in the SOAPdenovo + Chicago assembly were consistent with RACA results and are thus likely to be accurate. However, RACA introduced 50 breaks in 25 SOAPdenovo + Chicago scaffolds, suggesting that these scaffolds might be chimeric (Fig. 2B). Of the 50 breaks, 27 comprised joins of SOAPdenovo scaffolds into superscaffolds made using the HiRise assembler. The other 23 breaks were inside single SOAPdenovo scaffolds, with five also being broken in the SOAPdenovo + RACA assembly, while the rest were either not used (4 cases) or below the 150 Kbp resolution of the SOAPdenovo + RACA assembly (14 cases). Although physical or genetic maps for gemsbok are not available to verify the SOAPdenovo + Chicago + RACA assembly, we previously showed that RACA produces highly accurate chromosome assemblies when compared to meiotic linkage [20] or cytogenetic physical maps [19], suggesting that the 47 PCFs of the gemsbok assembly accurately represent scaffold order and orientation on the gemsbok chromosomes. Therefore, using RACA allowed us to identify putatively chimeric scaffolds and superscaffolds, as well as to align components of chimeric scaffolds to their likely location on the gemsbok genome.

**Figure 3:**
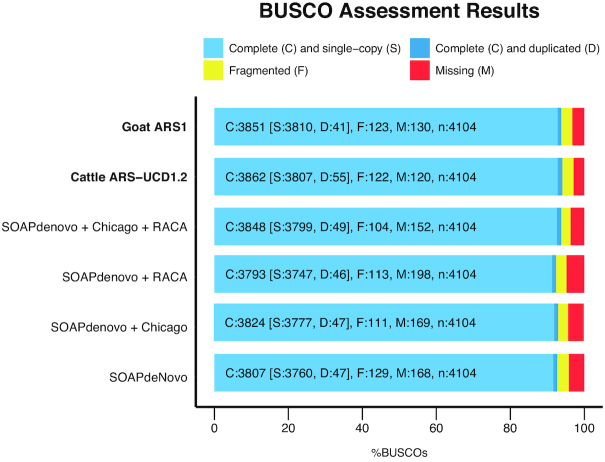
Genome assembly evaluation. The BUSCO dataset of the mammalia_odb9 including 4,104 BUSCOs was used to assess the four assemblies and compared to goat and cattle ARS-UCD1.2.

**Figure 4:**
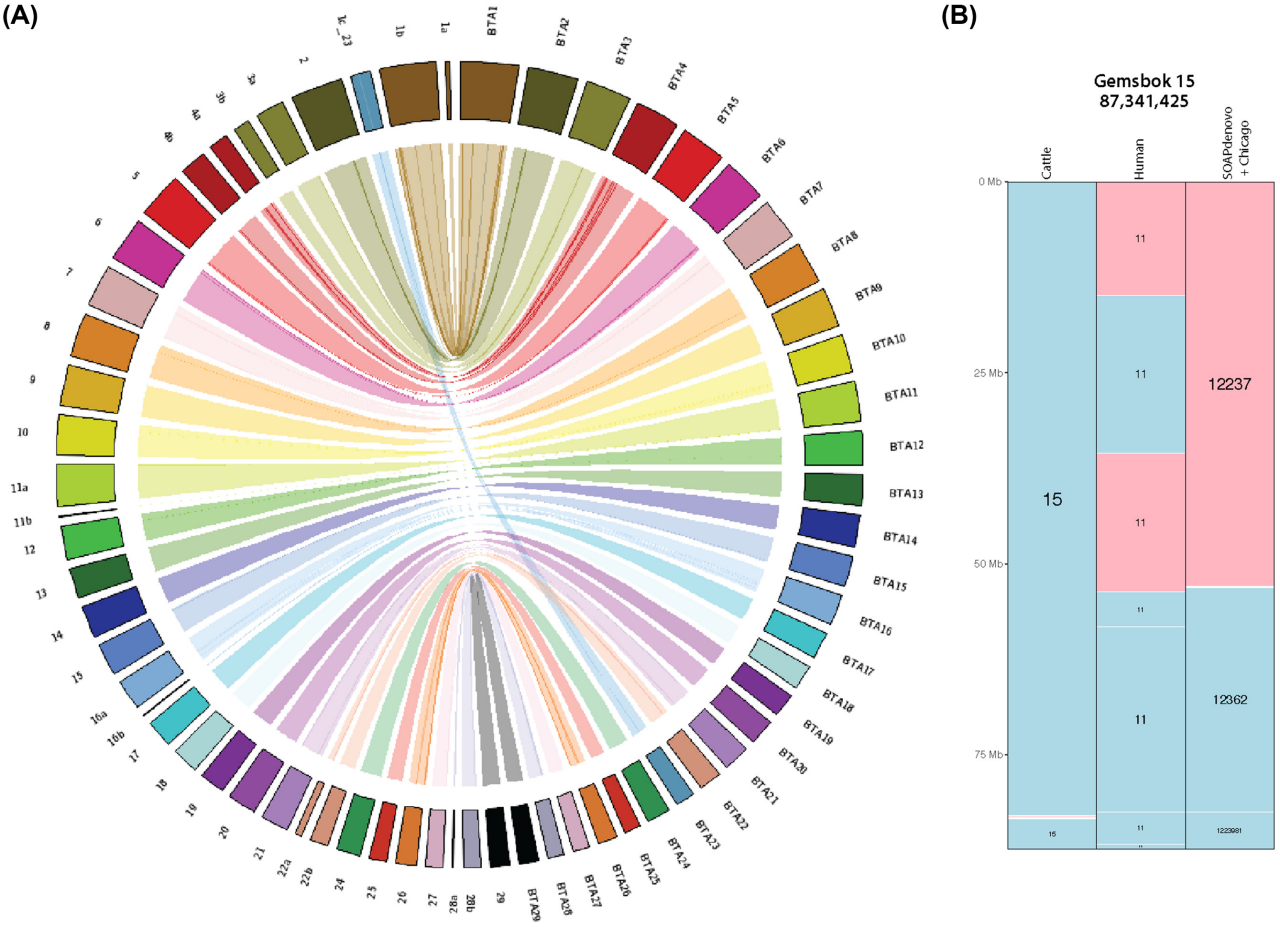
Syntenic relationships between gemsbok and cattle genomes. **(A)** Circos plot showing syntenic relationships between cattle autosomes (labeled as BTA) and gemsbok chromosomal fragments. Chromosomes are colored based on cattle homologies. Ribbons inside the plot show syntenic relationships, while lines inside each ribbon indicate inversions. **(B)** Gemsbok chromosome 15 showing homologous synteny blocks (HSBs) between gemsbok, cattle, and human. SOAPdenovo + Chicago scaffolds are also displayed. The other gemsbok chromosomes can be found in Supplementary Fig. S1.

Genome completeness was assessed using the Benchmarking Universal Single-Copy Orthologs (BUSCO, RRID:SCR_015008) [21]) software, version 3.0. More than 92% of the core mammalian gene set was complete in all assemblies (Fig. 3), with the SOAPdenovo + Chicago + RACA assembly being the most complete, containing 96.3% of the gene set with 93.8% being complete. The percentage of complete genes in this assembly is similar to other recent ruminant assemblies (93.8% and 94.1% in goat ARS1 and cattle ARS-UCD1.2, respectively; Fig. 3), showing that the Gemsbok SOAPdenovo + Chicago + RACA assembly is of similar quality. Finally, we assessed the genome continuity by identifying homologous synteny blocks (HSBs) between gemsbok and cattle chromosomes (Supplementary Fig. S1). Gemsbok (2n = 56) and cattle (2n = 60) karyotypes differ by two Robertsonian translocations [7], but only one of them is present in the gemsbok assembly (Fig. 4). A total of 21 cattle chromosomes aligned to an individual gemsbok fragment, indicating that they represent complete gemsbok chromosomes. Eight cattle chromosomes (BTA1, BTA3, BTA4, BTA11, BTA16, BTA22, BTA28, and BTAX) were syntenic to two or more gemsbok HSBs, suggesting that these HSBs represent chromosomal fragments. The HSBs were physically assigned to chromosomes based on known syntenic relationships to cattle chromosomes [7].

### Genome annotation

To annotate the gemsbok genome, we started by mapping transposable elements (TEs). The TEs were predicted in the genome by homology to RepBase sequences using RepeatProteinMask and RepeatMasker (RepeatMasker, RRID:SCR_012954) [22] with default parameters, then the results were combined to produce a non-redundant final set. About 42.5% of the gemsbok genome is comprised of TEs, with Long Insterspersed Nuclear Elements (LINEs) being the most frequent class (25.71%; Supplementary Table S2).

The rest of the genome assembly was annotated using both homology-based and *de novo* methods. For the homology-based prediction, human, mouse, cattle, and horse proteins were downloaded from Ensembl (release 64) and mapped onto the genome using tblastn. Homologous genome sequences were then aligned against the matching proteins using GeneWise (GeneWise, RRID:SCR_015054) [23] to define gene models. For *de novo* prediction, Augustus (Augustus: Gene Prediction, RRID:SCR_008417) [24], GENSCAN (GENSCAN, RRID:SCR_012902) [25], and SNAP (SNAP, RRID:SCR_007936) [26] were applied to predict coding genes, following previous publications [27]. Finally, homology-based and *de novo*-derived gene sets were merged to form a comprehensive and non-redundant reference gene set using GLEAN [28]. The reference gene set contained 23,125 protein-coding genes (Supplementary Table S3).

To assign functions to the newly annotated genes in the gemsbok genome, we aligned them to SwissProt database using blastp with an (E)- value cutoff of 1 e^−5^. A total of 19,949 genes (86.27% of the total annotated genes) had a SwissProt match. Publicly available databases (including Pfam, PRINTS, PROSITE, ProDom, and SMART) were used to annotate motifs and domains using InterPro, producing 17,112 genes annotated with domain information (74%). By searching the Kyoto Encyclopedia of Genes and Genomes database using a best hit for each gene, 9,696 genes were mapped to a known pathway (41.93% of the genes). Finally, we assigned a gene ontology term to 14,196 genes, representing 61.39% of the whole set. Overall, 20,008 genes (86.52%) had at least one functional annotation (Supplementary Table S3).

### Genome evolution

**Figure 5:**
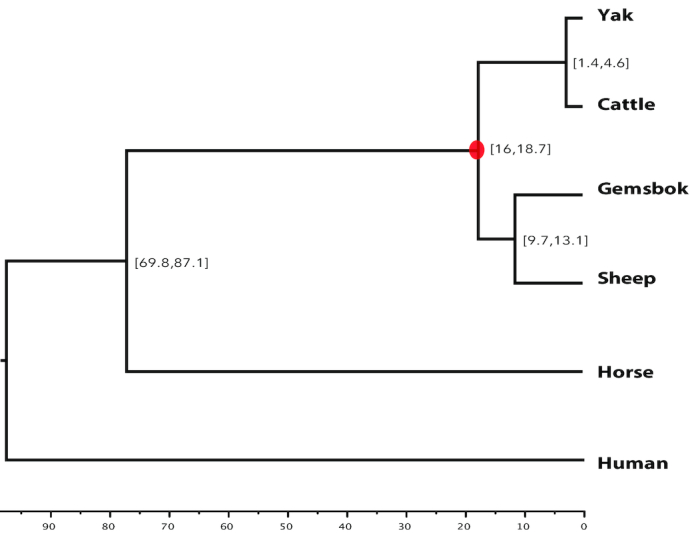
Phylogenetic relationships of gemsbok. Phylogenetic tree constructed with orthologous genes. Divergence times were extracted from the TimeTree database for calibration. Numbers in brackets indicate the estimated diverge times in millions of years, and red circle indicates the calibration time.

To understand the evolution of gemsbok, we reconstructed phylogenetic relationships within the bovid and ruminant clade. To do so, we first used the TreeFam methodology [29] to define gene families in six mammalian genomes using newly defined or existing gene annotations (cattle, sheep, gemsbok, yak, horse, and human) following previous publications [30]. A total of 16,148 gene families were identified, of which 1,327 are single-copy orthologs. The single-copy families were used to reconstruct the phylogenetic tree of the six mammals mentioned above. Concatenated protein sequence alignments were used as input for building the tree, with the JTT+gamma model using PhyMLv3.3 [31]. We assessed the branch reliability by using 1,000 bootstrap replicates. To determine divergence times, PAML (PAML, RRID:SCR_014932) mcmctree [32] was used with the approximate likelihood calculation method and data from TimeTree [33]. We found the same tree topology as identified previously [1] (Fig. 5), with gemsbok being more closely related to sheep than to cattle and yak.

## Availability of supporting data

The raw sequence data have been deposited in the Short Read Archive under accession numbers SRR7503154, SRR7503153, SRR7503152, SRR7503151, SRR7503160, SRR7503159, SRR7503135, SRR7503136, SRR7503137, SRR7503138, SRR7503139, and SRR7503140. The SOAPdenovo + Chicago assembly is also available in NCBI under accession number RAWW00000000. Further supporting data, including annotations and RACA PCF reconstructions, are available in the *GigaScience* database, GigaDB [34]. Visualizations of the different assemblies can be found in Supplementary Fig. S1 and in Evolution Highway [35].

## Additional files

**Table S1**. Summary of sequenced libraries for *Oryx gazella*.

**Table S2**. Summary statics of interspersed repeat regions in *Oryx gazella*.

**Table S3**. Summary statistics of function annotation for the predicted protein coding genes.

SupplFigure1.pdf

## Abbreviations

BUSCO: Benchmarking Universal Single-Copy Orthologs; HSB: homologous synteny block; NCBI: National Center for Biotechnology Information; PCF: predicted chromosome fragment; RACA: Reference-Assisted Chromosome Assembly tool; TE: transposable element.

## Competing interests

The authors declare that they have no competing interests.

## Funding

The following funding was provided: US Department of Agriculture Cooperative State Research Education and Extension Service, Livestock Genome Sequencing Initiative (grants 538 AG2009–34 480-19 875 and 538 AG 58–1265-0–03 to H.A.L.), Biotechnology and Biological Sciences Research Council (grant BB/P020062/1 to D.M.L.), and Strategic Priority Research Program of the Chinese Academy of Science (grant XDB13000000 XDPB0202 to G.Z.).

## Author contributions

M.F. performed SOAPdenovo + RACA and SOAPdenovo + Chicago + RACA assemblies, evaluated all of the assemblies, and wrote the manuscript. Q.L. and Y.Z. performed SOAPdenovo genome assembly and gene annotation. L.G.C. and O.A.R. prepared cell cultures and extracted DNA. G.Z. supervised SOAPdenovo assembly and gene annotation. J.K. and J.M. assisted in RACA assemblies. J.D. performed paired-end read mapping. D.M.L. and H.A.L. supervised the project and revised the manuscript.

## Supplementary Material

GIGA-D-18-00375_Original_Submission.pdf

GIGA-D-18-00375_Revision_1.pdf

Response_to_Reviewer_Comments_Original_Submission.pdf

Reviewer_1_Report_Orginal_Submission -- Joshua Moses Miller 10/31/2018 Reviewed

Reviewer_1_Report_Revsion_1 -- Joshua Moses Miller 12/7/2018 Reviewed

Reviewer_2_Report_Orginal_Submission -- Aaron Shafer 11/5/2018 Reviewed

Supplemental Files
