# Supplementary material for: A near-chromosome-scale genome assembly of the gemsbok (Oryx gazella): an iconic antelope of the Kalahari desert
Source: Gigascience. 2019 Jan 16;8(2):giy162. doi: 10.1093/gigascience/giy162 (PMC6351727; doi:10.1093/gigascience/giy162)
Supplement: GIGA-D-18-00375_Revision_1.pdf [file giy162_giga-d-18-00375_revision_1.pdf]

GigaScience

A Near-Chromosome Scale Genome Assembly of the Gemsbok (Oryx gazella): An Iconic Antelope of the Kalahari Desert

--Manuscript Draft--

**Manuscript Number:**	GIGA-D-18-00375R1
**Full Title:**	A Near-Chromosome Scale Genome Assembly of the Gemsbok (Oryx gazella): An Iconic Antelope of the Kalahari Desert
**Article Type:**	Data Note
**Funding Information:**	Biotechnology and Biological Sciences Research Council (BB/P020062/1) — Dr Denis M Larkin<br>Agricultural Research Service (538 AG2009-34480-1987) — Prof Harris A Lewin<br>Agricultural Research Service (538 AG 58-1265-0-03) — Prof Harris A Lewin<br>Chinese Academy of Sciences (XDB13000000 XDPB0202) — Dr Guojie Zhang
**Abstract:**	Background. The gemsbok (Oryx gazella) is one of the largest antelopes in Africa. Gemsbok are heterothermic and thus highly adapted to live in the desert, changing their feeding behavior when faced with extreme drought and heat. A high-quality genome sequence of this species will assist efforts to elucidate these and other important traits of gemsbok and facilitate research on conservation efforts. Findings. Using 180 Gbp of Illumina paired-end and mate-pair reads, a 2.9 Gbp assembly with scaffold N50 of 1.48 Mbp was generated using SOAPdenovo. Scaffolds were extended using Chicago library sequencing, which yielded an additional 114.7 Gbp of DNA sequence. The HiRise assembly using SOAPdenovo + Chicago library sequencing produced a scaffold N50 of 47 Mbp and a final genome size of 2.9 Gbp, representing 90.6% of the estimated genome size and including 93.2% of expected genes according to BUSCO analysis. The Reference-Assisted Chromosome Assembly tool (RACA) was used to generate a final set of 47 predicted chromosome fragments with N50 of 86.25 Mbp and containing 93.8% of expected genes. A total of 23,125 protein-coding genes and 1.14 Gbp of repetitive sequences were annotated using de novo and homology-based predictions. Conclusions. Our results provide the first high-quality, chromosome-scale genome sequence assembly for gemsbok, which will be a valuable resource for studying adaptive evolution of this species and other ruminants.
**Corresponding Author:**	Marta Farré<br>Royal Veterinary College<br>UNITED KINGDOM
**Corresponding Author Secondary Information:**	
**Corresponding Author's Institution:**	Royal Veterinary College
**Corresponding Author's Secondary Institution:**	
**First Author:**	Marta Farré
**First Author Secondary Information:**	
**Order of Authors:**	Marta Farré<br>Qiye Li<br>Yang Zhou<br>Joana Damas<br>Leona G. Chemnick<br>Jaebum Kim

Powered by Editorial Manager® and ProduXion Manager® from Aries Systems Corporation

	Oliver A Ryder
	Jian Ma
	Guojie Zhang
	Denis M Larkin
	Harris A Lewin
**Order of Authors Secondary Information:**	
**Response to Reviewers:**	Dear Mr Zauner,<br><br>We are very grateful for your time and the time of two referees who reviewed our manuscript. We were very pleased to find that the paper could be accepted for publication in GigaScience just after some minor modifications.<br><br>Our detailed responses to reviewers' comments are included below.<br><br>We also submit the modified manuscript with all changes highlighted as well as the modified figures, tables and supplementary files.<br><br>Sincerely yours,<br><br>Marta Farré<br>Denis Larkin<br>Harris Lewin<br><br><br>Editor comments: As you will see, both reviewers don't have major issues with the quality of the work, overall, but reviewer 2 doubts whether it is a sufficient advance to merit publication. After some discussion with the editorial team, we feel that it is indeed a borderline case, as usually our data notes present more complete assemblies these days. After some discussion among the editorial team, we sided with the more encouraging advice of reviewer 1. However, we feel that it would be helpful if you can add some additional value to the paper, e.g. by adding the circos plot on chromosome synteny as recommended by reviewer 1.<br><br>Reply: We are very grateful that the editorial team finds our manuscript worth of publication in GigaScience. We have included a circos plot showing the chromosome synteny between gemsbok and cattle assemblies as suggested (Figure 4a).<br><br>Editor comments: In addition to the reviewers' comments, please note that one feature of most of our genome Data Notes is a phylogeny of some related species, to give the readers some impression of the position of the newly sequenced species. If this is feasible, please consider including this as well.<br><br>Reply: We have included a phylogeny showing the relation of our newly sequenced species with other ruminants (cattle, yak and sheep) as well as other mammalian species (horse and human). We added a new section in the manuscript detailing this analysis and its results, and a new figure (Figure 5).<br><br>Editor comments: I also note that you mention that visualizations of the different assemblies will be available via the Evolution Highway site - I didn't manage to access this, please make sure this is accessible when you submit the revised version. I feel it may also be informative to include some of these visualizations (e.g. as screenshots) in the manuscript and briefly discuss them, if it's not redundant with information that is already included in the paper.<br><br>Reply: Evolution Highway now contains all the data, but we have also included screenshots of all the chromosomes as Supplementary Figure 1. Moreover, one Evolution Highway chromosome is now part of Figure 4.<br><br>Editor comments: Please also re-consider the title - in the light of reviewer 2's comment, I feel it is a bit misleading to label the assembly as "chromosome scale".

Reply: We agree with the reviewer that it might be misleading and have modified the title accordingly. It now reads: “A near-chromosome scale Genome Assembly of the Gemsbok (Oryx gazella): An Iconic Antelope of the Kalahari Desert”.

Reviewer #1: In this work Farré and colleagues present a genomic assembly for the gemsbok, and African ungulate with interesting adaptations. Using a combination of sequencing and bioinformatic methods the authors have created a chromosome level assembly with a high content of BUSCO genes. This assembly will serve as a reference for future studies of the unique adaptations of gemsbok compared to other ungulates. Overall, I think this is a well written manuscript that applies the latest techniques for genome assembly and annotation. My major comment would be that I think a circos plot of chromosome synteny in the gemsbok compared to domestic cattle would greatly add to the manuscript. Especially given the amount of attention given to the Reference-Assisted Chromosome Assembly tool.

Reply: We thank Reviewer 1 for their very encouraging comment, and we completely agree with them. We have created a new circos plot showing the chromosome synteny between the new gemsbok assembly and cattle genome, as shown in Figure 4. Moreover, data from Evolution Highway showing a detailed analysis of synteny between gemsbok, cattle and human are now part of Figure 4 and in Supplementary Figure 1.

As such, we have updated the manuscript. The text now reads:
“Finally, we assessed the genome continuity by identifying homologous synteny blocks (HSBs) between gemsbok and cattle chromosomes (Suppl. Fig. 1). Gemsbok (2n = 56) and cattle (2n = 60) karyotypes differ by two Robertsonian translocations [7], but only one of them is present in the gemsbok assembly (Figure 4). A total of 21 cattle chromosomes aligned to an individual gemsbok fragment, indicating that they represent complete gemsbok chromosomes. Eight cattle chromosomes (BTA1, BTA3, BTA4, BTA11, BTA16, BTA22, BTA28, and BTAX) were syntenic to two or more gemsbok HSBs, suggesting that these HSBs represent chromosomal fragments. The HSBs were physically-assigned to chromosomes based on known syntenic relationships to cattle chromosomes [7].”

Reviewer #1: Along this line, I noted that in the introduction it is stated that gemsbok are predicted to have 56 chromosomes, but the final assembly only contains 47. Can the authors comment on this discrepancy? Are these "remaining" chromosomes especially small?

Reply: We thank the reviewer for this comment. Indeed, gemsbok have a diploid number (2n) of 56 chromosomes, representing two copies of each of the 28 unique chromosomes that would be expected in the assembly. Our assembly consists of 47 chromosomal fragments, 21 of them representing entire gemsbok chromosomes, and 8 gemsbok chromosomes assembled into two or more fragments, the latter accounting for the difference from the expected 28.

Reviewer #2: The paper presents a genome assembly of the Gemsbok. The methodology is standard, and the genome is of reasonably good quality. I do not have any concerns other than to say the article presents common analyses that many groups, my group included, regularly conduct and do not publish as it is common to mix technologies and assembly strategies. Moreover, there were "problematic" scaffolds which in normal, but as per Data Notes guidelines, I do not consider this to be an exceptional data set rather a now commonplace non-model organism genome. The chromosome level highlighted in the title is presumably based off of some reference genome (never mentioned but required for RACA?), thus the chromosome order is based off a distant relative, compared to something identified with long-reads, where the latter would truly be an "exceptional" data set as per GigaScience guidelines.

Powered by Editorial Manager® and ProduXion Manager® from Aries Systems Corporation

	Reply: We thank Reviewer 2 for their time in revising our manuscript. We agree that with new technologies it has now become commonplace to sequence and assemble non-model organisms; however, we believe that the implementation of RACA as an evaluation method will be a powerful approach to assist in the assembly and assess the quality of genome assemblies obtained using third generation methodologies. Moreover, having a genome assembled at near-chromosome level will foster research into the unique adaptations that gemsbok has, as well as helping studies of endangered and closely related species, such as the scimitar oryx, for which a high-quality genome is still not available.<br>As suggested by the reviewer, we have modified the title of the manuscript. It now reads: “A Near-Chromosome Scale Genome Assembly of the Gemsbok (Oryx gazella): An Iconic Antelope of the Kalahari Desert”.<br><br>Gemsbok chromosome assignment was done using synteny comparison to cattle chromosomes and following the publication where gemsbok karyotype was established (Gallagher & Womack 1992). This has now been incorporated in the text, and it reads: “The HSBs were physically-assigned to chromosomes based on known syntenic relationships to cattle chromosomes [7].”
**Additional Information:**	
**Question**	**Response**
Are you submitting this manuscript to a special series or article collection?	No
**Experimental design and statistics**<br><br>Full details of the experimental design and statistical methods used should be given in the Methods section, as detailed in our Minimum Standards Reporting Checklist. Information essential to interpreting the data presented should be made available in the figure legends.<br><br>Have you included all the information requested in your manuscript?	Yes
**Resources**<br><br>A description of all resources used, including antibodies, cell lines, animals and software tools, with enough information to allow them to be uniquely identified, should be included in the Methods section. Authors are strongly encouraged to cite Research Resource Identifiers (RRIDs) for antibodies, model organisms and tools, where possible.<br><br>Have you included the information requested as detailed in our Minimum	Yes

Powered by Editorial Manager® and ProduXion Manager® from Aries Systems Corporation

Standards Reporting Checklist?	
**Availability of data and materials**<br><br>All datasets and code on which the conclusions of the paper rely must be either included in your submission or deposited in publicly available repositories (where available and ethically appropriate), referencing such data using a unique identifier in the references and in the “Availability of Data and Materials” section of your manuscript.<br><br>Have you have met the above requirement as detailed in our Minimum Standards Reporting Checklist?	Yes

Manuscript

Click here to view linked References

Click here to access/download;Manuscript;Farre_gemsbok_final_11_12_18.

A Near-Chromosome-Scale Genome Assembly of the Gemsbok (*Oryx gazella*): An Iconic Antelope of the Kalahari Desert

Marta Farré[1,#,*], Qiye Li[2,3,#], Yang Zhou[3,4], Joana Damas[1], Leona G. Chemnick[5], Jaebum Kim[6], Oliver A. Ryder[5], Jian Ma[7], Guojie Zhang[2,3,4], Denis M. Larkin[1,*] and Harris A. Lewin[8,*]

1. Comparative Biomedical Sciences Department, Royal Veterinary College, University of London, UK.
2. State Key Laboratory of Genetic Resources and Evolution, Kunming Institute of Zoology, Chinese Academy of Sciences, Kunming 650223, China
3. China National Genebank, BGI-Shenzhen, Shenzhen 518120, China
4. Centre for Social Evolution, Department of Biology, Universitetsparken 15, University of Copenhagen, DK-2100 Copenhagen, Denmark.
5. San Diego Zoo Institute for Conservation Research, Escondido, California, USA.
6. Department of Biomedical Science and Engineering, Konkuk University, Seoul 05029, South Korea.
7. Computational Biology Department, School of Computer Science, Carnegie Mellon University, USA.
8. The UC Davis Genome Center, Department of Evolution and Ecology, College of Biological Sciences, and the Department of Reproduction and Population Health, School of Veterinary Medicine, University of California, Davis, USA.

[#] These authors contributed equally
* Corresponding authors

Emails:
M.F.: mfarrebelmonte@gmail.com
J.K.: jaebum.kim@gmail.com
J.D.: joanadamas@gmail.com
Q.L.: liqiye@genomics.cn
Y.Z.: zhouyang@genomics.cn
L.G.C.: lchemnick@sandiegozoo.org
O.R.: oryder@sandiegozoo.org
J.M.: jianma@cs.cmu.edu
G.Z.: zhanggj@genomics.cn
D.M.L.: dlarkin@rvc.ac.uk
H.A.L.: lewin@ucdavis.edu

Abstract

Background. The gemsbok (*Oryx gazella*) is one of the largest antelopes in Africa. Gemsbok are heterothermic and thus highly adapted to live in the desert, changing their feeding behavior when faced with extreme drought and heat. A high-quality genome sequence of this species will assist efforts to elucidate these and other important traits of gemsbok and facilitate research on conservation efforts. **Findings.** Using 180 Gbp of Illumina paired-end and mate-pair reads, a 2.9 Gbp assembly with scaffold N50 of 1.48 Mbp was generated using SOAPdenovo. Scaffolds were extended using Chicago library sequencing, which yielded an additional 114.7 Gbp of DNA sequence. The HiRise assembly using SOAPdenovo + Chicago library sequencing produced a scaffold N50 of 47 Mbp and a final genome size of 2.9 Gbp, representing 90.6% of the estimated genome size and including 93.2% of expected genes according to BUSCO analysis. The Reference-Assisted Chromosome Assembly tool (RACA) was used to generate a final set of 47 predicted chromosome fragments with N50 of 86.25 Mbp and containing 93.8% of expected genes. A total of 23,125 protein-coding genes and 1.14 Gbp of repetitive sequences were annotated using *de novo* and homology-based predictions. **Conclusions.** Our results provide the first high-quality, chromosome-scale genome sequence assembly for gemsbok, which will be a valuable resource for studying adaptive evolution of this species and other ruminants.

Keywords: gemsbok, *Oryx gazella*, assembly, annotation, ruminant, drought

Background information

The Gemsbok (*Oryx gazella*) is the largest antelope in the genus *Oryx*, and a member of the Hippotraginae tribe of ruminants [1] (Figure 1). The gemsbok's biogeographical distribution includes Botswana and Namibia, traditionally inhabiting the Kalahari and Karoo Deserts in Southern Africa [2]. The climate of these regions is highly seasonal, with cool winters (10°C – 15°C) and hot summers (43°C – 46°C) when most of the annual rainfall occurs (90 – 100 mm). High evaporation rates and low precipitation result in a semi-arid climate in both deserts [3]. Living in such extreme environments, gemsbok have evolved to be highly adapted to drought and extreme heat by minimizing water demand and loss. All the species in the *Oryx* genus are heterotherms, i.e., they can increase their body temperature from ~36°C to ~45°C in order to delay evaporative cooling [4]. *Oryx* species can also change their feeding behavior from grazing to browsing and digging when faced by extreme

environmental conditions [5]. Male and female gemsbok are characterized by their low sexual dimorphism, with both sexes having horns and other shared secondary sexual traits [6], making them highly sought after by trophy hunters.

The gemsbok karyotype has 2n=56 chromosomes, with two Robertsonian translocations compared to cattle [7]. Gemsbok populations have high genetic diversity [8], consistent with other African bovids [9, 10]. Here we report a chromosome-scale gemsbok genome sequence that will be useful for elucidating the unique adaptations that allow gemsbok to live in arid climates. Several of the large scaffolds are chromosome-length or near chromosome-length, which will facilitate detailed studies of genome evolution in ruminants. The high quality, chromosome scale assembly of the gemsbok contribute to the goals of the Genome 10K Project [11] and the Earth BioGenome Project [12].

Data description

Library construction, sequencing and filtering

Genomic DNA was extracted from a captive born female Gemsbok from San Diego Safari Park (USA) using heart muscle collected at necropsy (NCBI BioSample ID SAMN09604855). High-molecular weight genomic DNA was obtained using the phenol/chloroform protocol as previously described [13]. Isolated genomic DNA was then used to construct four short-insert sequencing libraries (170, 250, 500, and 800 bp) and eight long-insert libraries (2 Kbp x 2, 5 Kbp x 2, 10 Kbp x 2, and 20 Kbp x 2) following standard protocols provided by Illumina (San Diego, CA, USA). Then, sequencing of the short- and long-insert size libraries was performed using the Illumina Hiseq 2000 platform to generate 301.39 Gbp of raw data (Supplementary Table 1). Reads were trimmed based on low base quality, and reads with more than 5% of uncalled (“N”) bases were removed, providing a total of 179.64 Gbp of filtered read data for genome assembly.

Two Chicago libraries were generated (Dovetail Genomics, Santa Cruz, CA) as previously described [14]. Briefly, high-molecular-weight DNA was assembled into chromatin *in vitro* and then chemically cross-linked before being restriction digested. The overhangs were filled in with a biotinylated nucleotide, and the chromatin was incubated in a proximity-ligation reaction. The cross-links were then reversed, and the DNA purified from chromatin. After sequencing these libraries on the Illumina Hiseq 4000 platform, we obtained ~382 million 150 bp read pairs.

Evaluation of genome size

We used k-mer analysis to estimate the size of gemsbok’s genome. A k-mer refers to an artificial sequence division of K nucleotides iteratively from sequencing reads. A raw sequence read

with L bp contains (L-K+1) k-mers if the length of each k-mer is K bp. The frequency of each k-mer can be calculated from the genome sequence reads. Typically, k-mer frequencies plotted against the sequence depth gradient follow a Poisson distribution in any given dataset, whereas sequencing errors may lead to a higher representation of low frequencies. The genome size, G, can then be calculated from the formula G=K_num/K_depth, where the K_num is the total number of k-mer, and K_depth denotes the depth of coverage of the k-mer with the highest frequency. In gemsbok, K was 17, K_num was 85,155,457,485 and the K_depth was 26. Therefore, we estimated the genome size of *Oryx gazella* to be 3.2 Gbp. The filtered reads provided approximately 61.9-fold mean coverage of the genome, while the Chicago library represented 72.7-fold genome coverage.

Genome assembly

We used SOAPdenovo, version 2.04, (SOAP, RRID:SCR_000689) to construct contigs and scaffolds following previously published protocols [15]. The gemsbok genome assembly was 2.90 Gbp long, including 177.88 Mbp (6.13%) of unknown bases. The contig N50 and scaffold N50 sizes were 17.25 Kbp and 1.48 Mbp, respectively (Table 1, Figure 2a). To assess assembly quality, approximately 98 Gbp (representing genome coverage of 34x) high quality short-insert size reads were aligned to the assembly using Burrows-Wheeler Aligner (BWA, RRID:SCR_010910), with parameters of -t 1 -I [16]. A total of 95.3% reads could be mapped, covering 97.8% of the assembly excluding gaps; 82.1% of these reads were properly paired with an expected insert size associated with the different libraries.

To increase the contiguity of the assembly we used sequence information from the Chicago libraries and the HiRise (version 2.0) scaffolder (Figure 2a) [14]. A total of 5,411 new joins were produced, resulting in a superscaffold N50 of 47.03 Mbp (Table 1).

In parallel, we assembled the gemsbok genome with the Reference-Assisted Chromosome Assembly tool (RACA) [17] using the original SOAPdenovo assembly and raw sequence reads as input (Figure 2a). Using comparative genomic information and paired-end read mapping to target genome scaffolds, RACA orders and orients scaffolds of a target species into predicted chromosome fragments (PCFs). Only scaffolds longer than 10 Kbp were included in the assembly, accounting for 95% of its length. The cattle (bosTau6) and human (hg19) genomes were used as reference and outgroup, respectively, and all the Illumina paired-end and mate-pair libraries were used in the RACA assembly. Briefly, read libraries were aligned to SOAPdenovo scaffolds using Bowtie2, and syntenic fragments (SFs) were constructed at 150 Kbp resolution after aligning cattle and gemsbok scaffolds using lastZ and UCSC Kent utilities [18] as previously described [17, 19]. A total of 49 PCFs were reconstructed, of which 21 were homologous to complete cattle chromosomes, and a final PCF

N50 of 80.57 Mbp was achieved (Table 1). More than 97% of the scaffold joins introduced in the SOAPdenovo + Chicago assembly were concordant with the RACA assembly, showing a high agreement between both methodologies.

Evaluation of SOAPdenovo assembly

To further evaluate the structure of the SOAPdenovo scaffolds we used the information provided by RACA (Figure 2b). The RACA evaluation allowed identification of problematic regions in scaffolds with low read physical coverage and not supported by syntenic information from either the reference and the outgroup genomes. As we previously showed [17, 19], 20 to 60 percent of the flagged problematic scaffolds are chimeric and, therefore, not existent in the genome. In gemsbok, only 12 SOAPdenovo scaffolds were identified as putatively chimeric after running RACA (Table 1).

The HiRise assembler also pinpointed putatively chimeric SOAPdenovo scaffolds using the Chicago libraries sequence information (Figure 2b). A total of 17 regions in 16 SOAPdenovo scaffolds were identified in this manner. Among the 16 problematic SOAPdenovo scaffolds identified using Chicago library sequence information, four were also flagged by RACA, while four SOAPdenovo scaffolds were not included in the RACA assembly because they were smaller than 10 Kbp. Seven SOAPdenovo scaffolds were broken in the SOAPdenovo + Chicago assembly, but one of the fragments was below the 150 Kbp resolution chosen to run RACA and therefore not reported in the RACA output. Only two complete disagreements between the SOAPdenovo + Chicago and SOAPdenovo + RACA assemblies were identified.

Evaluation of SOAPdenovo + Chicago assembly

To assess the SOAPdenovo + Chicago assembly, RACA was used to identify putative chimeric superscaffolds (Figure 2b). Because there is no physical or genetic map for gemsbok, we were not able to verify the scaffold adjacencies in PCFs predicted by RACA, and therefore, the PCFs were used as a tool to evaluate the SOAPdenovo + Chicago assembly. In this assessment, cattle and human genomes served as the reference and outgroup, respectively, and the SOAPdenovo + Chicago assembly as input. A total of 47 PCFs were reconstructed with N50 of 86.25 Mbp (Table 1), representing 94.5% of the original SOAPdenovo assembly. Nineteen PCFs were orthologous to complete cattle chromosome. Two PCFs corresponding to one complete cattle chromosome were fused to fragments of other chromosomes, and 17 PCFs representing complete independent chromosomes. One PCF represented the complete cattle chromosome 3 in the SOAPdenovo + RACA assembly, while in the SOAPdenovo + Chicago + RACA it was broken into two pieces corresponding to the region with the lowest adjacency score in the SOAPdenovo + RACA assembly. Another PCF

was orthologous to cattle chromosome 11, but in the new assembly it was fragmented into two PCFs, one of ~186 Kbp containing sequence not present in the SOAPdenovo + RACA assembly.

More than 98% of the scaffold joins introduced in the SOAPdenovo + Chicago assembly were consistent with RACA results and are thus likely to be accurate. However, RACA introduced 50 breaks in 25 SOAPdenovo + Chicago scaffolds, suggesting that these scaffolds might be chimeric (Figure 2b). Of the 50 breaks, 27 comprised joins of SOAPdenovo scaffolds into superscaffolds made using the HiRise assembler. The other 23 breaks were inside single SOAPdenovo scaffolds, with five being also broken in the SOAPdenovo + RACA assembly, while the rest were either not used (4 cases) or below the 150 Kbp resolution of the SOAPdenovo + RACA assembly (14 cases). Although physical or genetic maps for gemsbok are not available to verify the SOAPdenovo + Chicago + RACA assembly, we previously showed that RACA produces highly accurate chromosome assemblies when compared to meiotic linkage [20] or cytogenetic physical maps [19], suggesting that the 47 PCFs of the gemsbok assembly accurately represent scaffold order and orientation on the gemsbok chromosomes. Therefore, using RACA allowed us to identify putatively chimeric scaffolds and superscaffolds, as well as to align components of chimeric scaffolds to their likely location on the gemsbok genome.

Genome completeness was assessed using the Benchmarking Universal Single-Copy Orthologs (BUSCO, RRID:SCR_015008) [21]) software, version 3.0. More than 92% of the core mammalian gene set was complete in all the assemblies (Figure 3), with the SOAPdenovo + Chicago + RACA assembly being the most complete, containing 96.3% of the gene set with 93.8% being complete. The percentage of complete genes in this assembly is similar to other recent ruminant assemblies (93.8% and 94.1% in goat ARS1 and cattle ARS-UCD1.2, respectively, Fig. 3), showing that the Gemsbok SOAPdenovo + Chicago + RACA assembly is of similar quality. Finally, we assessed the genome continuity by identifying homologous synteny blocks (HSBs) between gemsbok and cattle chromosomes (Suppl. Fig. 1). Gemsbok (2n = 56) and cattle (2n = 60) karyotypes differ by two Robertsonian translocations [7], but only one of them is present in the gemsbok assembly (Figure 4). A total of 21 cattle chromosomes aligned to an individual gemsbok fragment, indicating that they represent complete gemsbok chromosomes. Eight cattle chromosomes (BTA1, BTA3, BTA4, BTA11, BTA16, BTA22, BTA28, and BTAX) were syntenic to two or more gemsbok HSBs, suggesting that these HSBs represent chromosomal fragments. The HSBs were physically-assigned to chromosomes based on known syntenic relationships to cattle chromosomes [7].

Genome annotation

To annotate the gemsbok genome, we started by mapping transposable elements (TEs). The

TEs were predicted in the genome by homology to RepBase sequences using RepeatProteinMask and RepeatMasker (RepeatMasker, RRID:SCR_012954) [22] with default parameters, then the results were combined to produce a non-redundant final set. About 42.5% of the gemsbok genome is comprised of TEs, with LINEs being the most frequent class (25.71%, Supplementary Table 2).

The rest of the genome assembly was annotated using both homology-based and *de novo* methods. For the homology-based prediction, human, mouse, cattle, and horse proteins were downloaded from Ensembl (release 64) and mapped onto the genome using tblastn. Homologous genome sequences were then aligned against the matching proteins using GeneWise (GeneWise, RRID:SCR_015054) [23] to define gene models. For *de novo* prediction, Augustus (Augustus: Gene Prediction, RRID:SCR_008417) [24], GENSCAN (GENSCAN, RRID:SCR_012902) [25], and SNAP (SNAP, RRID:SCR_007936) [26] were applied to predict coding genes, following previous publications [27]. Finally, homology-based and *de novo* derived gene sets were merged to form a comprehensive and non-redundant reference gene set using GLEAN [28]. The reference gene set contained 23,125 protein coding genes (Supplementary Table 3).

To assign functions to the newly annotated genes in the gemsbok genome, we aligned them to SwissProt database using blastp with an (E)- value cutoff of 1 e^{-5}. A total of 19,949 genes (86.27% of the total annotated genes) had a Swissprot match. Publicly available databases (including Pfam, PRINTS, PROSITE, ProDom, and SMART) were used to annotate motifs and domains using InterPro, producing a total of 17,112 genes annotated with domain information (74%). By searching the KEGG database using a best hit for each gene, 9,696 genes were mapped to a known pathway (41.93% of the genes). Finally, we assigned a gene ontology term to 14,196 genes, representing 61.39% of the whole set. Overall, 20,008 genes (86.52%) had at least one functional annotation (Supplementary Table 3).

Genome evolution

To understand the evolution of gemsbok, we reconstructed phylogenetic relationships within the bovid and ruminant clade. To do so, we first used the TreeFam methodology [29] to define gene families in six mammalian genomes using newly defined or existing gene annotations (cattle, sheep, gemsbok, yak, horse, and human) following previous publications [30]. A total of 16,148 gene families were identified, of which 1,327 are single-copy orthologs. The single-copy families were used to reconstruct the phylogenetic tree of the six mammals mentioned above. Concatenated protein sequence alignments were used as input for building the tree, with the JTT+gamma model using PhyMLv3.3 [31]. We assessed the branch reliability by using 1,000 bootstrap replicates. To determine divergence times, PAML (PAML, RRID:SCR_014932) mcmctree [32] was used with the

approximate likelihood calculation method and data from TimeTree [33]. We found the same tree topology as identified previously [1] (Fig. 5), with gemsbok being more closely related to sheep than to cattle and yak.

List of abbreviations

BUSCO: Benchmarking Universal Single-Copy Orthologs; RACA: Reference Assisted Chromosome Assembly; PCF: Predicted Chromosome Fragment.

Availability of supported data

The raw sequence data have been deposited in the Short Read Archive (SRA) under accession numbers SRR7503154, SRR7503153, SRR7503152, SRR7503151, SRR7503160, SRR7503159, SRR7503135, SRR7503136, SRR7503137, SRR7503138, SRR7503139, SRR7503140. The SOAPdenovo + Chicago assembly is also available in NCBI under accession number (RAWW00000000). Further supporting data, including annotations and RACA PCF reconstructions, are available in the *GigaScience* database, GigaDB [34]. Visualizations of the different assemblies can be found in Supplementary Figure 1 and in Evolution Highway [35].

Competing interests

The authors declare that they have no competing interests.

Funding

US Department of Agriculture Cooperative State Research Education and Extension Service, Livestock Genome Sequencing Initiative Grants 538 AG2009-34480-19875 and 538 AG 58-1265-0-03 (to H.A.L). Biotechnology and Biological Sciences Research Council Grant BB/P020062/1 (to D.M.L.). Strategic Priority Research Program of the Chinese Academy of Science Grant No. XDB13000000 XDPB0202 (to G.Z.).

Author contributions

M.F. performed SOAPdenovo + RACA and SOAPdenovo + Chicago + RACA assemblies, evaluated all the assemblies and wrote the manuscript. Q.L. and Y.Z. performed SOAPdenovo genome assembly and gene annotation. L.G.C. and O.A.R. prepared cell cultures and extracted DNA. G.Z. supervised SOAPdenovo assembly and gene annotation. J.K. and J.M. assisted in RACA assemblies. J.D.

performed paired-end read mapping. D.M.L. and H.A.L. supervised the project and revised the manuscript.

References

1. Decker JE, Pires JC, Conant GC, McKay SD, Heaton MP, Chen K, et al. Resolving the evolution of extant and extinct ruminants with high-throughput phylogenomics. Proc Natl Acad Sci U S A. 2009;106 44:18644-9. doi:10.1073/pnas.0904691106.
2. Knight M. Oryx gazella Gemsbok (Southern Oryx). In: Kingdon J and Hoffmann M, editors. The Mammals of Africa VI Pigs, Hippopotamuses, Chevrotain, Giraffes, Deer and Bovids. London: Bloomsbury Publishing; 2013. p. 572-6.
3. Thomas D and Shaw PA. The Kalahari Environment. Cambridge Univsersity Press; 2010.
4. Hetem RS, Maloney SK, Fuller A and Mitchell D. Heterothermy in large mammals: inevitable or implemented? Biol Rev Camb Philos Soc. 2016;91 1:187-205. doi:10.1111/brv.12166.
5. Mills G and Hes L. The Complete Book of Southern African Mammals. Cape Town, South Africa: Struik Publishers (Pty) Ltd; 1997.
6. Estes RD. The behavior guide to African mammals. Berkeley: The University of California Press; 1991.
7. Gallagher DS and Womack JE. Chromosome conservation in the Bovidae. J Hered. 1992;83 4:287-98.
8. Osmers B, Petersen B-S, Hartl GB, Grobler JP, Kotze A, Van Aswegen E, et al. Genetic analysis of southern African gemsbok (Oryx gazella) reveals high variability, distinct lineages and strong divergence from the East African Oryx beisa. Mammalian Biology - Zeitschrift für Säugetierkunde. 2012;77 1:60-6. doi:https://doi.org/10.1016/j.mambio.2011.08.003.
9. Arctander P, Kat PW, Aman RA and Siegismund HR. Extreme genetic differences among populations of Gazella granti, Grant's gazelle in Kenya. Heredity (Edinb). 1996;76 (Pt 5):465-75.
10. Birungi J and Arctander P. Large sequence divergence of mitochondrial DNA genotypes of the control region within populations of the african antelope, kob (Kobus kob). Mol Ecol. 2000;9 12:1997-2008.
11. Koepfli K-P, Paten B, Genome KCoS and O'Brien SJ. The Genome 10K Project: A Way Forward. Annu Rev Anim Biosci. 2015;3:57-111. doi:10.1146/annurev-animal-090414-014900.
12. Lewin HA, Robinson GE, Kress WJ, Baker WJ, Coddington J, Crandall KA, et al. Earth BioGenome Project: Sequencing life for the future of life. Proc Natl Acad Sci U S A. 2018;115 17:4325-33. doi:10.1073/pnas.1720115115.
13. Zhang G, Li C, Li Q, Li B, Larkin DM, Lee C, et al. Comparative genomics reveals insights into avian genome evolution and adaptation. Science. 2014;346 6215:1311-20. doi:10.1126/science.1251385.
14. Putnam NH, O'Connell BL, Stites JC, Rice BJ, Blanchette M, Calef R, et al. Chromosome-scale shotgun assembly using an in vitro method for long-range linkage. Genome Res. 2016;26 3:342-50. doi:10.1101/gr.193474.115.

15. Luo R, Liu B, Xie Y, Li Z, Huang W, Yuan J, et al. SOAPdenovo2: an empirically improved memory-efficient short-read de novo assembler. Gigascience. 2012;1 1:18. doi:10.1186/2047-217x-1-18.
16. Li H and Durbin R. Fast and accurate short read alignment with Burrows-Wheeler transform. Bioinformatics. 2009;25 14:1754-60. doi:10.1093/bioinformatics/btp324.
17. Kim J, Larkin DM, Cai Q, Cai Q, Zhang Y, Ge R-L, et al. Reference-assisted chromosome assembly. Proc Natl Acad Sci U S A. 2013;110 5:1785-90. doi:10.1073/pnas.1220349110.
18. Kent WJ, Sugnet CW, Furey TS, Roskin KM, Pringle TH, Zahler AM, et al. The human genome browser at UCSC. Genome Res. 2002;12 6:996-1006. doi:10.1101/gr.229102. Article published online before print in May 2002.
19. Damas J, O'Connor R, Farre M, Lenis VP, Martell HJ, Mandawala A, et al. Upgrading short read animal genome assemblies to chromosome level using comparative genomics and a universal probe set. Genome Res. 2016; doi:10.1101/gr.213660.116.
20. Rando H, Farré M, Robson M, Won N, Johnson J, Buch R, et al. Construction of Red Fox Chromosomal Fragments from the Short-Read Genome Assembly. Genes. 2018;9 6:308.
21. Simao FA, Waterhouse RM, Ioannidis P, Kriventseva EV and Zdobnov EM. BUSCO: assessing genome assembly and annotation completeness with single-copy orthologs. Bioinformatics. 2015;31 19:3210-2. doi:10.1093/bioinformatics/btv351.
22. Tarailo-Graovac M and Chen N. Using RepeatMasker to identify repetitive elements in genomic sequences. Curr Protoc Bioinformatics. 2009;Chapter 4:Unit 4.10. doi:10.1002/0471250953.bi0410s25.
23. Birney E, Clamp M and Durbin R. GeneWise and Genomewise. Genome Res. 2004;14 5:988-95. doi:10.1101/gr.1865504.
24. Stanke M, Keller O, Gunduz I, Hayes A, Waack S and Morgenstern B. AUGUSTUS: ab initio prediction of alternative transcripts. Nucleic Acids Res. 2006;34 Web Server issue:W435-9. doi:10.1093/nar/gkl200.
25. Burge C and Karlin S. Prediction of complete gene structures in human genomic DNA. J Mol Biol. 1997;268 1:78-94. doi:10.1006/jmbi.1997.0951.
26. Korf I. Gene finding in novel genomes. BMC Bioinformatics. 2004;5:59. doi:10.1186/1471-2105-5-59.
27. Zhang C, Chen L, Zhou Y, Wang K, Chemnick LG, Ryder OA, et al. Draft genome of the milu (Elaphurus davidianus). GigaScience. 2018;7 2:gix130-gix. doi:10.1093/gigascience/gix130.
28. Elsik CG, Mackey AJ, Reese JT, Milshina NV, Roos DS and Weinstock GM. Creating a honey bee consensus gene set. Genome Biol. 2007;8 1:R13. doi:10.1186/gb-2007-8-1-r13.
29. Li H, Coghlan A, Ruan J, Coin LJ, Hériché J-K, Osmotherly L, et al. TreeFam: a curated database of phylogenetic trees of animal gene families. Nucleic Acids Res [Internet]. 2006;34:D572-80. Available from: http://dx.doi.org/10.1093/nar/gkj118
30. Kim EB, Fang X, Fushan AA, Huang Z, Lobanov A V, Han L, et al. Genome sequencing reveals insights into physiology and longevity of the naked mole rat. Nature [Internet]. 2011;479:223–7. Available from: http://dx.doi.org/10.1038/nature10533
31. Guindon S, Dufayard J-F, Lefort V, Anisimova M, Hordijk W, Gascuel O, et al. New Algorithms and Methods to Estimate Maximum-Likelihood Phylogenies: Assessing the Performance of PhyML 3.0 [Internet]. Available from: http://www.lirmm.fr/~gascuel

32. Yang Z, Rannala B. Molecular phylogenetics: principles and practice. Nat Rev Genet [Internet]. 2012;13:303–14. Available from: http://dx.doi.org/10.1038/nrg3186
33. Hedges SB, Dudley J, Kumar S. TimeTree: a public knowledge-base of divergence times among organisms. Bioinformatics [Internet]. 2006 [cited 2018 Nov 19];22:2971–2. Available from: http://www.ncbi.nlm.nih.gov/pubmed/17021158
34. Farré M, Li Q, Zhou Y, Damas J, Chemnick LG, Kim J, Ryder OA et al. Supporting data for "A Near-Chromosome-Scale Genome Assembly of the Gemsbok (Oryx gazella): An Iconic Antelope of the Kalahari Desert". GigaScience Database 2018. http://dx.doi.org/10.5524/100544
35. Evolution Highway. Comparative genomics. http://eh-demo.ncsa.uiuc.edu/ruminants Accessed 7 Dec. 2018.

Table 1. Assembly statistics of *Oryx gazella* genome.

	SOAPdenovo	SOAPdenovo + Chicago	SOAPdenovo + RACA	SOAPdenovo + Chicago + RACA
**Input assembly**	NA	SOAPdenovo	SOAPdenovo	SOAPdenovo + Chicago
**Total length (Mbp)**	2,900.52	2,905.93	2,648.75	2,740.44
**N50 (Mbp)**	1.48	47.03	80.57	86.25
**No. scaffolds/PCFs**	1,223,903	1,218,509	49	47
**No. input scaffolds broken**	--	16	12	25

Figure 1. Picture of a gemsbok (*Oryx gazella*) male at Etosha National Park (Namibia). Picture from Charles J Sharp QS:P170,Q54800218, Gemsbok (Oryx gazella) male, CC BY-SA 4.0

Figure 2. Overview of the approach to generate a chromosome level gemsbok genome assembly. A. Illumina paired-end and mate-pair reads were assembled into contigs (purple) and then into scaffolds (green) using SOAPdenovo (i). These scaffolds were merged into superscaffolds (orange) using Dovetail Chicago methodology (ii) [11]. Finally, RACA [13] was applied to produce chromosomal fragments (blue) from the superscaffolds (iii). **B.** To reveal potential chimeric scaffolds, we used the information provided by RACA to identify regions with low read coverage and no syntenic information (demarcated with a red box) in scaffolds (i) or in superscaffolds (iii). The HiRise scaffolder used Chicago libraries sequencing data to pinpoint potentially chimeric regions (shown in the red box) with low read coverage and a substantial reduction of link support (ii). R: reference, T: target and O: outgroup genomes**.**

Figure 3. Genome assembly evaluation. The BUSCO dataset of the mammalia_odb9 including 4,104 BUSCOs was used to assess the four assemblies and compared to goat and cattle ARS-UCD1.2.

Figure 4. Syntenic relationships between gemsbok and cattle genomes. A. Circos plot showing syntenic relationships between cattle autosomes (labelled as BTA) and gemsbok chromosomal fragments. Chromosomes are colored based on cattle homologies. Ribbons inside the plot show syntenic relationships, while lines inside each ribbon indicate inversions. B. Gemsbok chromosome 15 showing homologous synteny blocks (HSBs) between gemsbok, cattle, and human. SOAPdenovo + Chicago scaffolds are also displayed. The other gemsbok chromosomes can be found in Supplementary Figure 1.

Figure 5. Phylogenetic relationships of gemsbok. Phylogenetic tree constructed with orthologous genes. Divergence times were extracted from the TimeTree database for calibration. Numbers in brackets indicate the estimated diverge times in millions of years (Mya), and red circle indicates the calibration time.

Figure1

Click here to access/download;Figure;Figure1_Gemsbok_(Oryx_gazella)_male.jpg

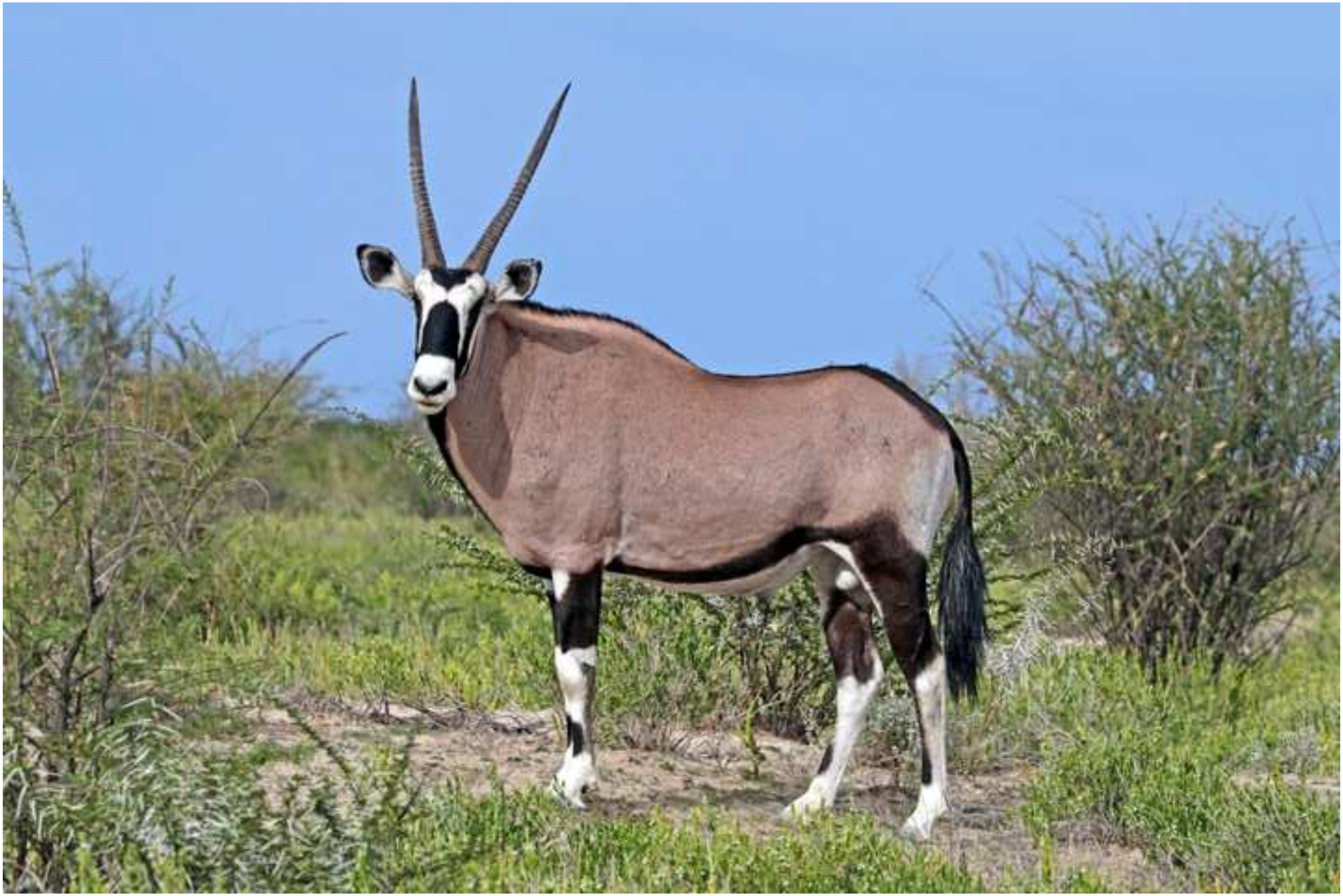

Figure2

Click here to access/download;Figure;Figure2.pdf

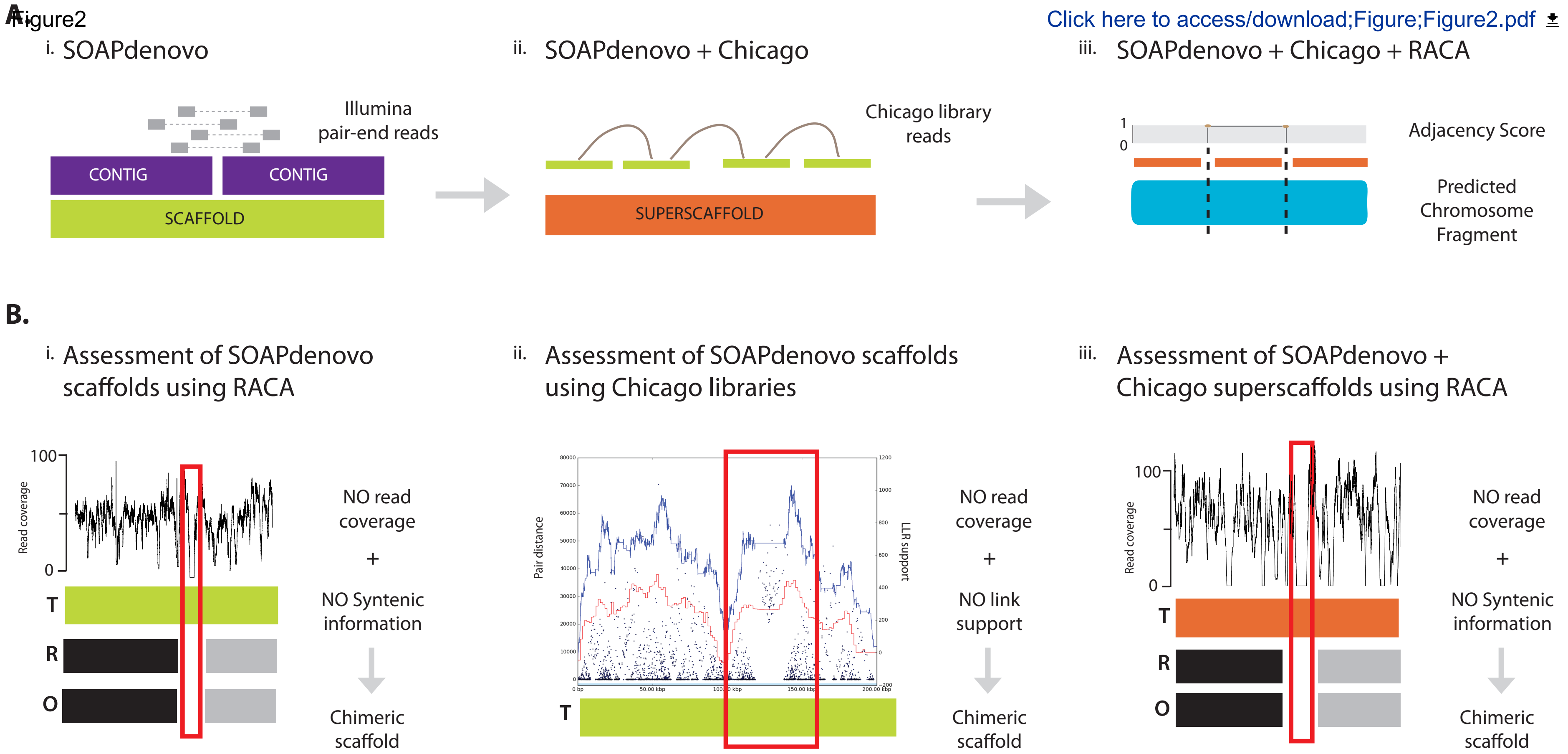

Figure3

Click here to access/download;Figure;Figure3.pdf

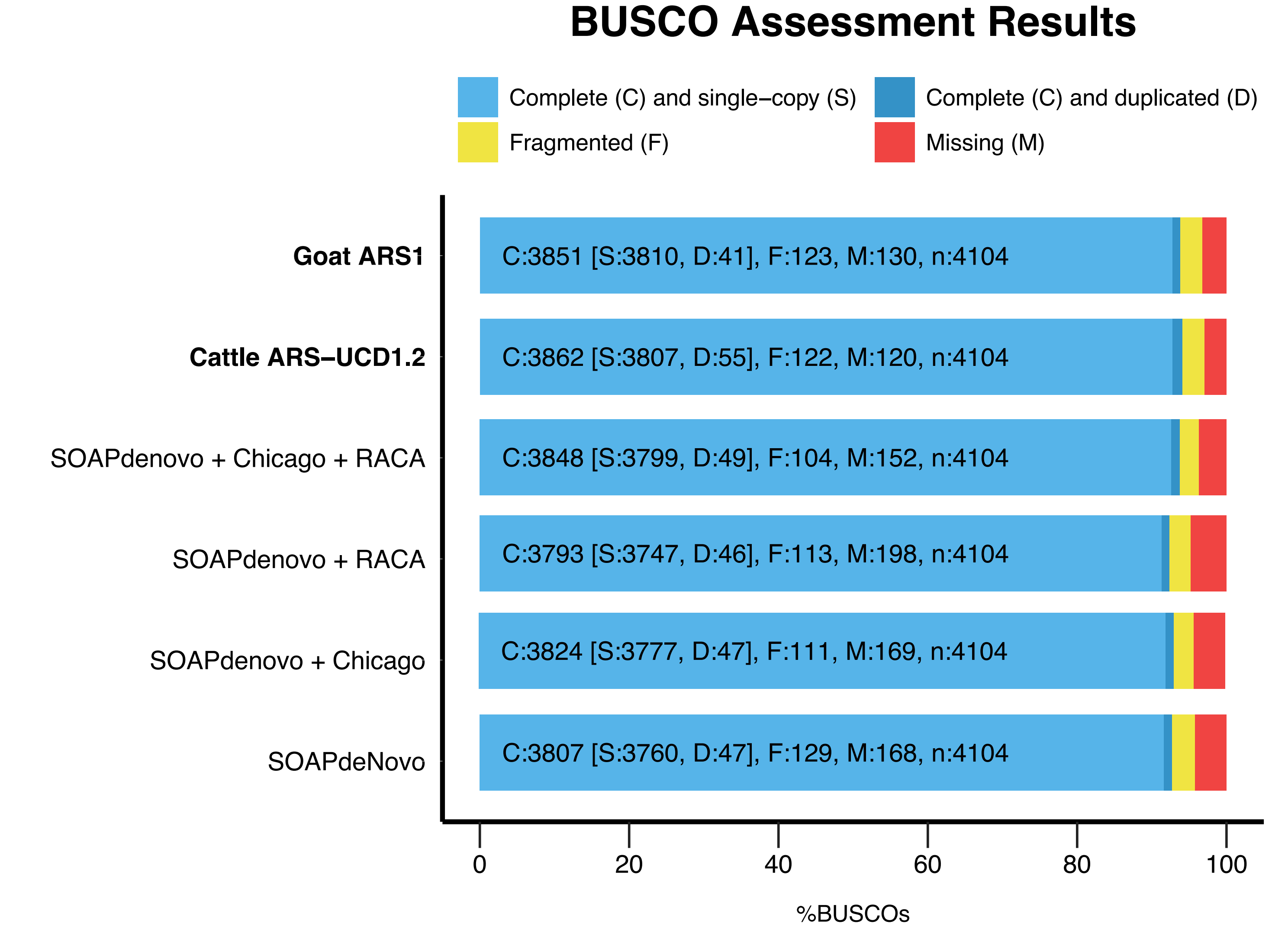

Figure4

Click here to access/download;Figure;Figure4_

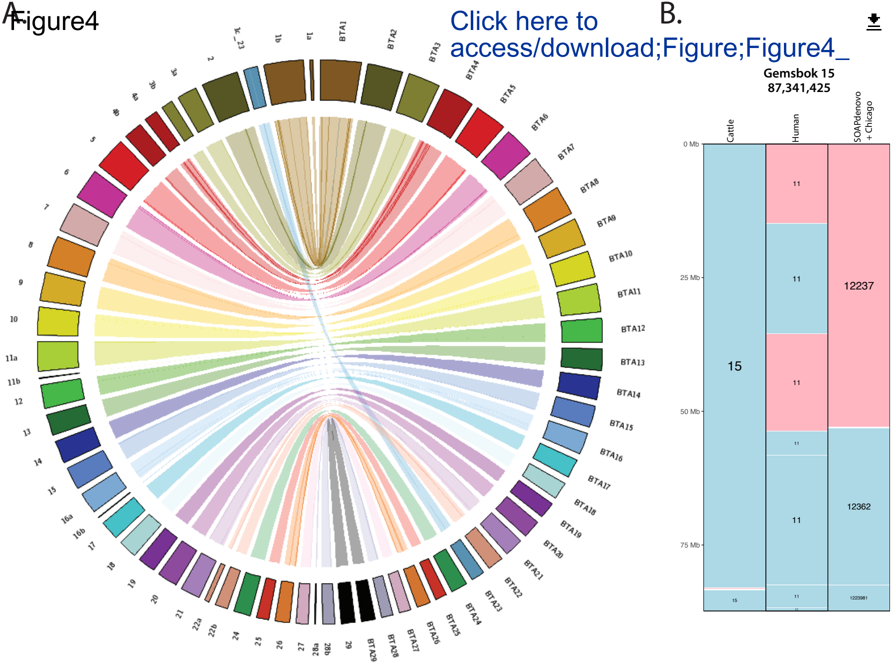

Figure5

Click here to access/download;Figure;Figure5_tree.p

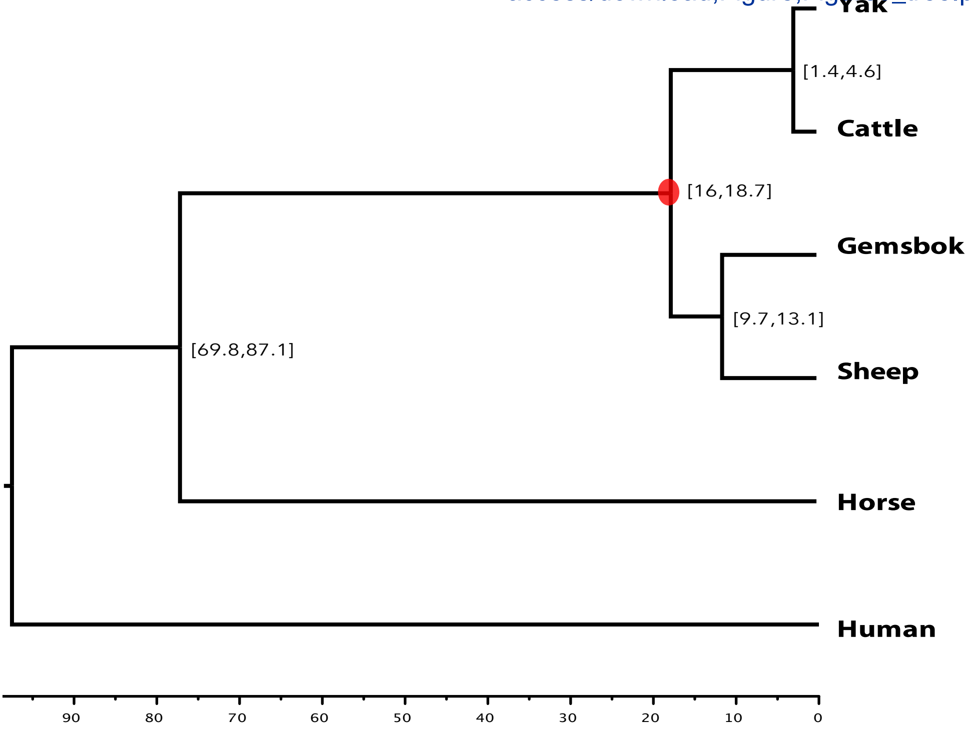

Click here to access/download
Supplementary Material
Farre_gemsbok_SupplementaryData.docx

Supplementary Figure

Click here to access/download
Supplementary Material
SupplFigure1.pdf
