# Supplementary material for: A near-chromosome-scale genome assembly of the gemsbok (Oryx gazella): an iconic antelope of the Kalahari desert
Source: Gigascience. 2019 Jan 16;8(2):giy162. doi: 10.1093/gigascience/giy162 (PMC6351727; doi:10.1093/gigascience/giy162)
Supplement: Supplemental Files [file giy162_supplemental_files.zip › Farre_gemsbok_SupplementaryData.docx]

**Supplementary Data**

**Supplementary Table 1. Summary of sequenced libraries for *Oryx gazella*.**

| **Library type** | **Inset size (bp)** | **Read length (bp)** | **Total data (Gbp)** | **Sequence coverage (fold)** | **Deposit number** |
| --- | --- | --- | --- | --- | --- |
| Illumina paired-end | 170 | 100 | 28.06 | 9.68 |  |
|  | 250 | 150 | 38.01 | 13.11 |  |
|  | 500 | 100 | 43.07 | 14.85 |  |
|  | 800 | 100 | 18.88 | 6.51 |  |
| Illumina  mate-pair | 2,000 | 49 | 23.72 | 8.18 |  |
|  | 5,000 | 49 | 17.52 | 6.04 |  |
|  | 10,000 | 49 | 6.26 | 2.16 |  |
|  | 20,000 | 49 | 4.11 | 1.42 |  |
| Illumina | Total | -- | 179.65 | 61.95 |  |
| Chicago | NA | 150 | 114.68 | 72.72 |  |

**Supplementary Table 2. Summary statics of interspersed** **repeat regions in *Oryx gazella.***

| **TE type** | **RepBase TEs** | | **TE proteins** | | **Combined TEs** | |
| --- | --- | --- | --- | --- | --- | --- |
|  | **Length (Mbp)** | **% genome** | **Length (Mbp)** | **% genome** | **Length (Mbp)** | **% genome** |
| DNA | 61,516,764 | 2.23 | 6,530,370 | 0.24 | 62,048,876 | 2.24 |
| LINE | 678,529,046 | 24.55 | 440,836,999 | 15.95 | 710,625,032 | 25.71 |
| LTR | 134,430,579 | 4.86 | 11,899,868 | 0.43 | 136,511,733 | 4.94 |
| SINE | 265,313,934 | 9.60 | - | - | 265,313,934 | 9.60 |
| Other | 2,468 | 0.00 | - | - | 2,468 | 0.00 |
| Unknown | 846,054 | 0.03 | - | - | 846,054 | 0.03 |
| Total | 1,140,638,845 | 41.27 | 459,267,237 | 16.62 | 1,175,348,097 | 42.52 |

**Supplementary Table 3. Summary statistics of function annotation for the predicted protein coding genes.**

|  |  | **Number** | **Percentage (%)** |
| --- | --- | --- | --- |
| Total number of predicted protein coding genes |  | 23,125 | 100.00 |
| Annotated | Swissprot | 19,949 | 86.27 |
|  | KEGG | 9,696 | 41.93 |
|  | InterPro | 17,112 | 74.00 |
|  | GO | 14,196 | 61.39 |
| Unannotated |  | 3,117 | 13.48 |
