# Supplementary material for: A near-chromosome-scale genome assembly of the gemsbok (Oryx gazella): an iconic antelope of the Kalahari desert
Source: Gigascience. 2019 Jan 16;8(2):giy162. doi: 10.1093/gigascience/giy162 (PMC6351727; doi:10.1093/gigascience/giy162)

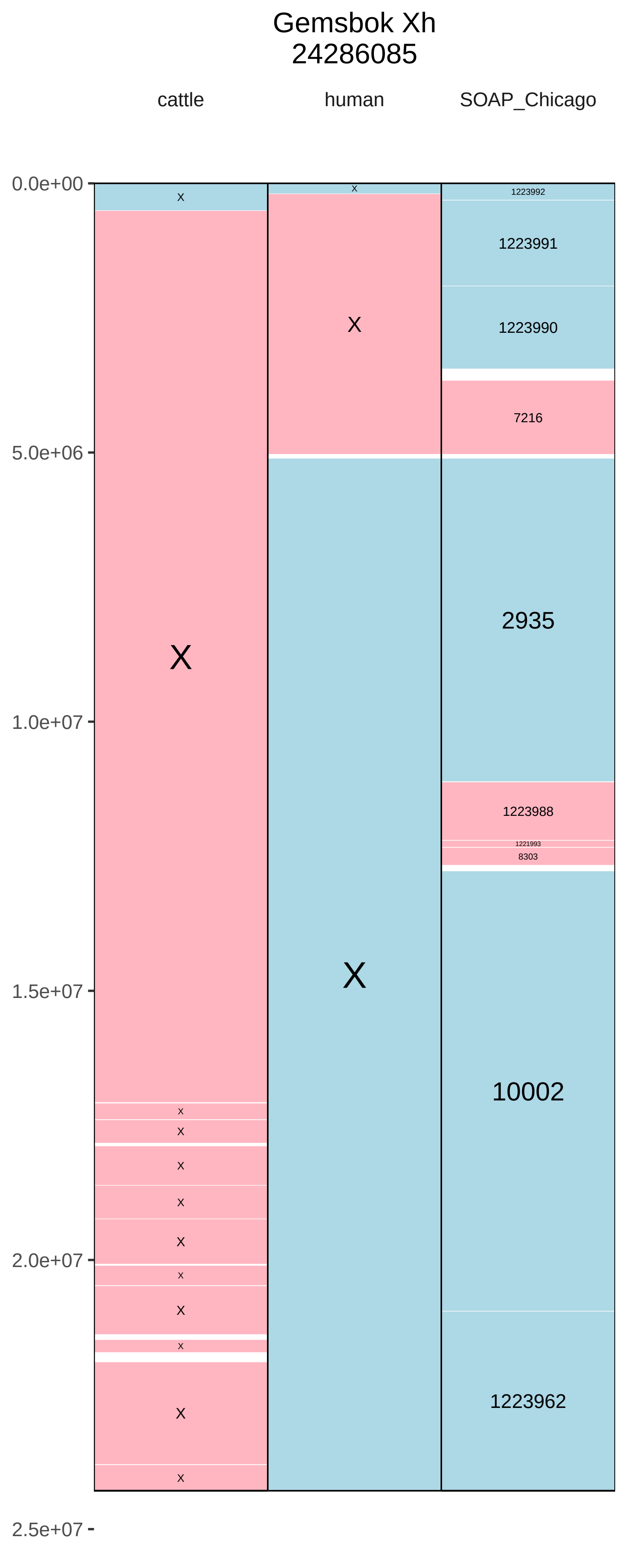
Gemsbok Xh
24286085
cattle
human
SOAP_Chicago
0.0e+00
5.0e+06
1.0e+07
1.5e+07
2.0e+07
2.5e+07
X
X
1223992
1223991
1223990
7216
2935
1223988
1221993
8303
10002
1223962

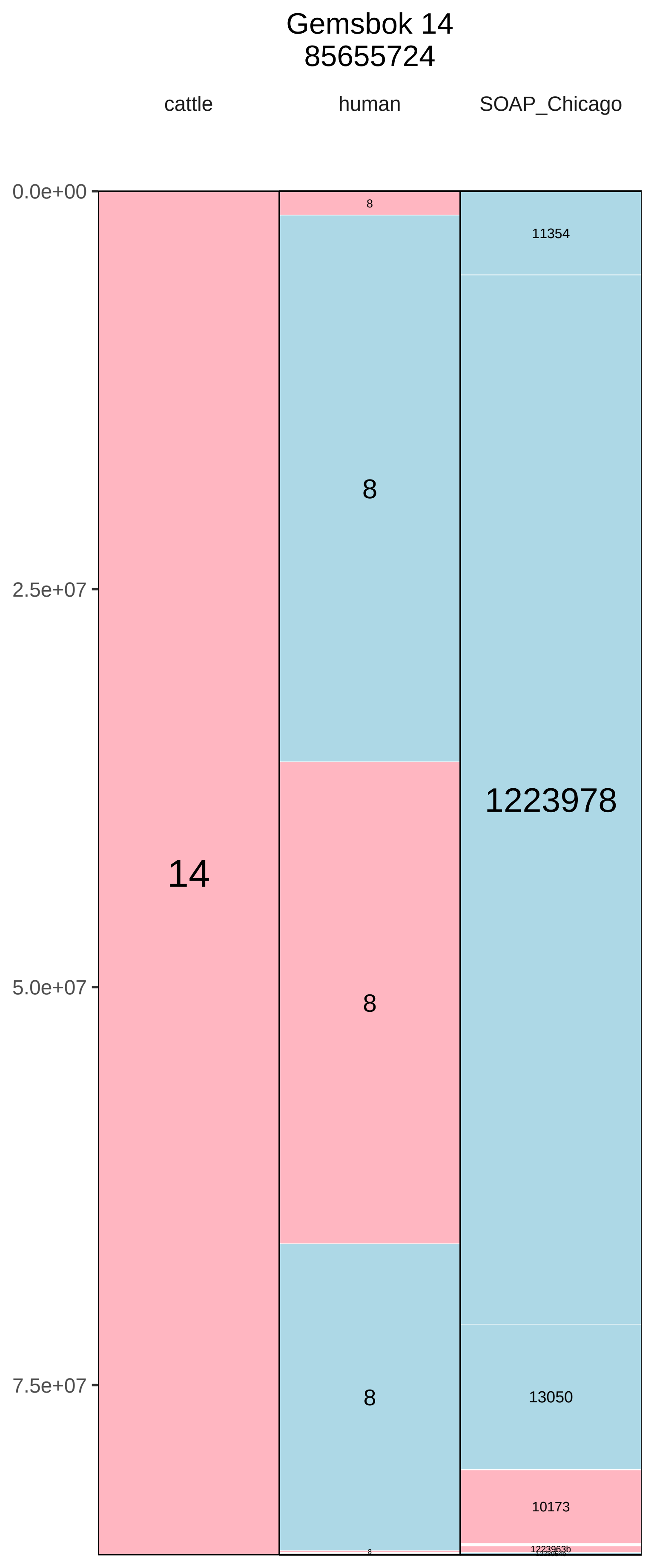
Gemsbok 14
85655724
cattle
human
SOAP_Chicago
0.0e+00
2.5e+07
5.0e+07
7.5e+07
14
8
8
8
8
11354
1223978
13050
10173

Gemsbok 16a
83386973

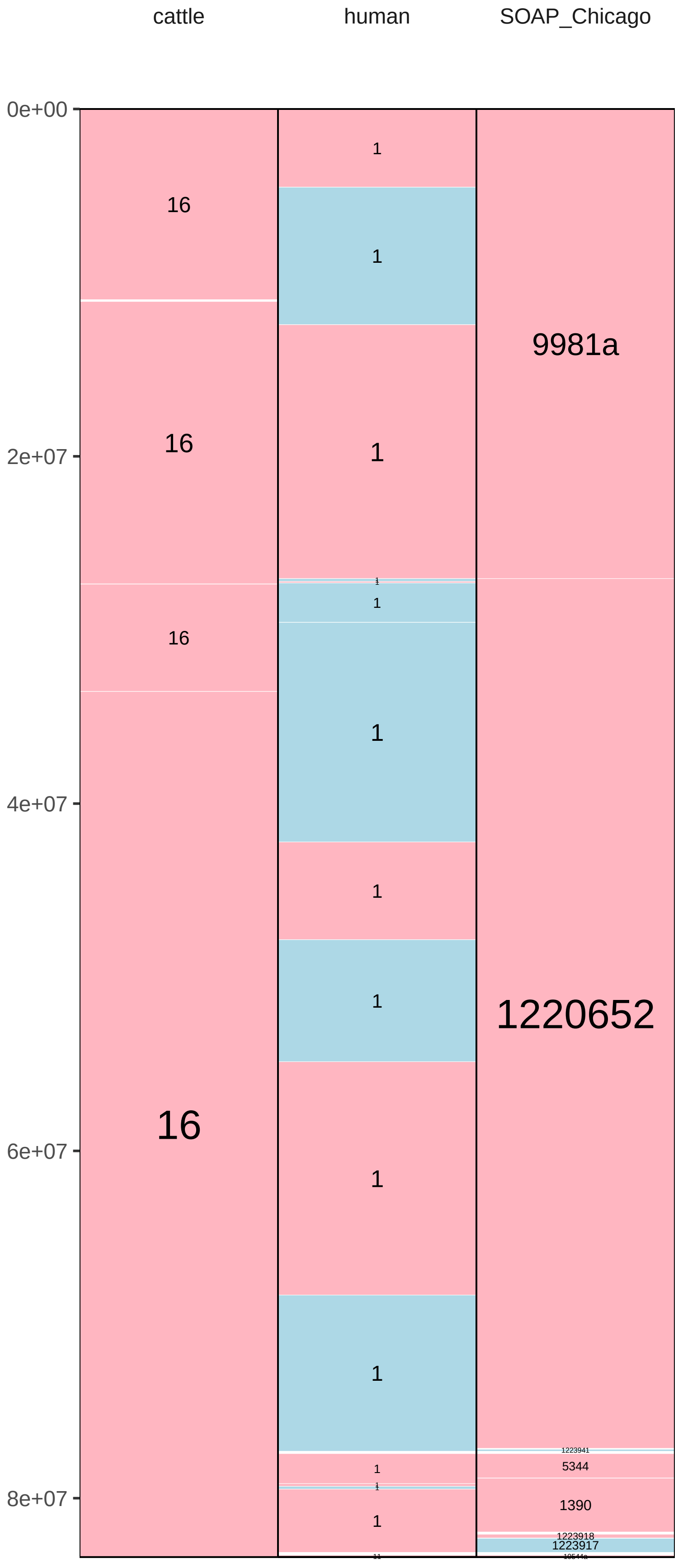

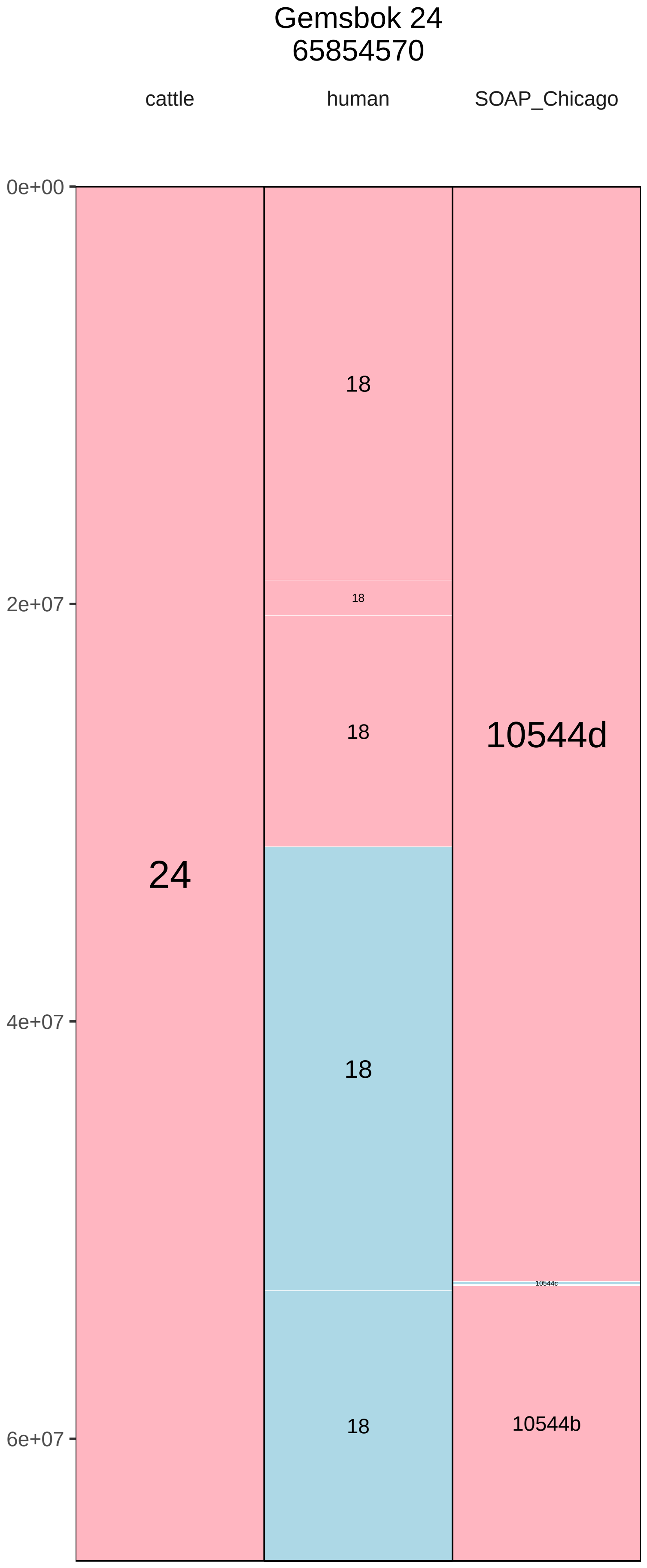

Gemsbok 24
65854570
cattle
human
SOAP_Chicago
0e+00
2e+07
4e+07
6e+07
24
18
18
18
18
18
10544d
10544c
10544b

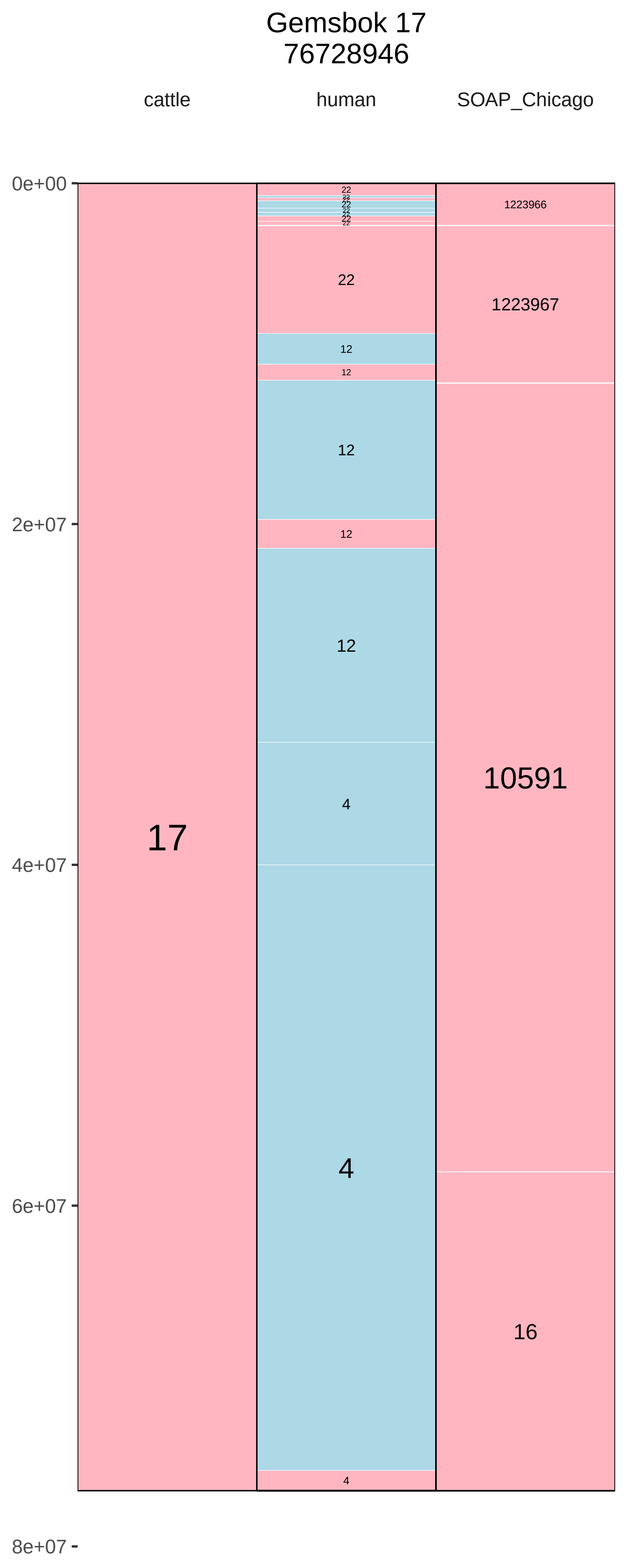
Gemsbok 17
76728946
cattle
human
SOAP_Chicago
0e+00
2e+07
4e+07
6e+07
8e+07
17
22
12
12
12
12
12
4
4
4
1223966
1223967
10591
16

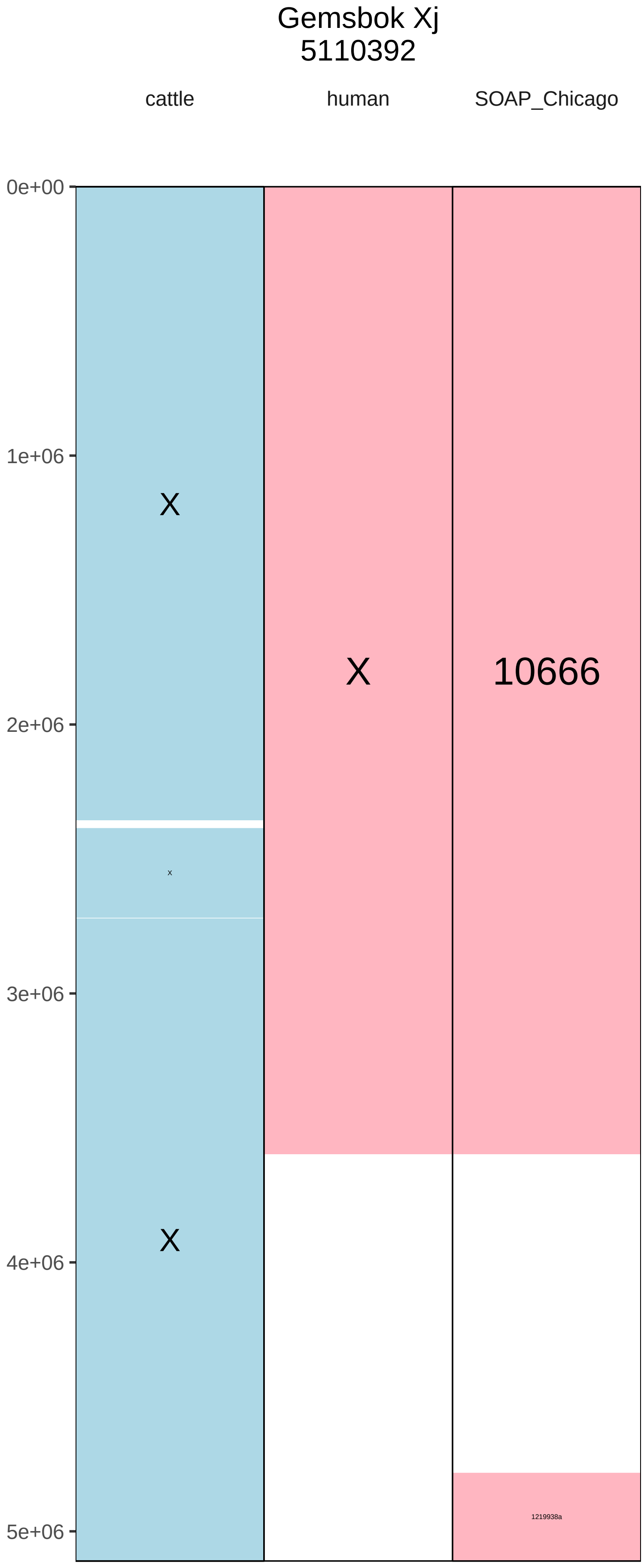
Gemsbok Xj
5110392
cattle
human
SOAP_Chicago
0e+00
1e+06
2e+06
3e+06
4e+06
5e+06
X
x
X
X
10666
1219938a

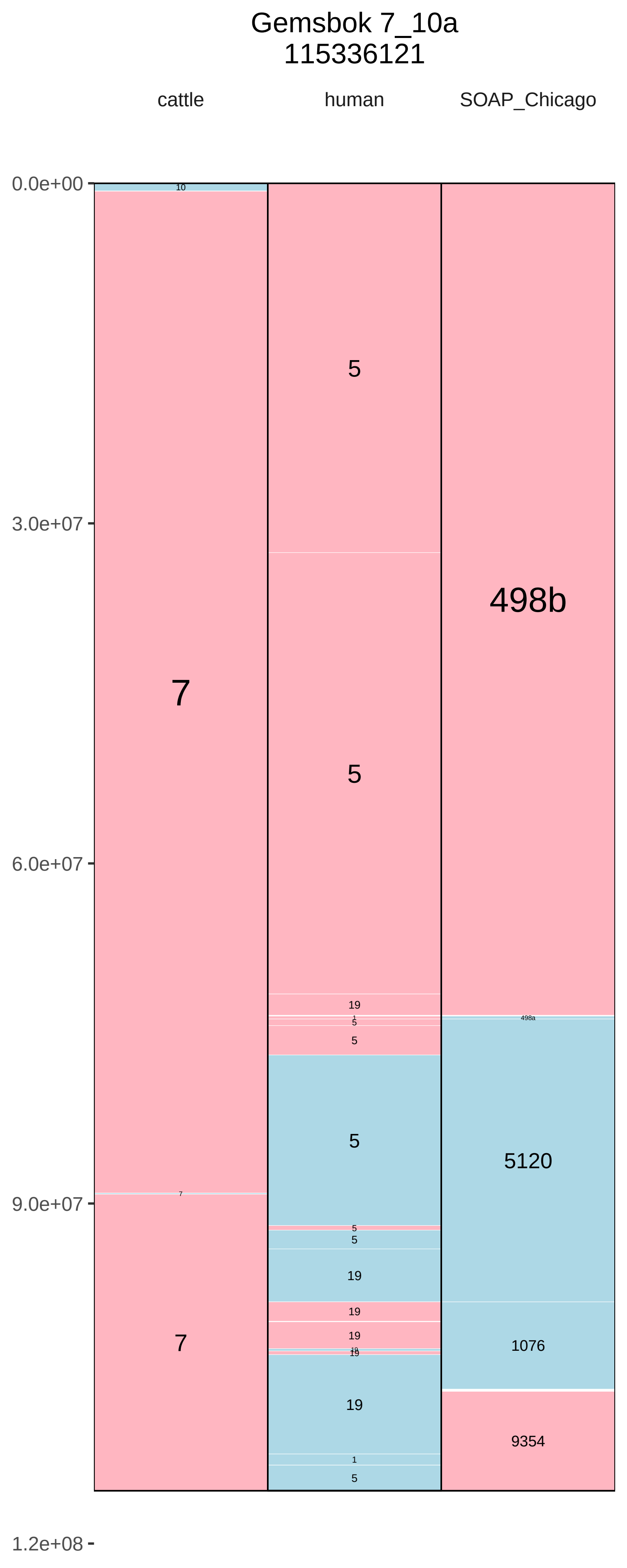
Gemsbok 7_10a
115336121
cattle
human
SOAP_Chicago
0.0e+00
3.0e+07
6.0e+07
9.0e+07
1.2e+08
10
7
7
7
5
5
19
1
5
5
5
5
5
19
19
19
19
19
1
5
498b
498a
5120
1076
9354

Gemsbok 10b
107983847

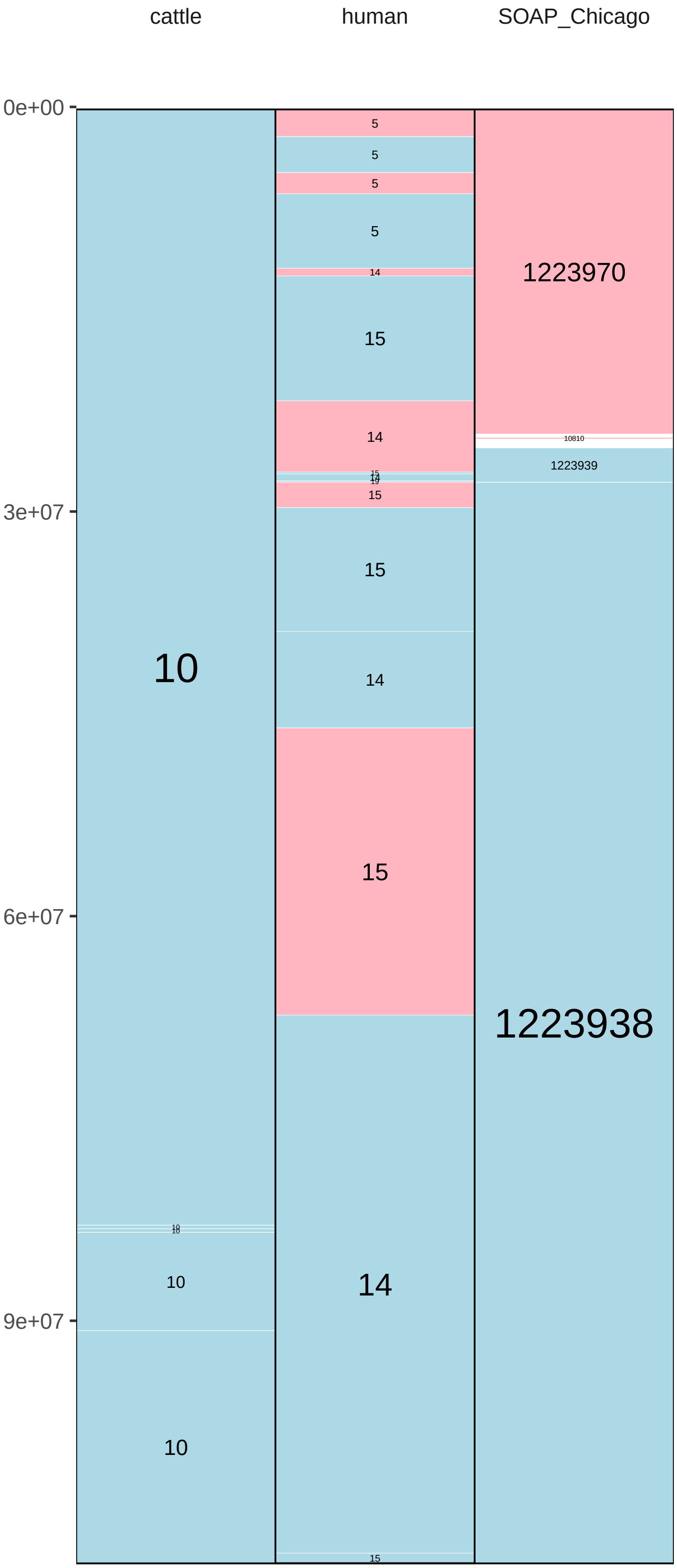

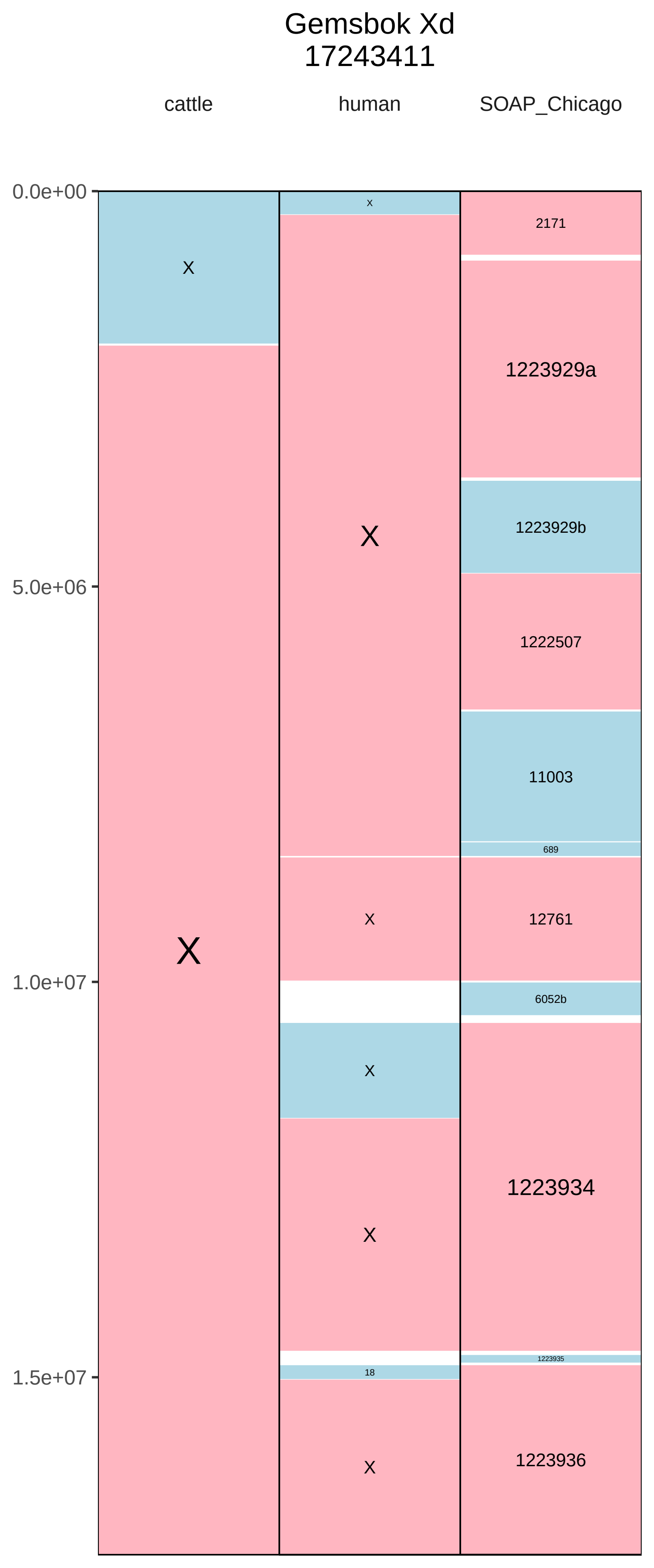
Gemsbok Xd
17243411
cattle
human
SOAP_Chicago
0.0e+00
5.0e+06
1.0e+07
1.5e+07
X
X
X
X
X
X
18
X
X
2171
1223929a
1223929b
1222507
11003
689
12761
6052b
1223934
1223935
1223936

Gemsbok 8
117223004

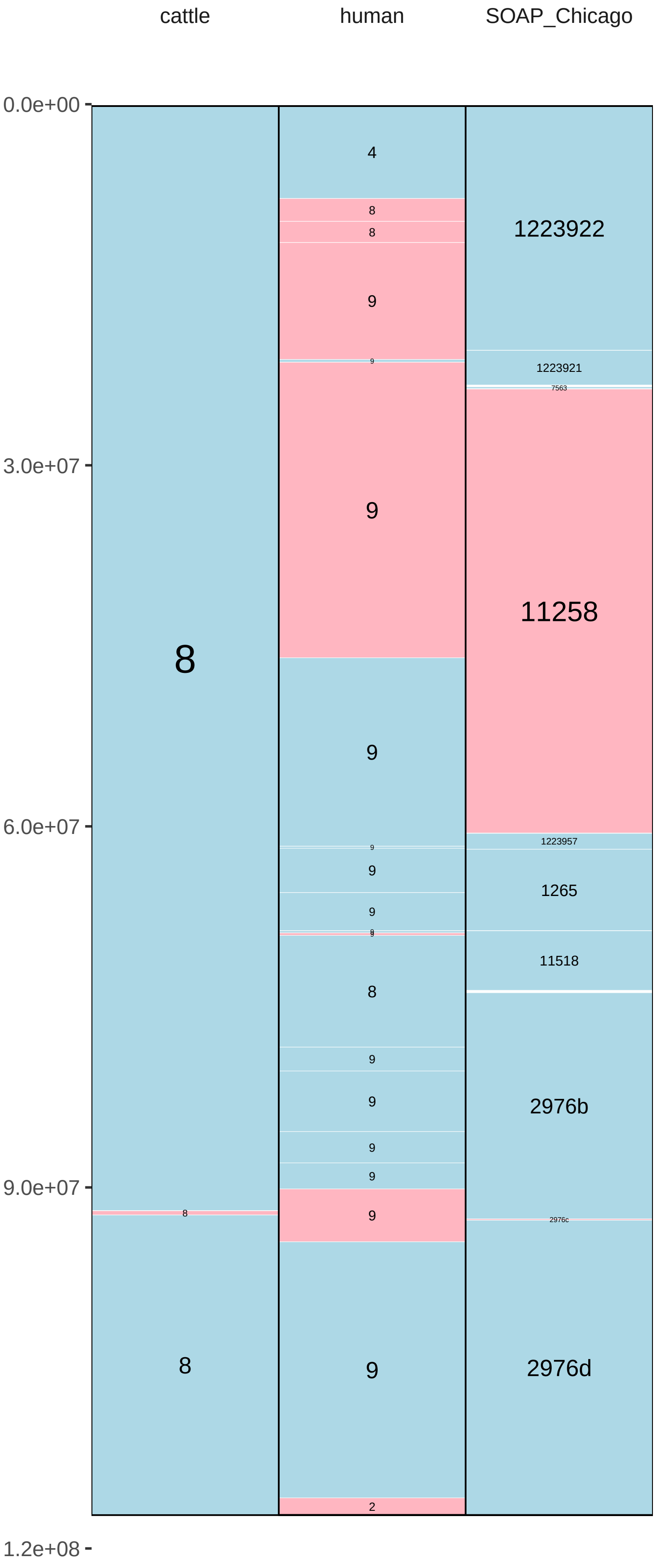

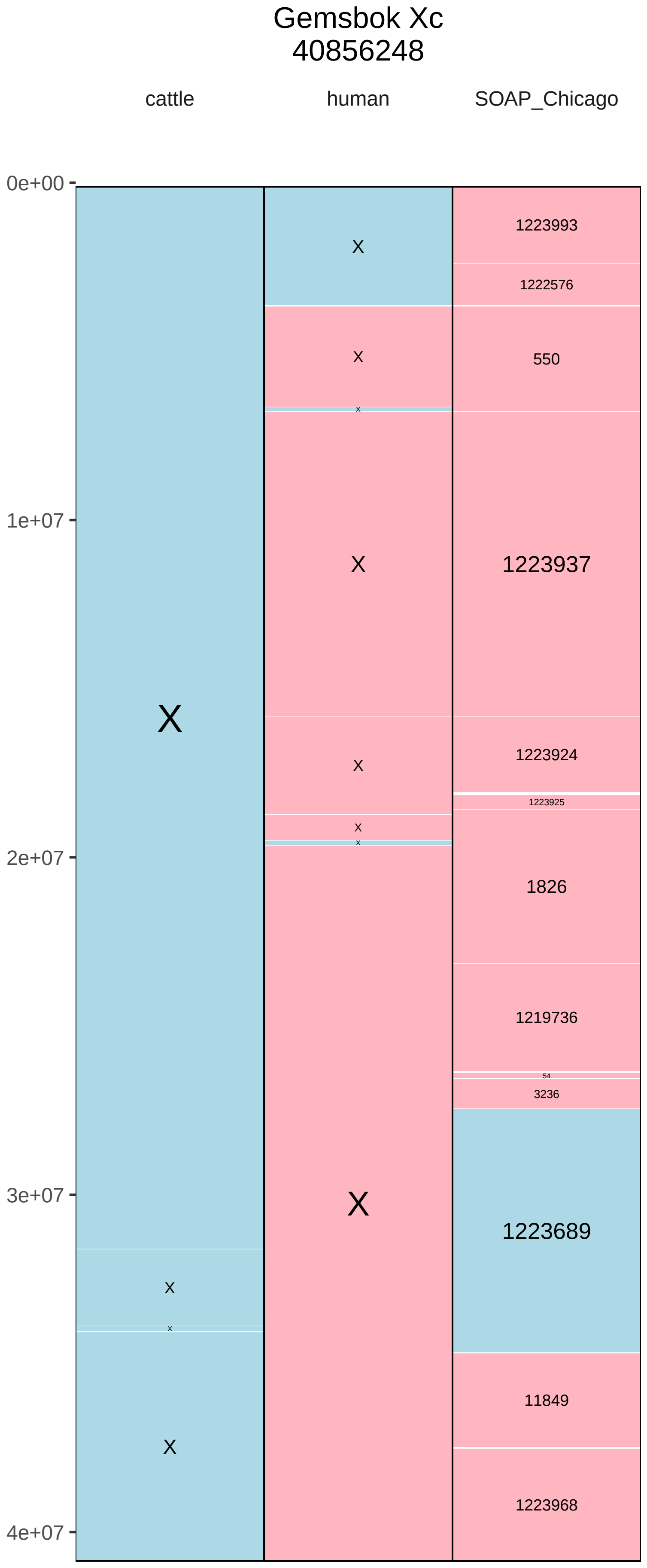

Gemsbok Xc
40856248
cattle
human
SOAP_Chicago
0e+00
1e+07
2e+07
3e+07
4e+07
X
X
X
X
X
X
X
X
X
1223993
1222576
550
1223937
1223924
1223925
1826
1219736
54
3236
1223689
11849
1223968

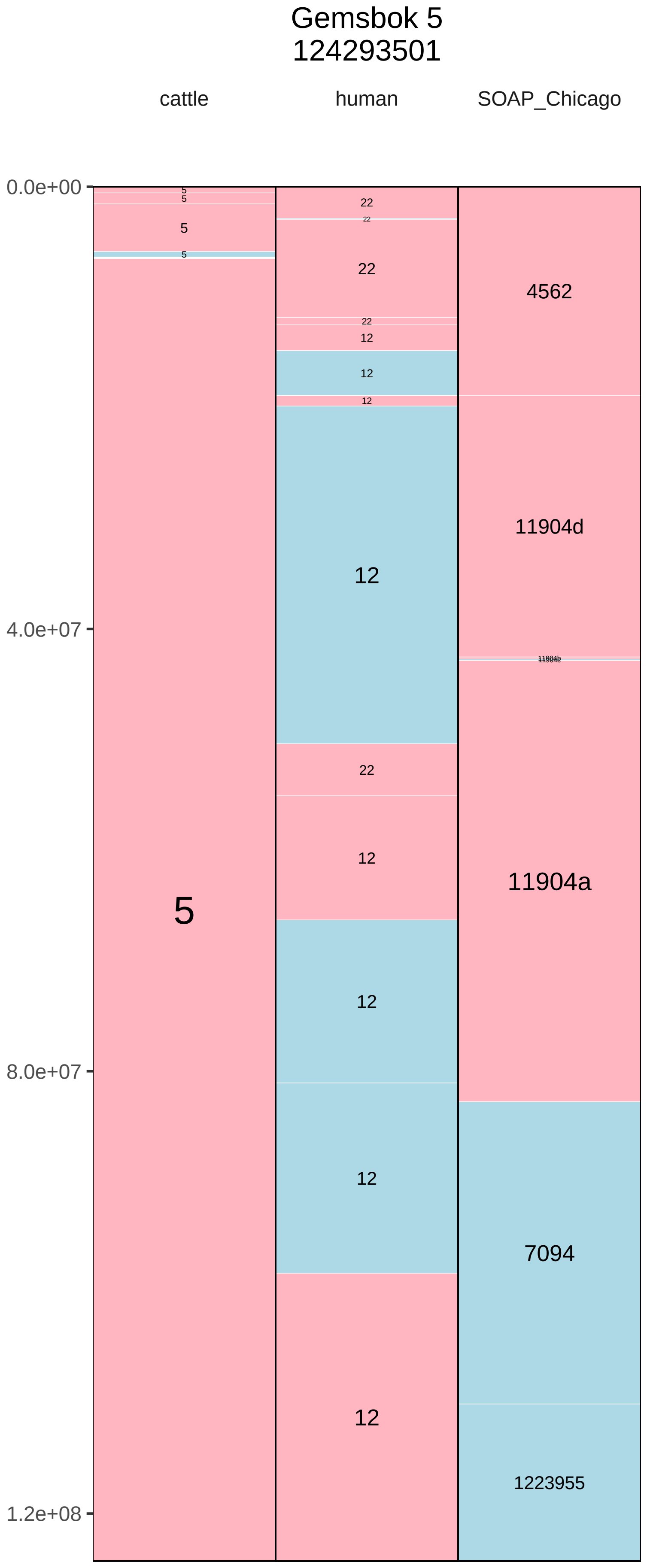
Gemsbok 5
124293501
cattle
human
SOAP_Chicago
0.0e+00
4.0e+07
8.0e+07
1.2e+08
5
5
5
5
5
22
22
22
22
12
12
12
12
22
12
12
12
12
4562
11904d
11904a
7094
1223955

Gemsbok 19
67895924

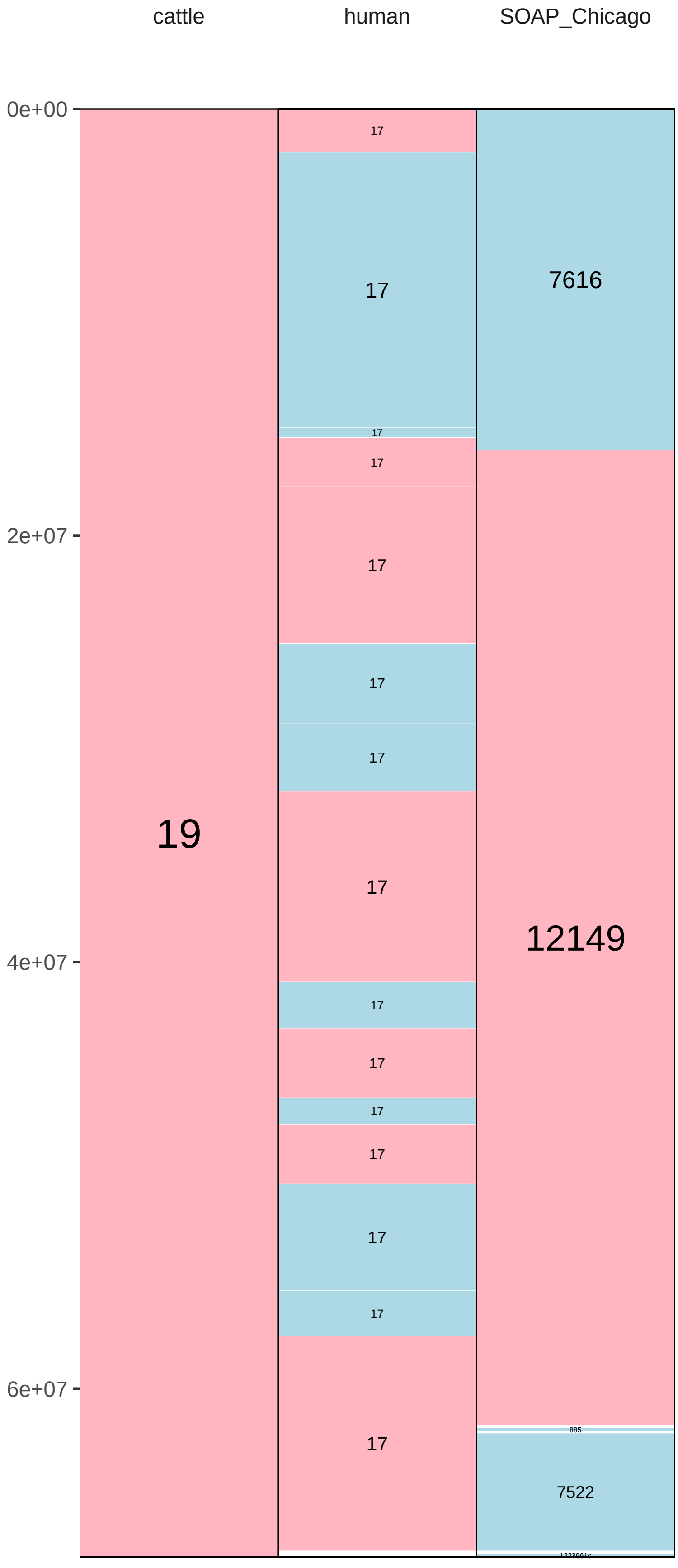

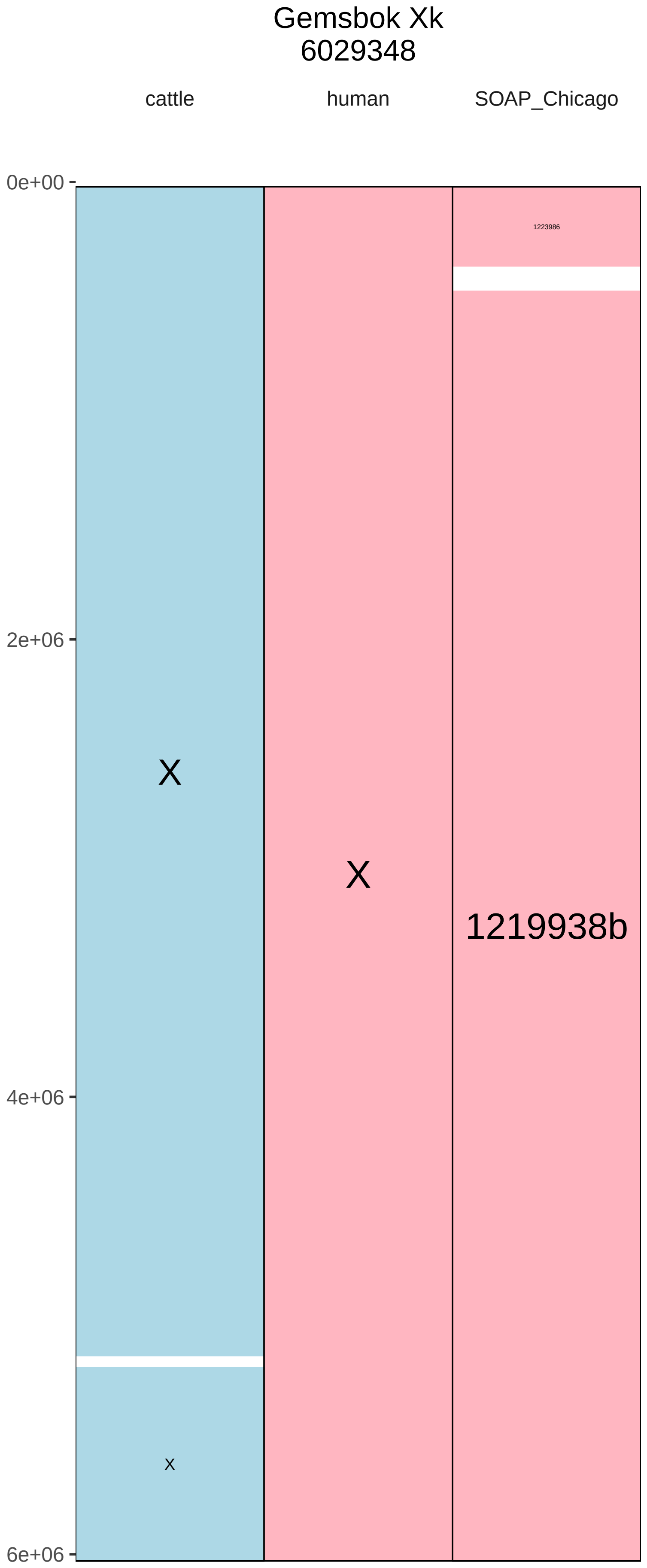
Gemsbok Xk
6029348
cattle
human
SOAP_Chicago
0e+00
2e+06
4e+06
6e+06
1223986
X
X
1219938b
X

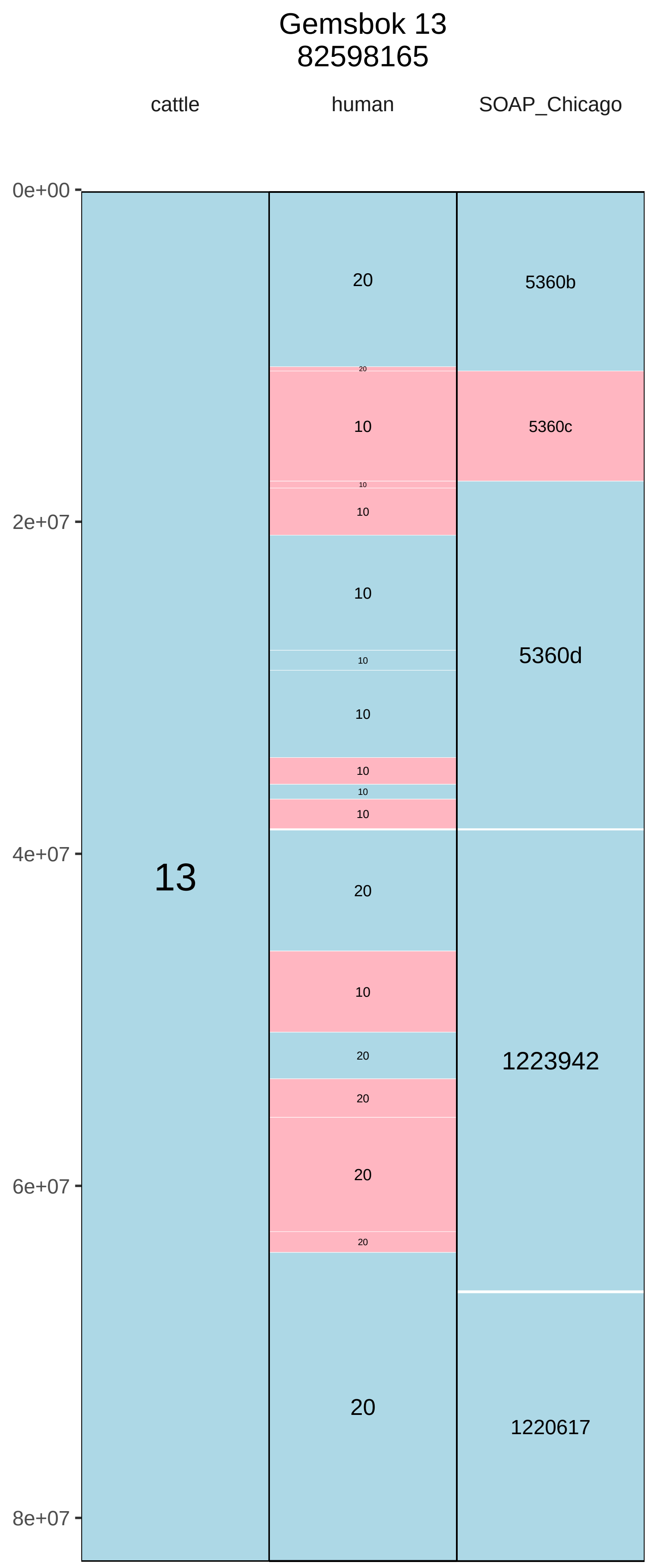
Gemsbok 13
82598165
cattle
human
SOAP_Chicago
0e+00
2e+07
4e+07
6e+07
8e+07
13
20
20
10
10
10
10
10
10
10
10
10
20
10
20
20
20
20
20
5360b
5360c
5360d
1223942
1220617

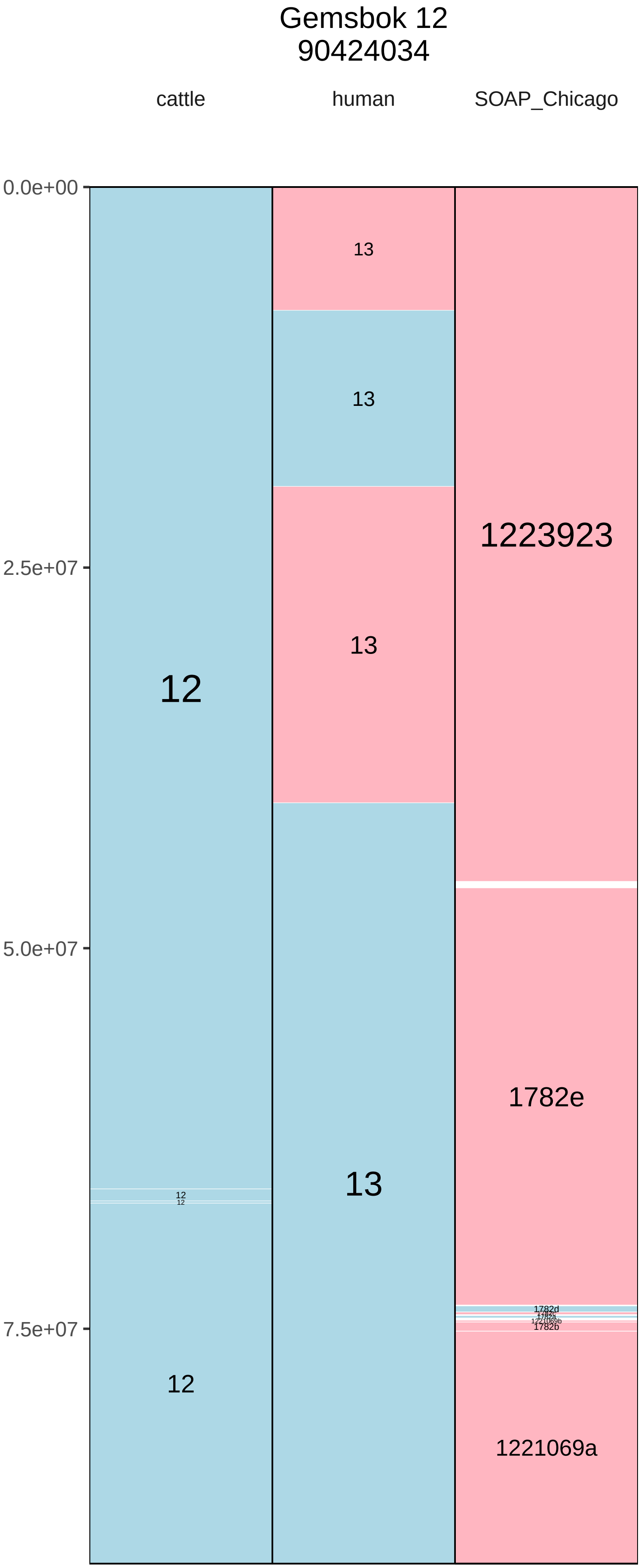
Gemsbok 12
90424034
cattle
human
SOAP_Chicago
0.0e+00
2.5e+07
5.0e+07
7.5e+07
12
12
12
12
13
13
13
13
1223923
1782e
1782d
1782c
1782a
1221069b
1782b
1221069a

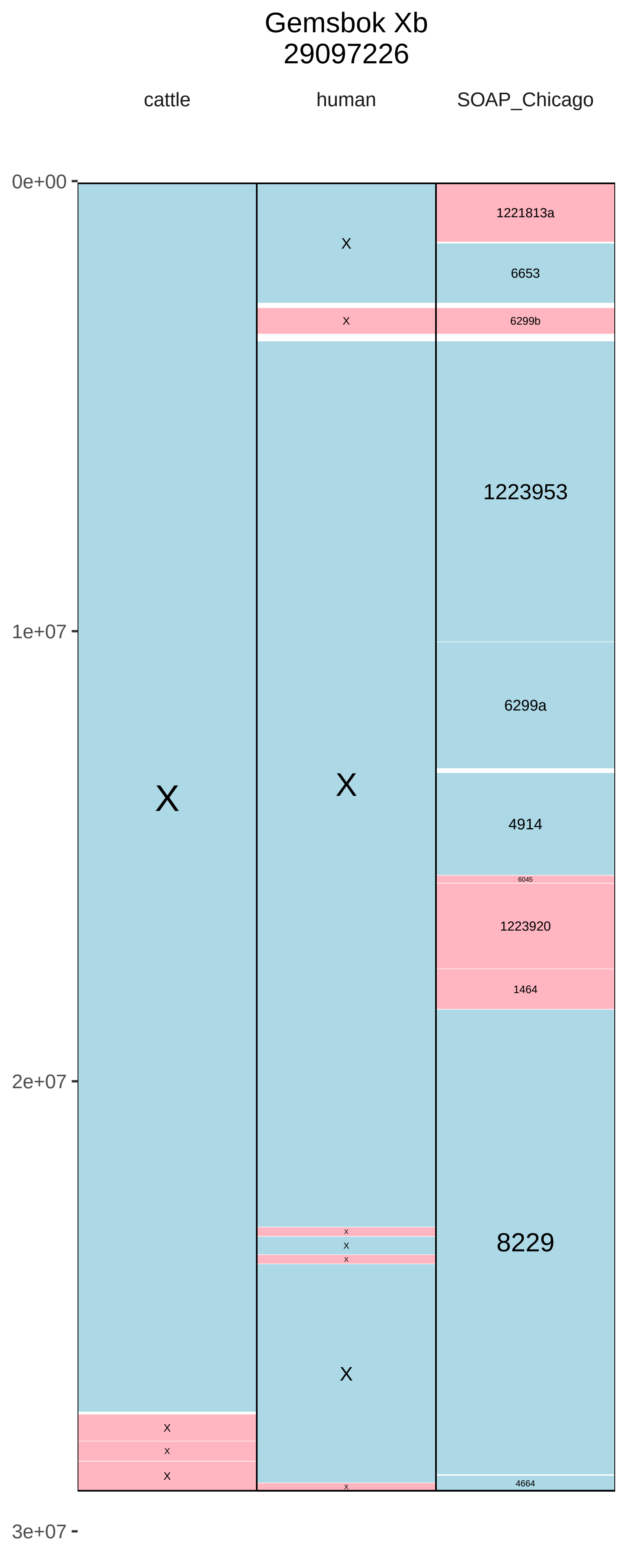
Gemsbok Xb
29097226
cattle
human
SOAP_Chicago
0e+00
1e+07
2e+07
3e+07
X
x
x
x
X
X
X
x
x
x
X
1221813a
6653
6299b
1223953
6299a
4914
6045
1223920
1464
8229
4664

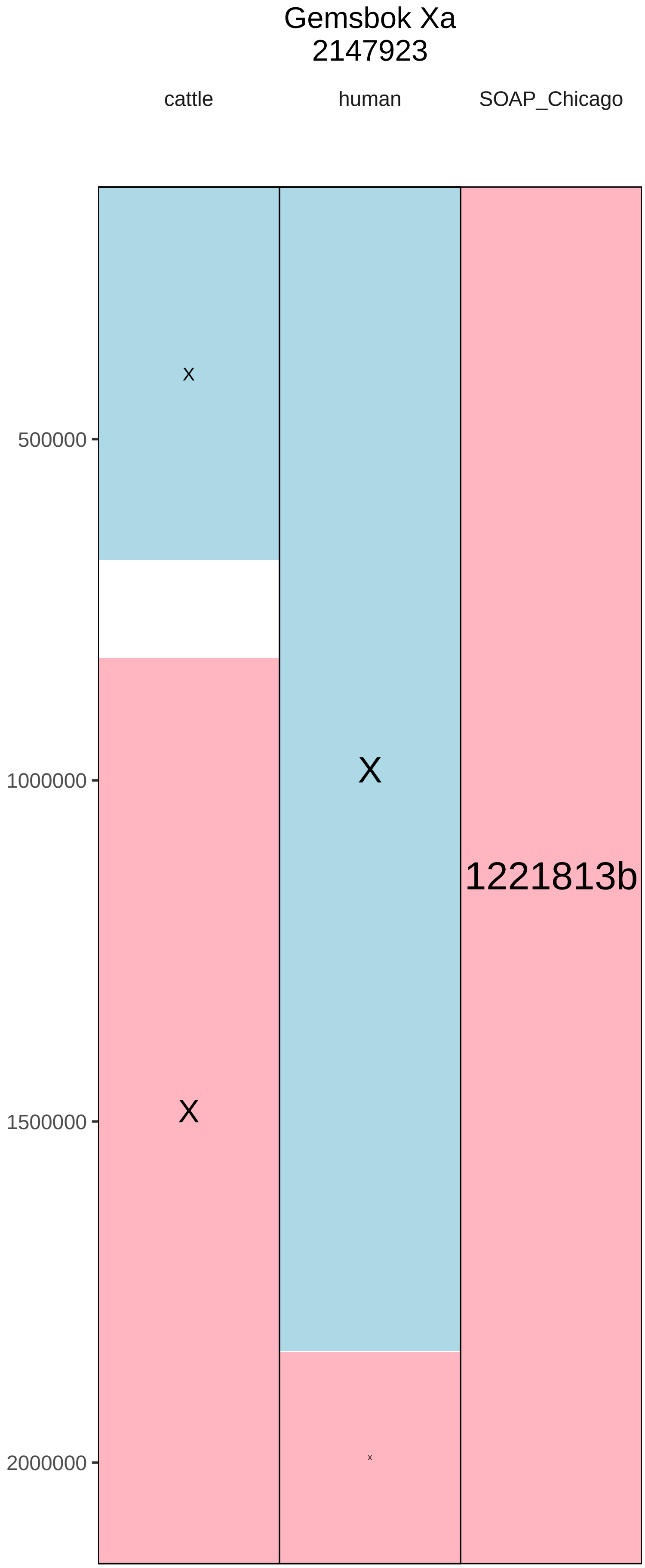

Gemsbok Xa
2147923
cattle
human
SOAP_Chicago
X
500000
X
1000000
1221813b
X
1500000
2000000

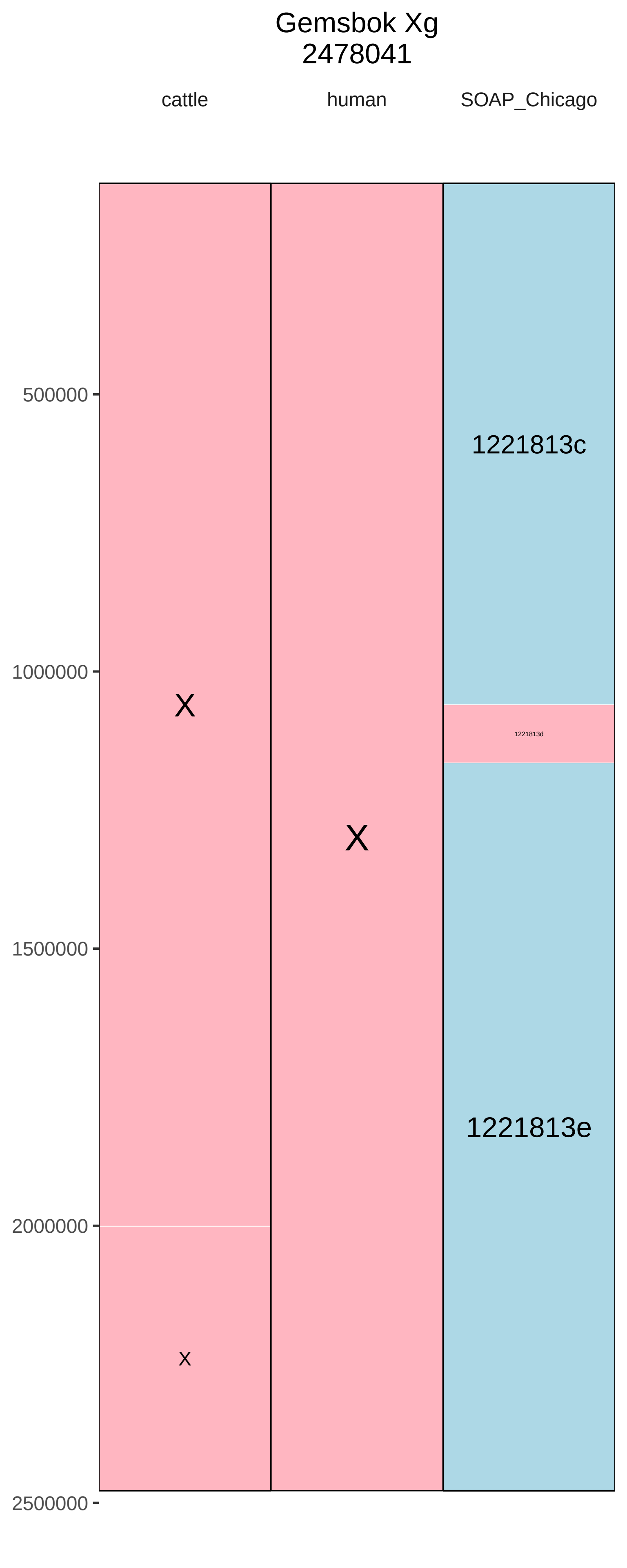
Gemsbok Xg
2478041
cattle
human
SOAP_Chicago
500000
1000000
1500000
2000000
2500000
X
X
X
1221813c
1221813d
1221813e

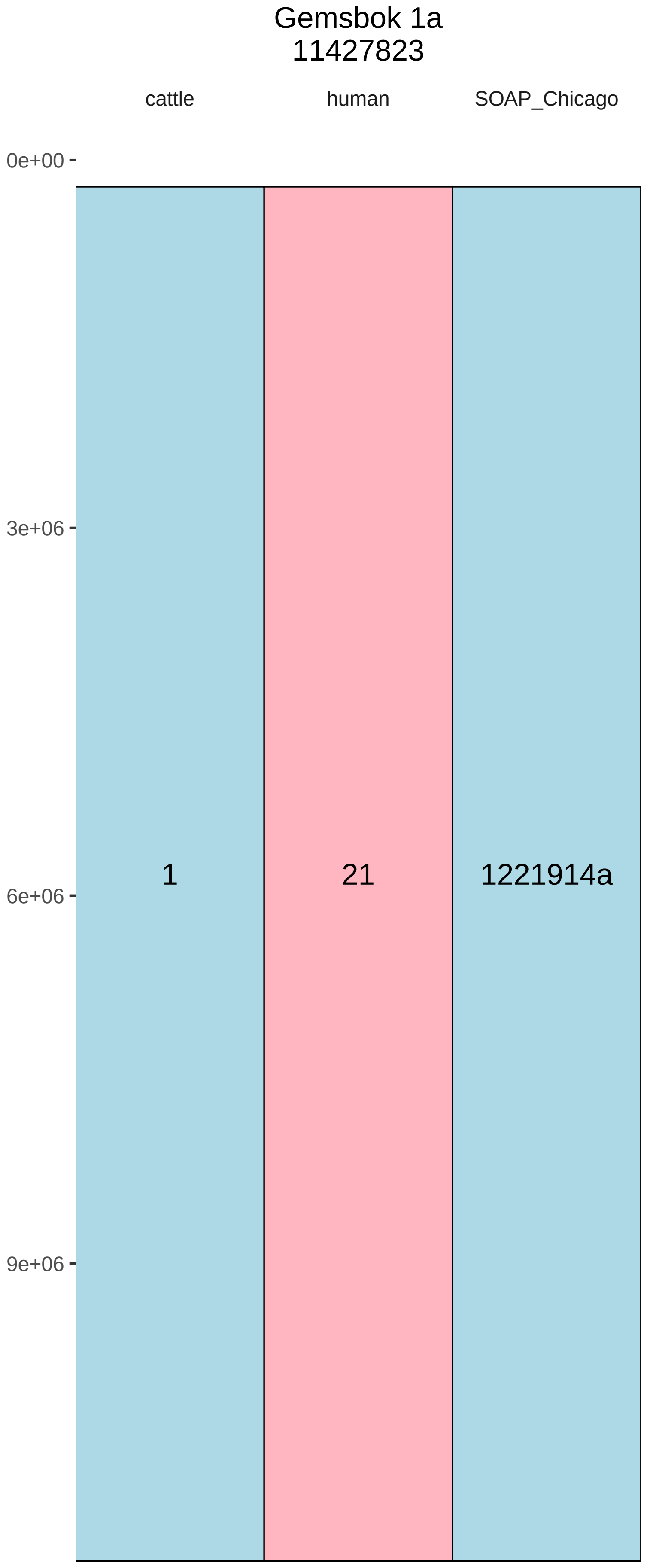
Gemsbok 1a
11427823
cattle
human
SOAP_Chicago
0e+00
3e+06
6e+06
9e+06
1
21
1221914a

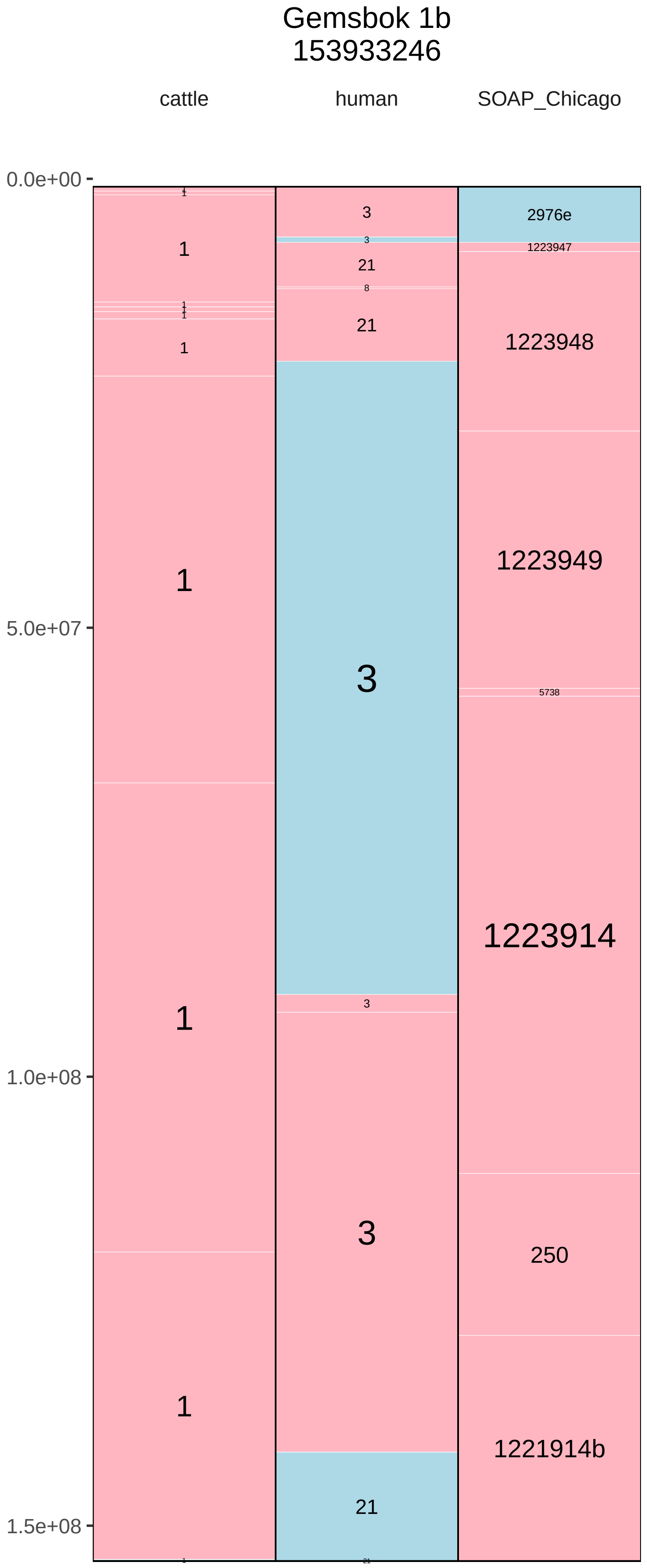
Gemsbok 1b
153933246
cattle
human
SOAP_Chicago
0.0e+00
5.0e+07
1.0e+08
1.5e+08
1
1
1
1
1
3
21
21
3
3
3
21
2976e
1223947
1223948
1223949
5738
1223914
250
1221914b

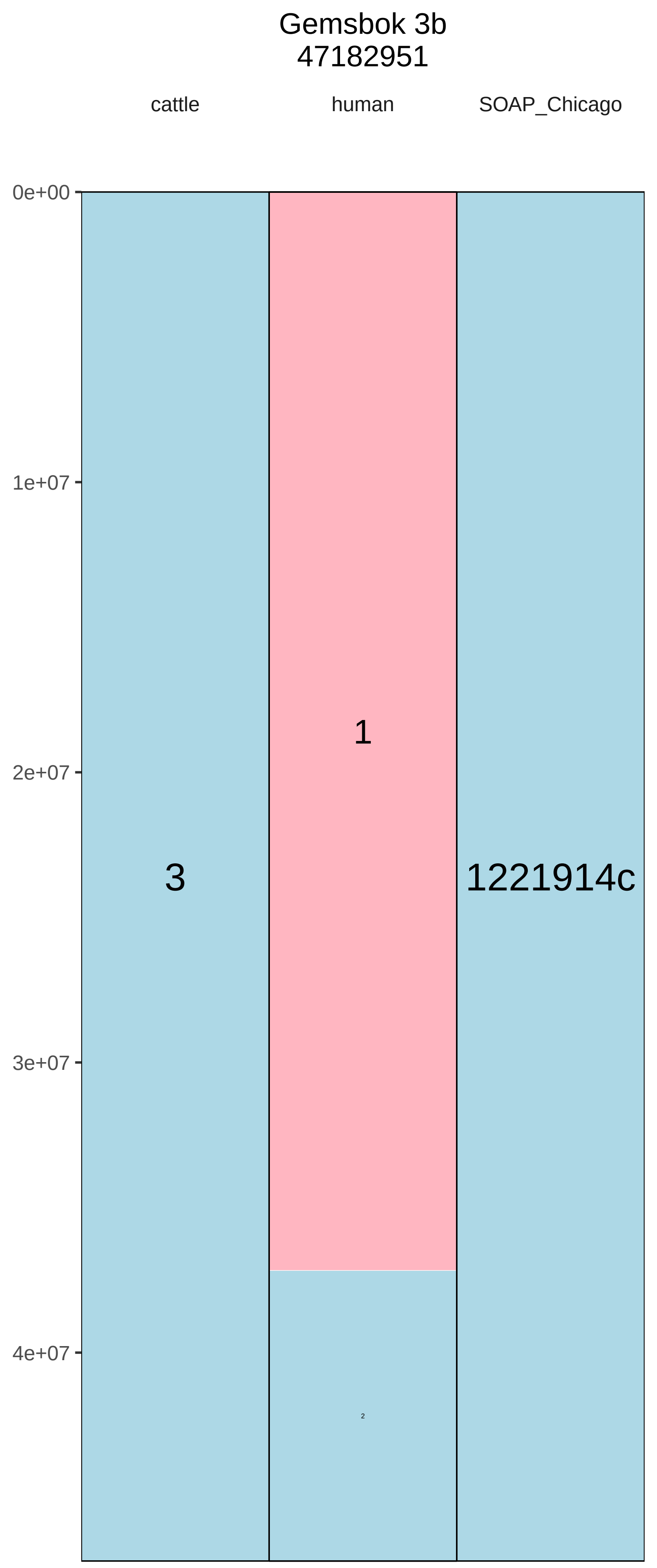
Gemsbok 3b
47182951
cattle
human
SOAP_Chicago
0e+00
1e+07
2e+07
3e+07
4e+07
3
1
2
1221914c

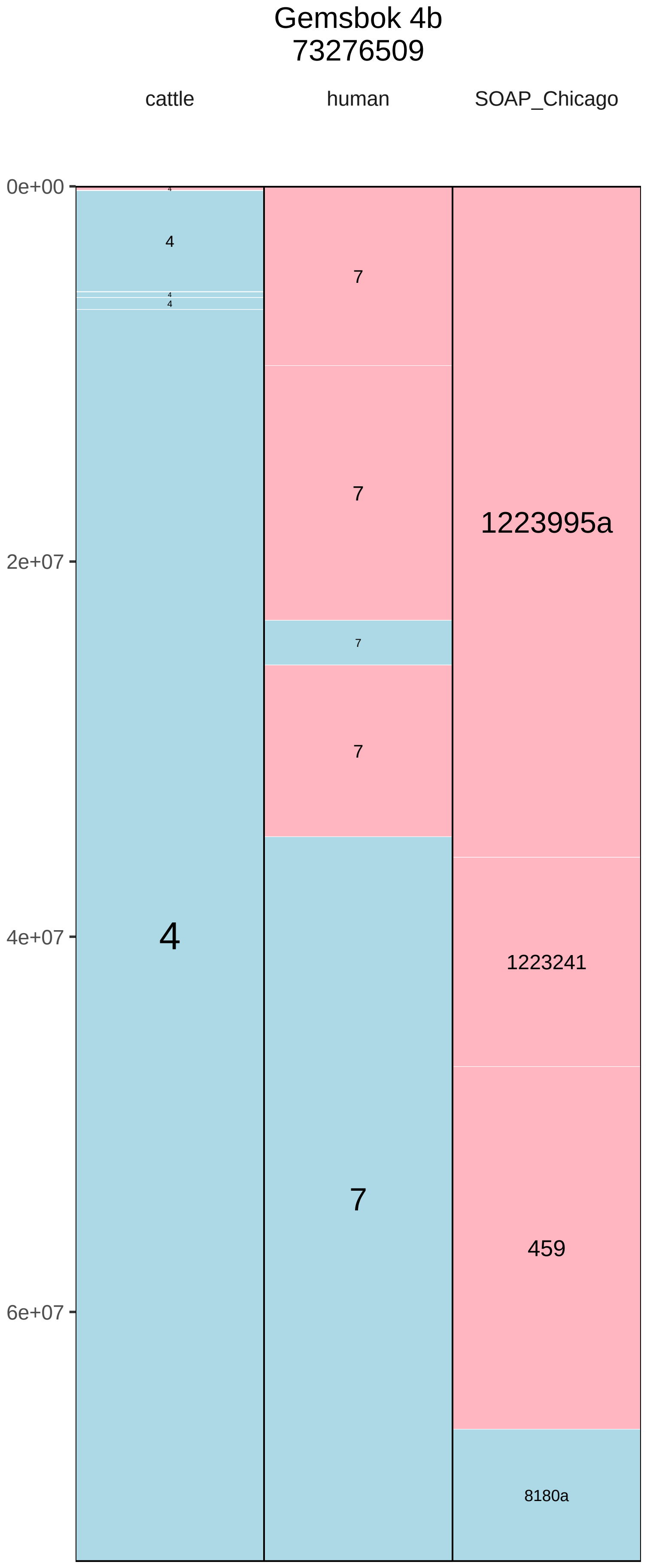

Gemsbok 4b
73276509
cattle
human
SOAP_Chicago
0e+00
2e+07
4e+07
6e+07
4
4
7
7
7
7
7
1223995a
1223241
459
8180a

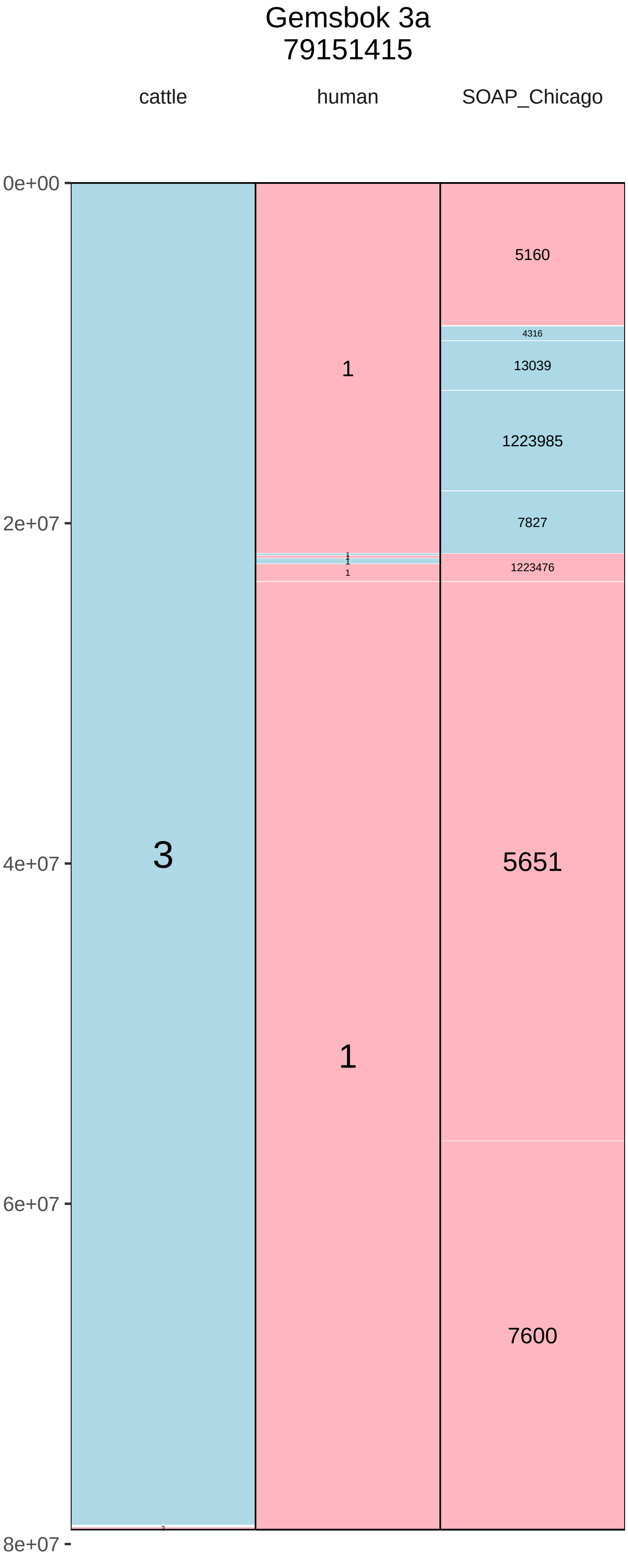
Gemsbok 3a
79151415
cattle
human
SOAP_Chicago
0e+00
2e+07
4e+07
6e+07
8e+07
3
1
1
1
5160
4316
13039
1223985
7827
1223476
5651
7600

Gemsbok 15
87341425

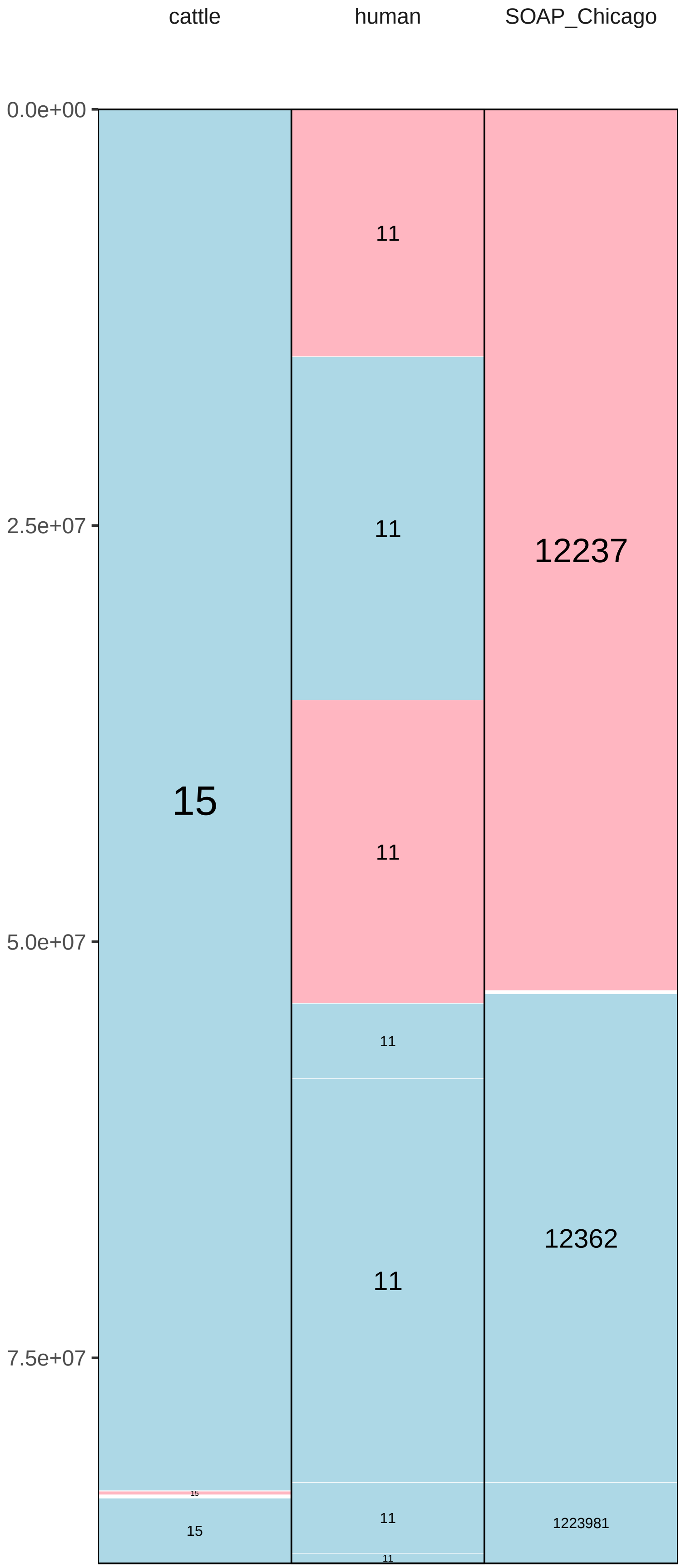

Gemsbok 18
69623073

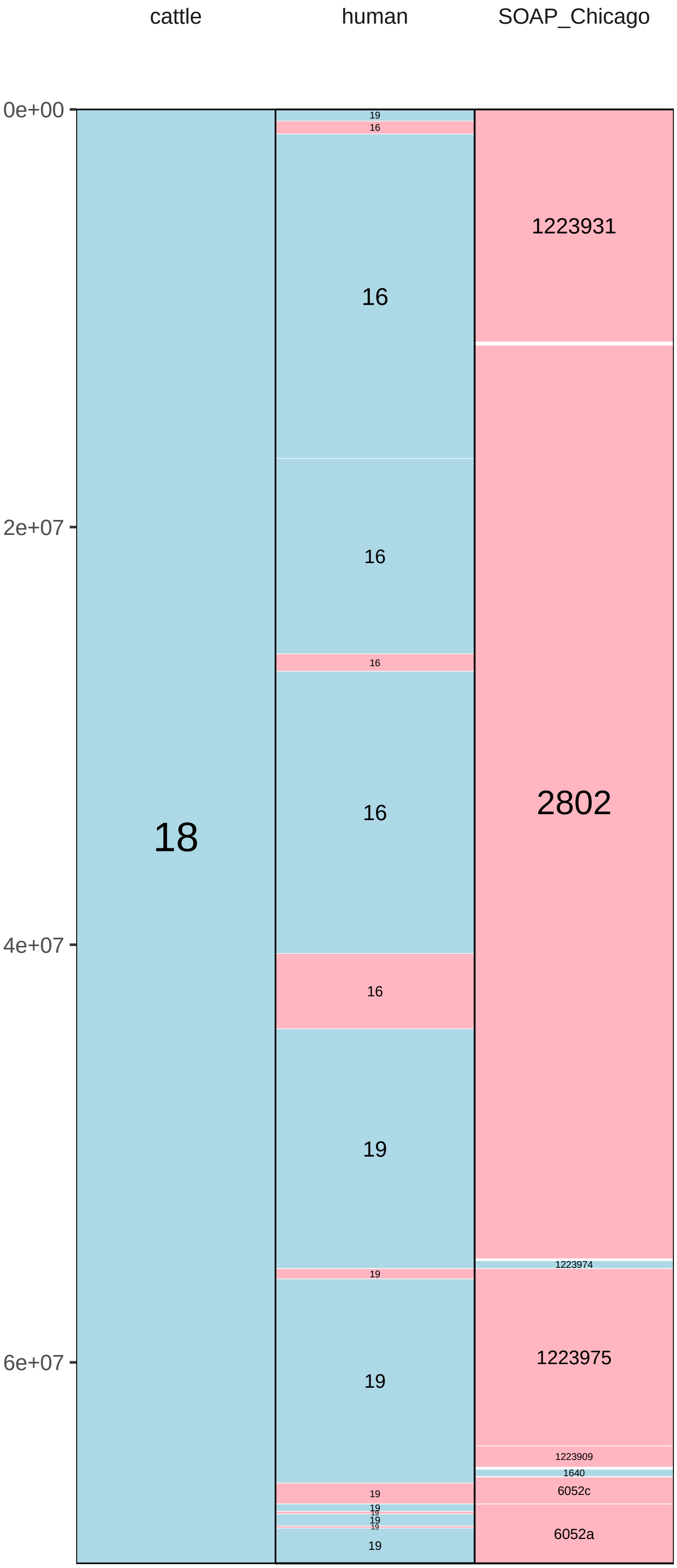

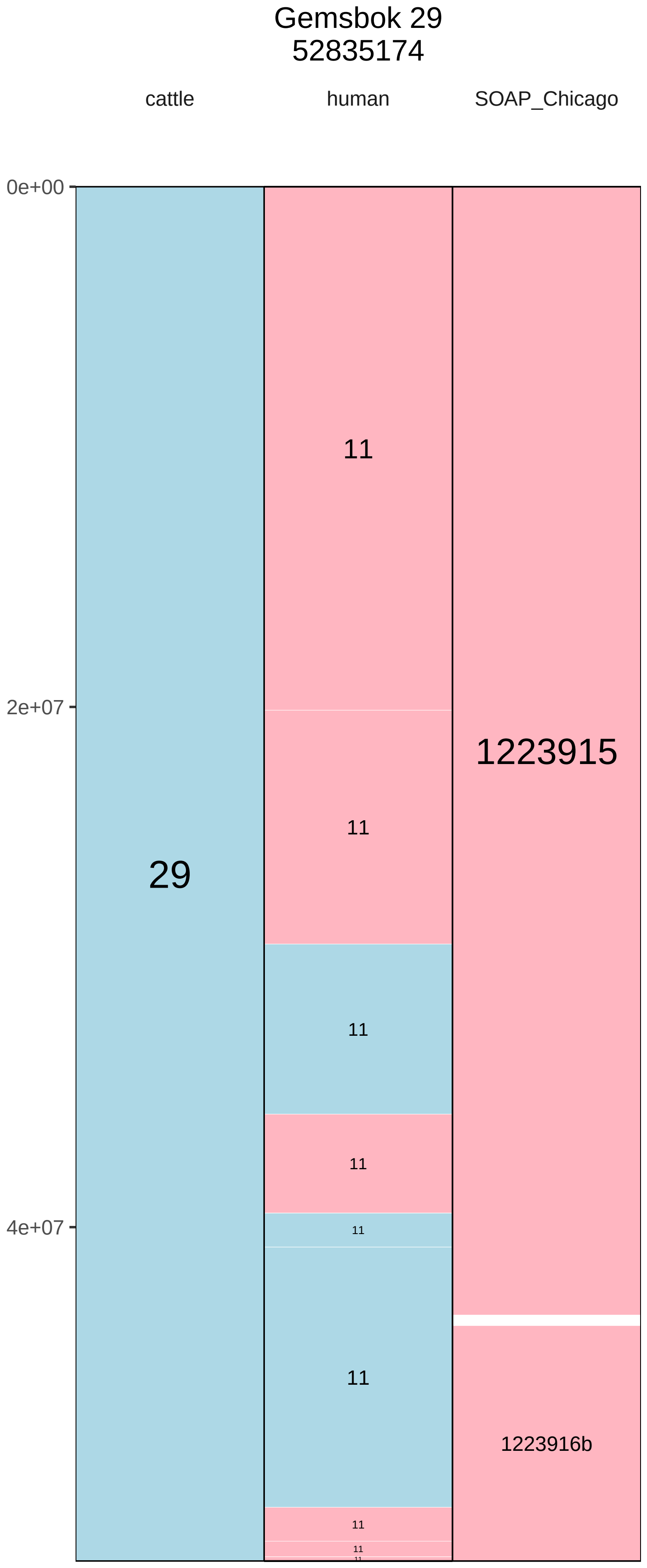
Gemsbok 29
52835174
cattle
human
SOAP_Chicago
0e+00
2e+07
4e+07
29
11
11
11
11
11
11
11
11
1223915
1223916b

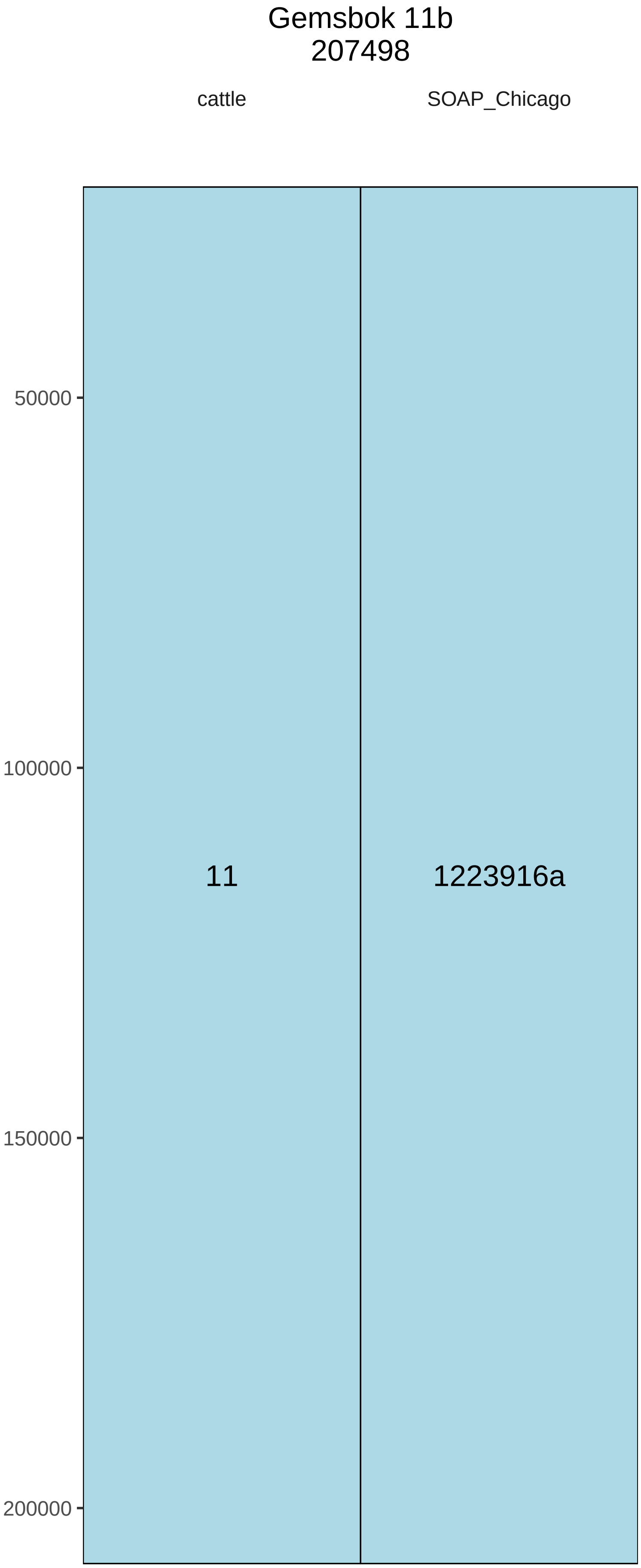

Gemsbok 11b
207498
cattle
SOAP_Chicago
50000
100000
150000
200000
11
1223916a

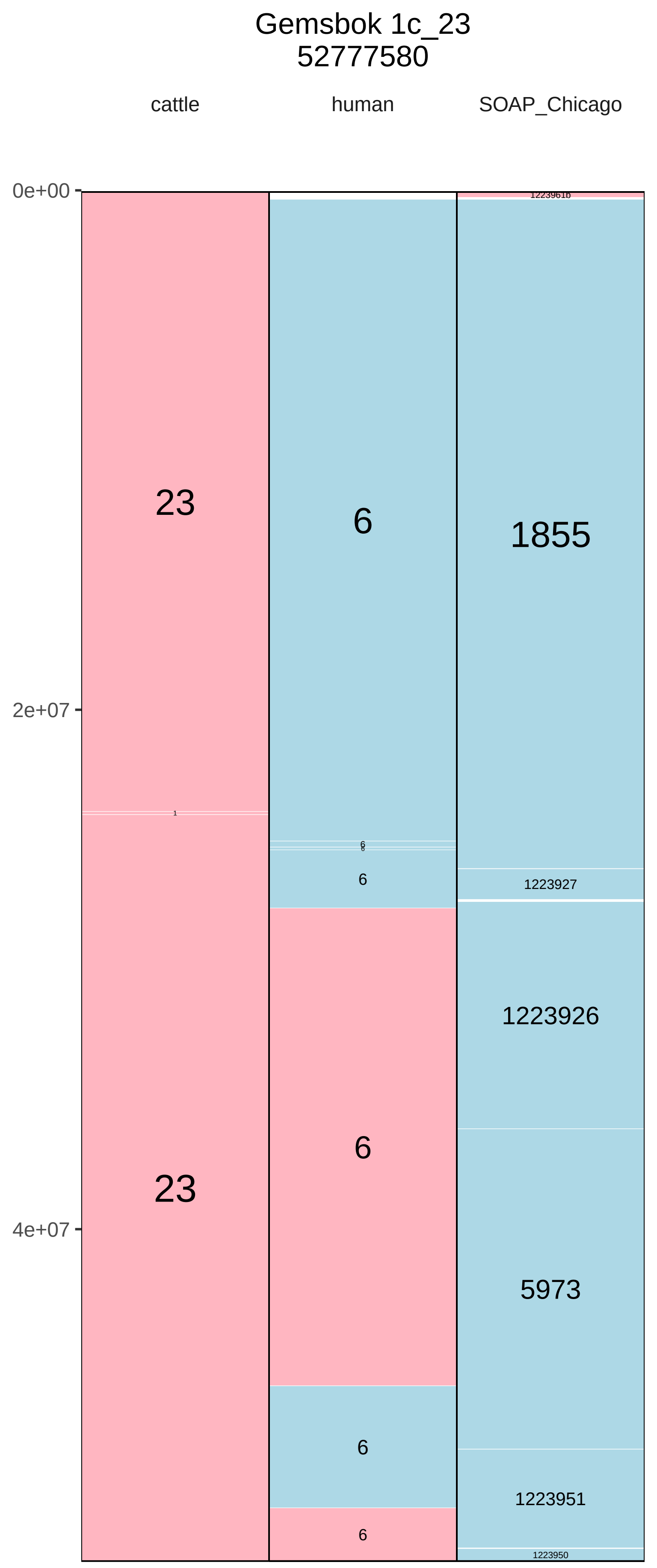

Gemsbok 1c_23
52777580
cattle
human
SOAP_Chicago
0e+00
2e+07
4e+07
23
1
23
6
6
6
6
6
1223961b
1855
1223927
1223926
5973
1223951
1223950

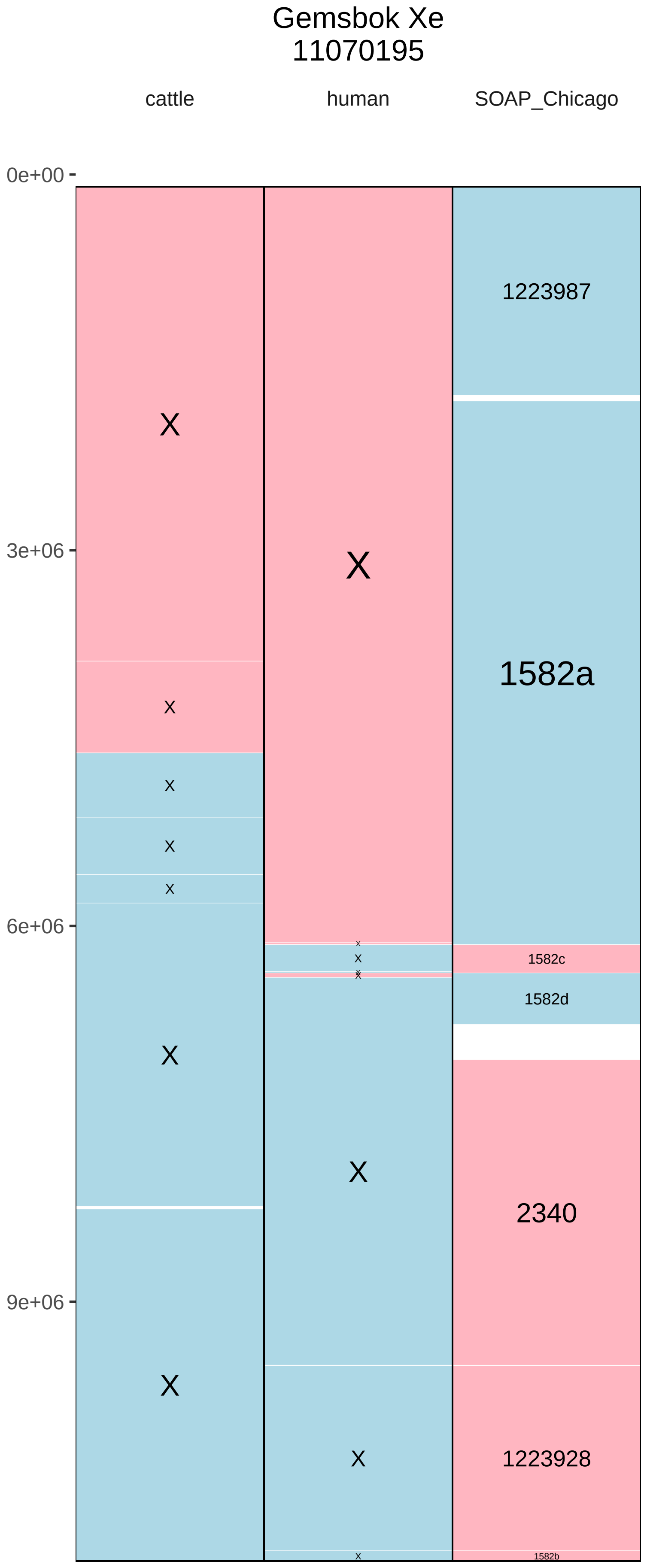

Gemsbok Xe
11070195
cattle
human
SOAP_Chicago
0e+00
3e+06
6e+06
9e+06
X
X
X
X
X
X
X
X
X
X
X
X
1223987
1582a
1582c
1582d
2340
1223928
1582b

Gemsbok 2
142327305

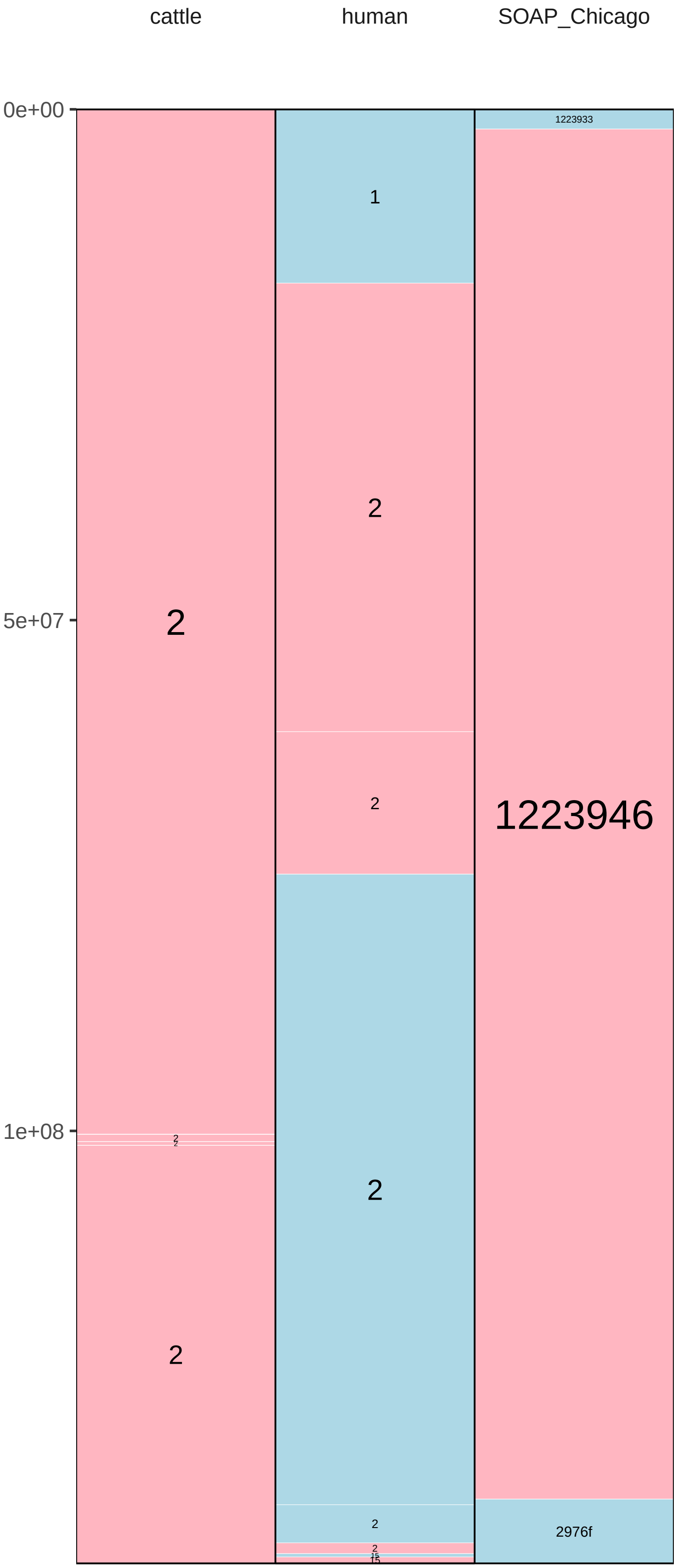

Gemsbok 26
54165646

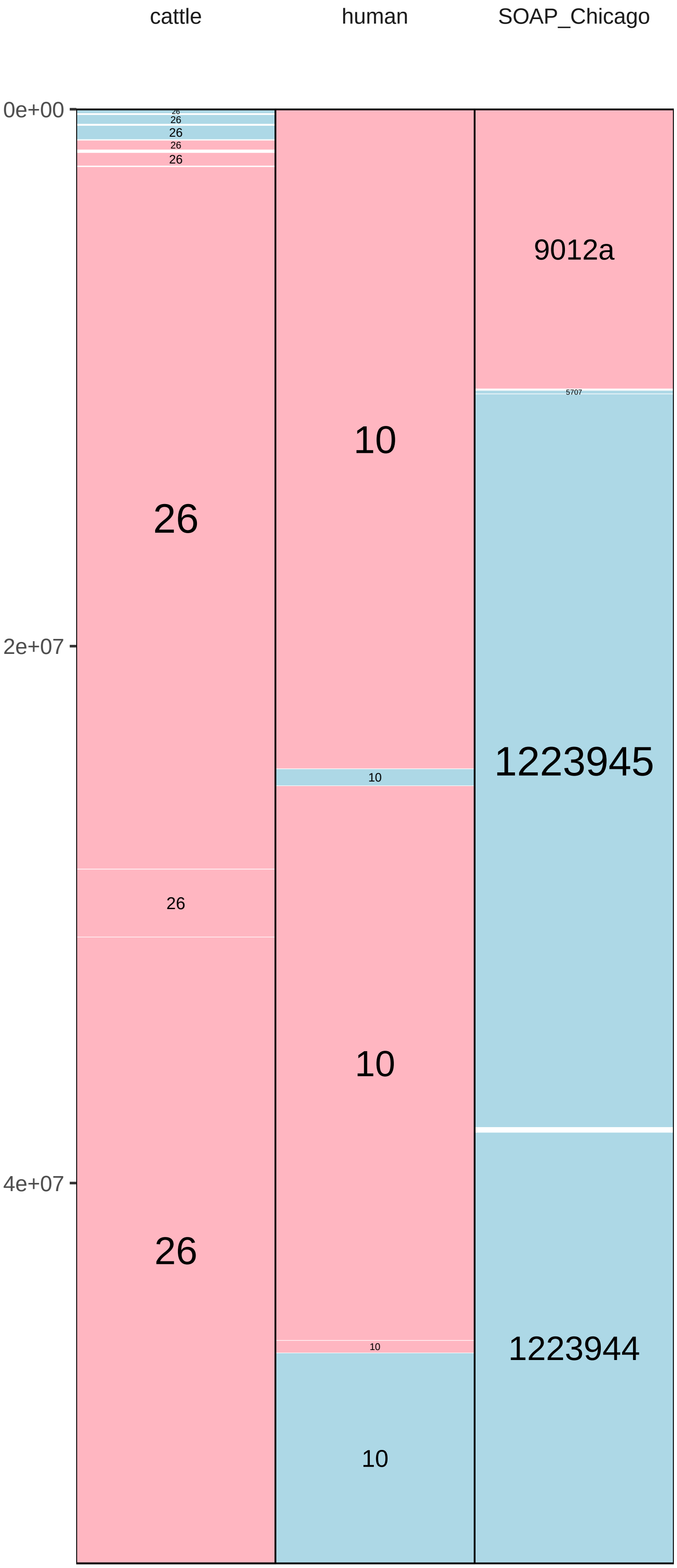

Gemsbok 4a
52260288

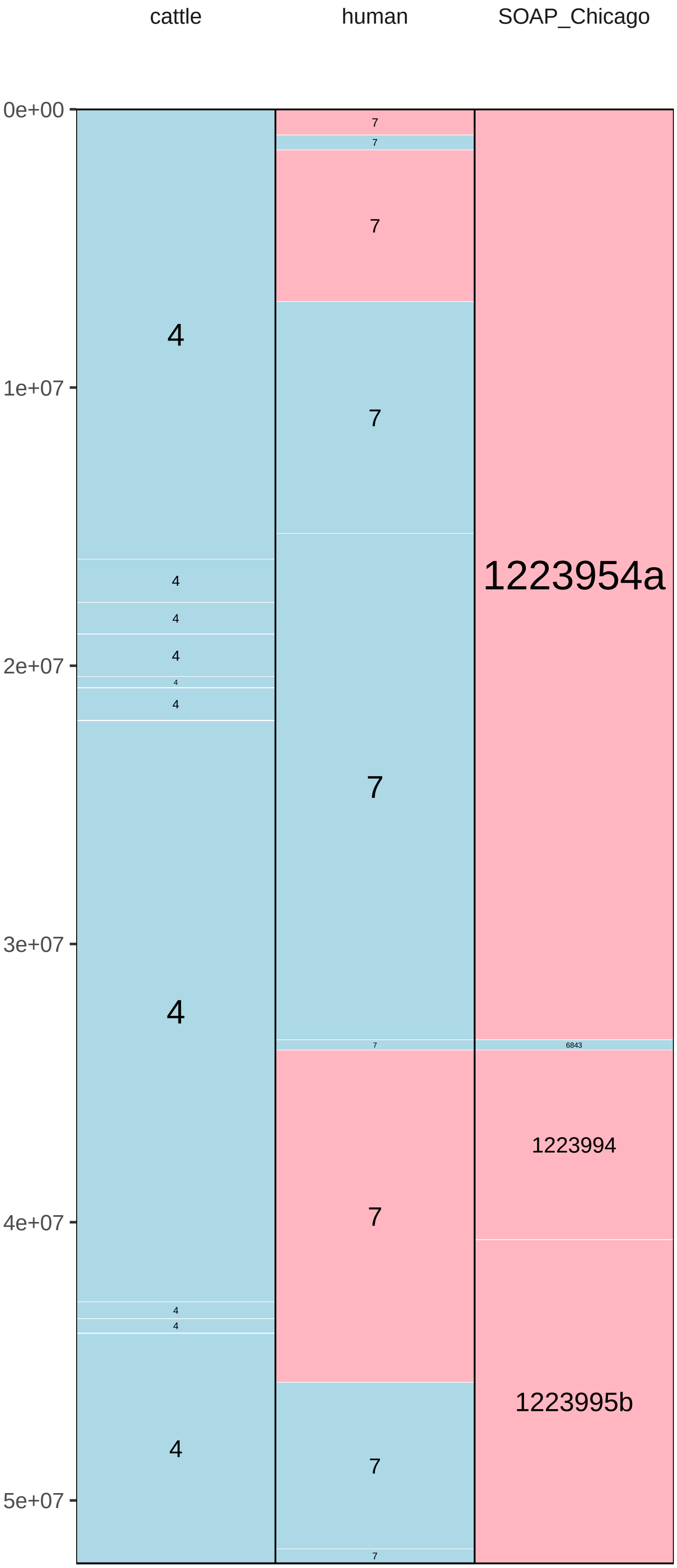

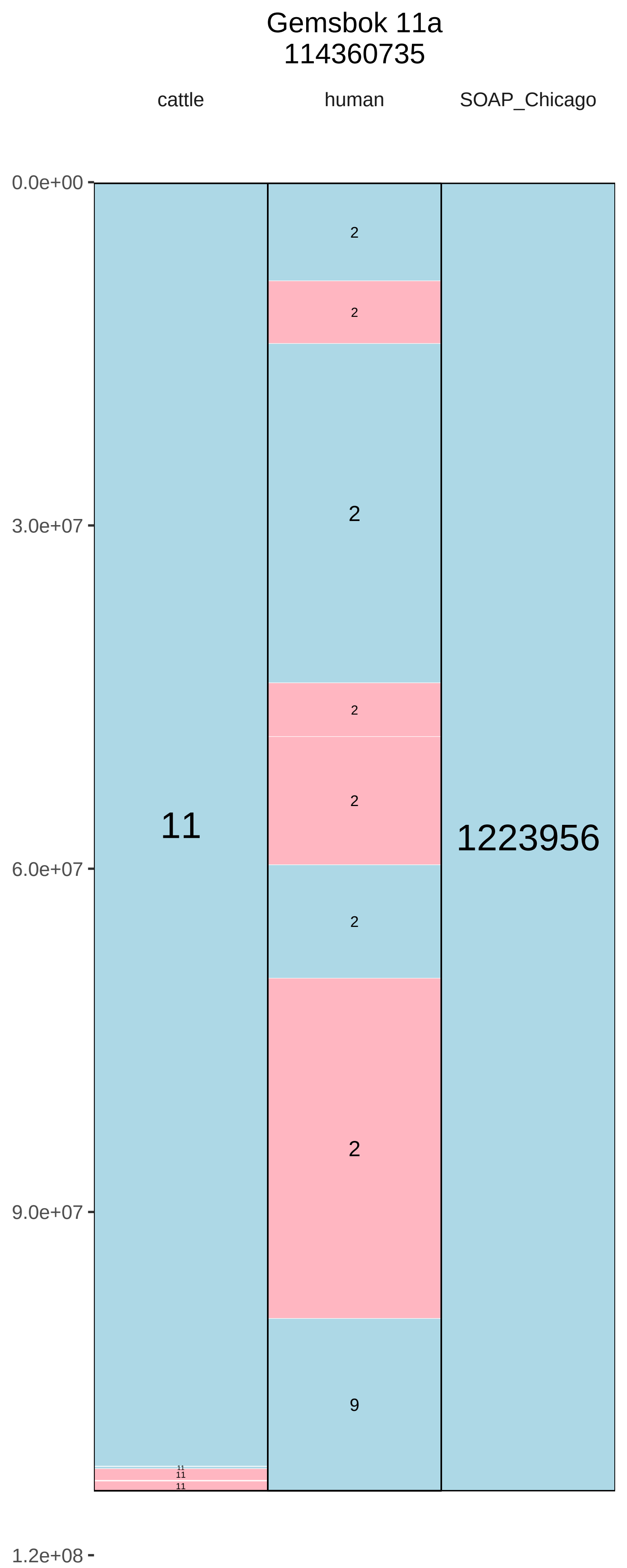

Gemsbok 11a
114360735
cattle
human
SOAP_Chicago
0.0e+00
3.0e+07
6.0e+07
9.0e+07
1.2e+08
11
11
11
11
2
2
2
2
2
2
2
9
1223956

Gemsbok 9
108734999

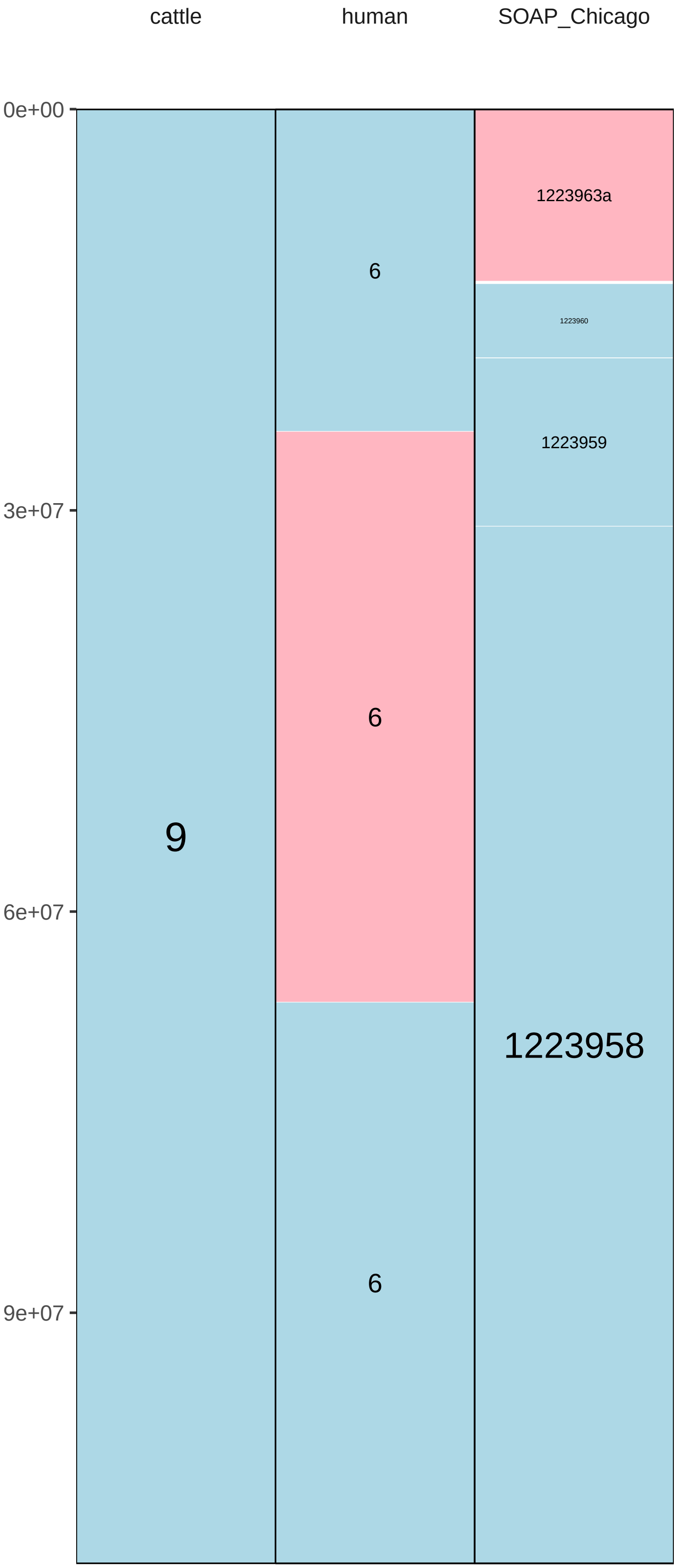

Gemsbok 25
46724120

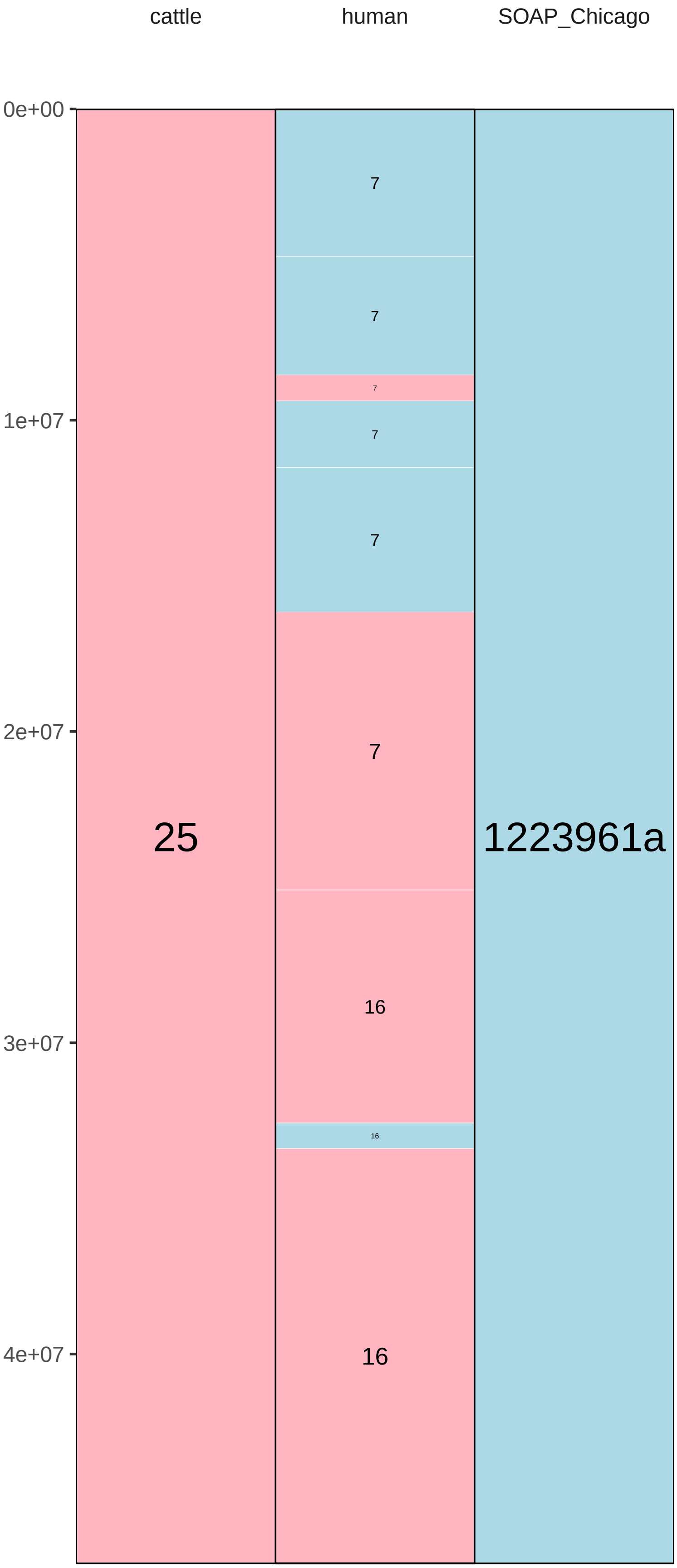

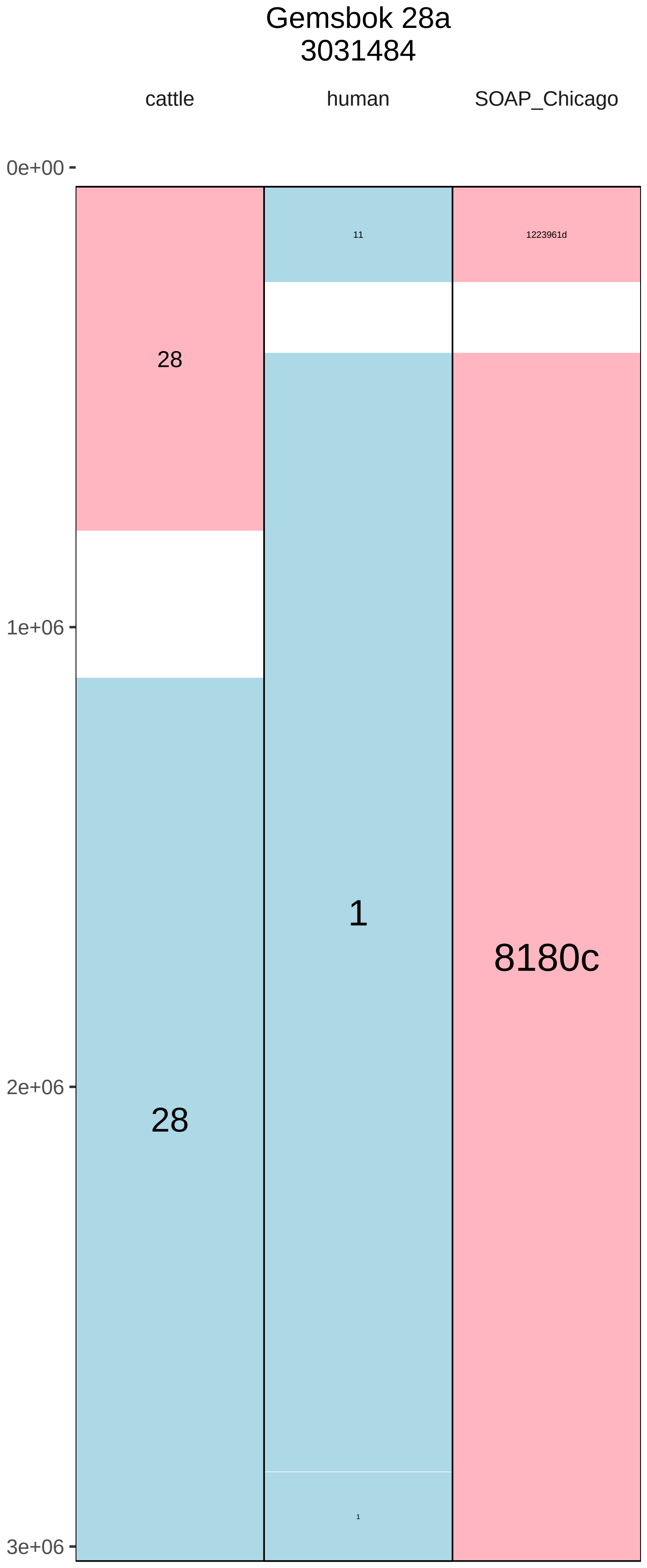

Gemsbok 28a
3031484
cattle
human
SOAP_Chicago
0e+00
1e+06
2e+06
3e+06
28
11
1223961d
1
8180c
28
1

Gemsbok 21a
73412713

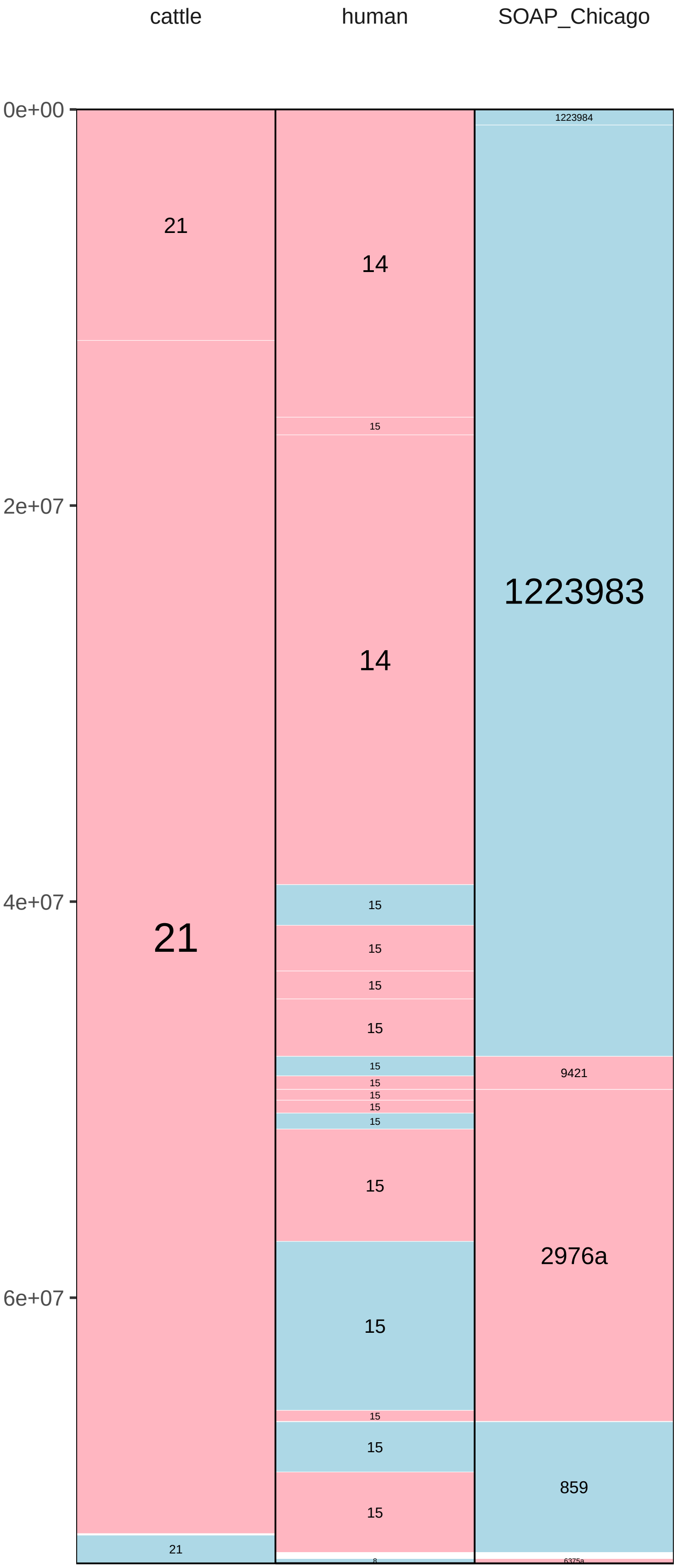

Gemsbok 6
121836686

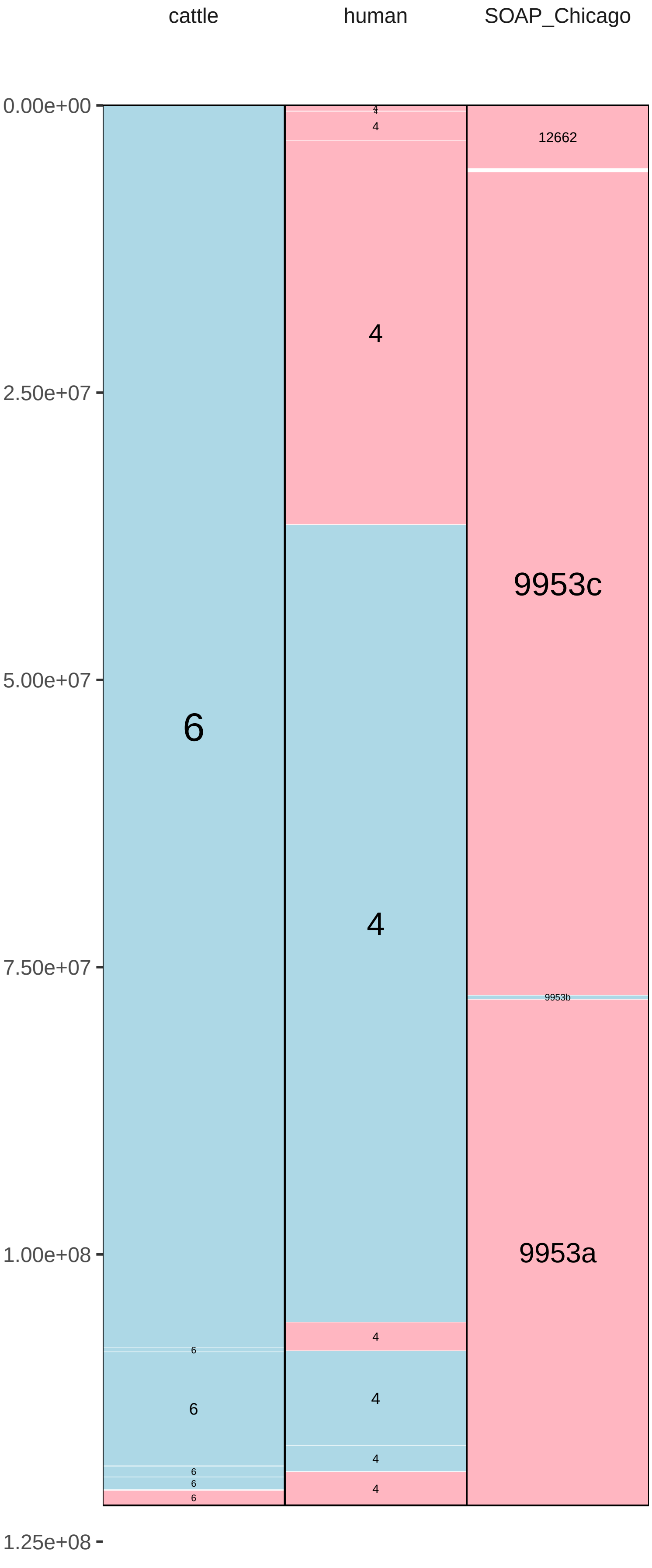

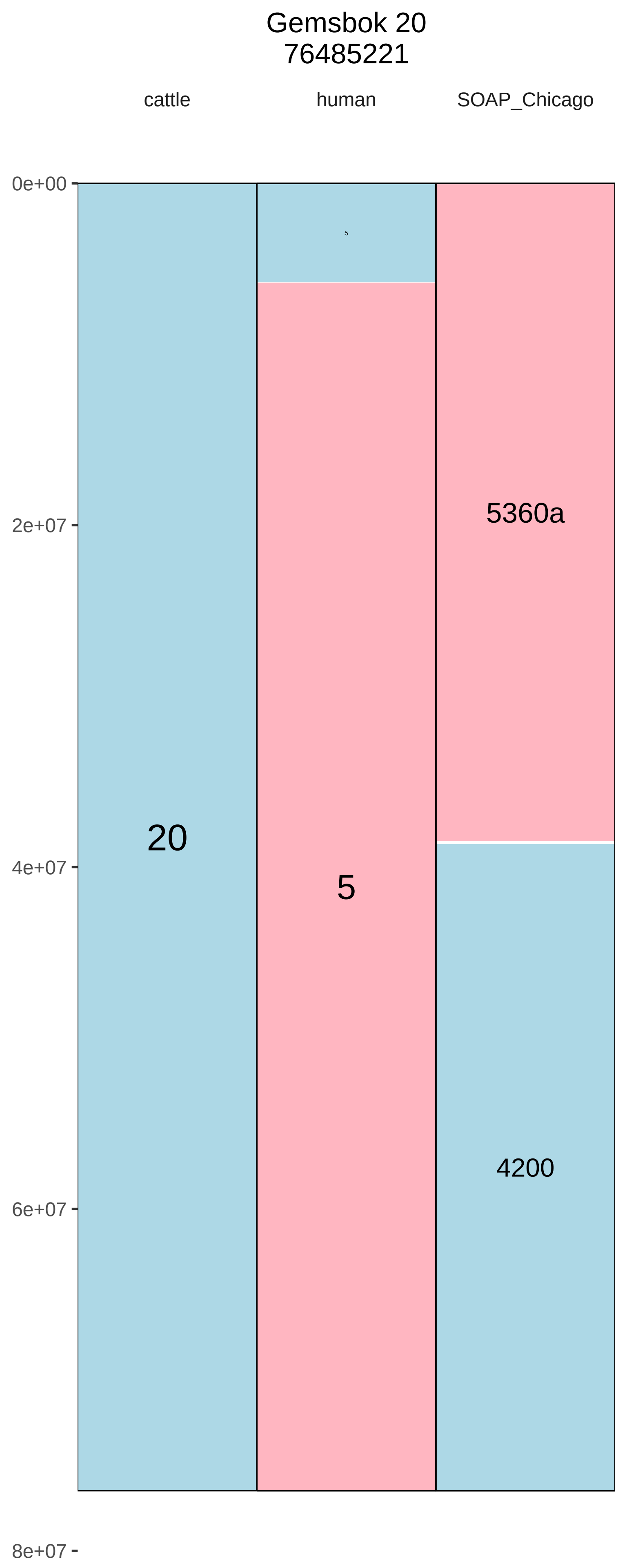
Gemsbok 20
76485221
cattle
human
SOAP_Chicago
0e+00
2e+07
4e+07
6e+07
8e+07
20
5
5
5360a
4200

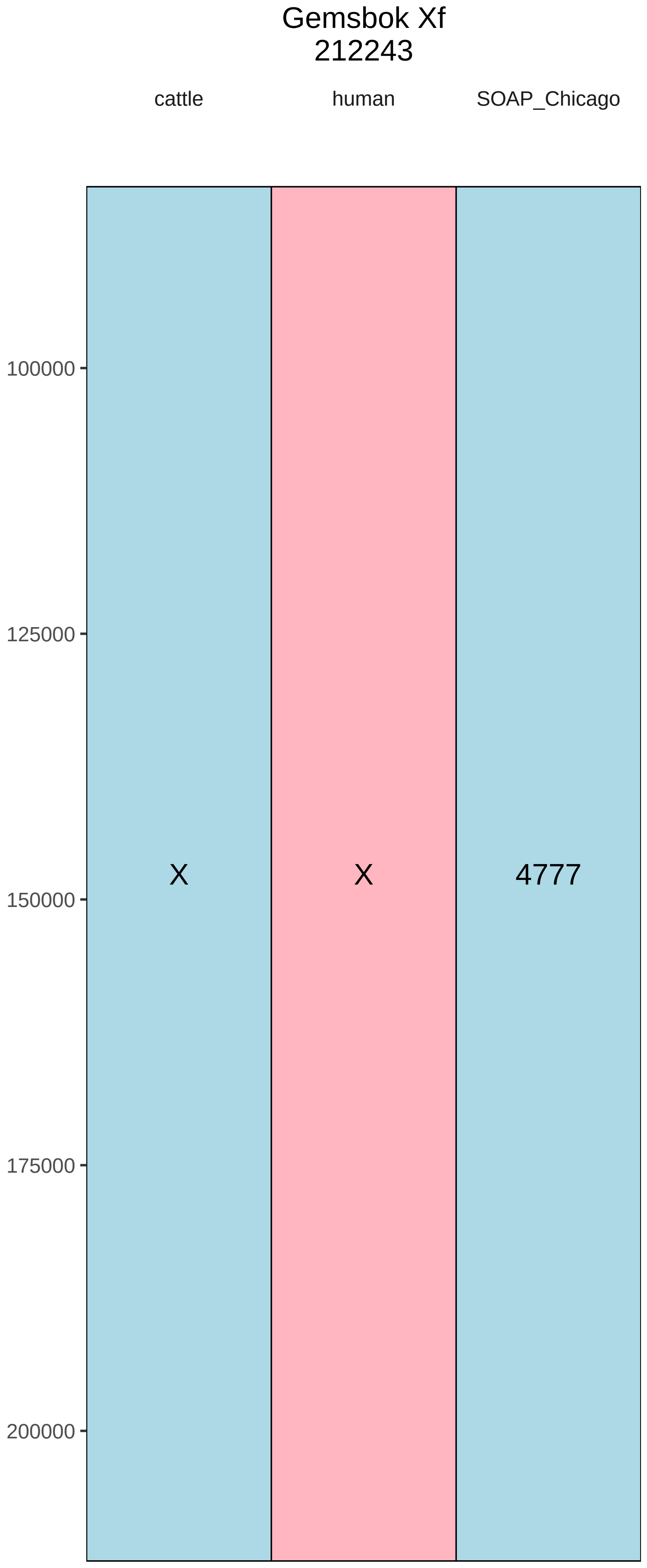
Gemsbok Xf
212243
cattle
human
SOAP_Chicago
100000
125000
150000
175000
200000
X
X
4777

Gemsbok 21b_27
46360443

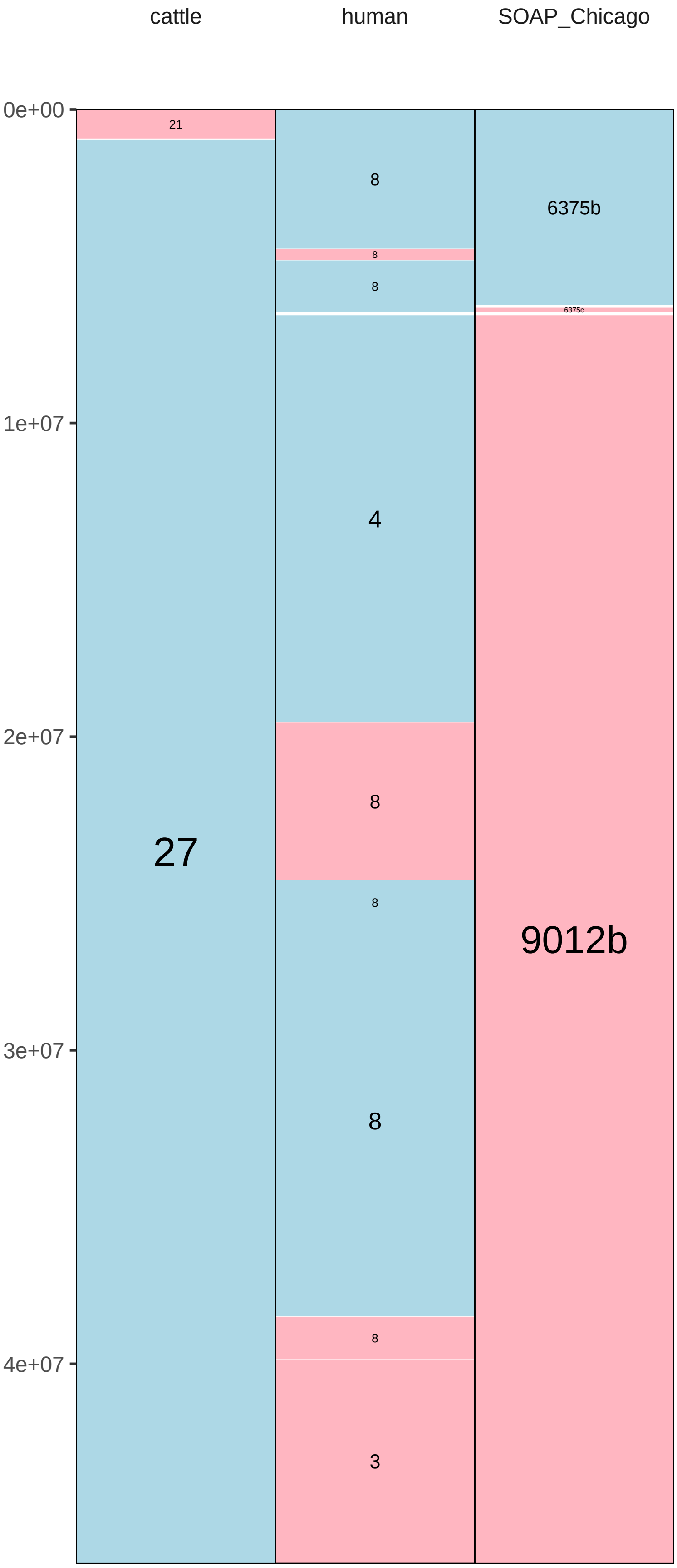

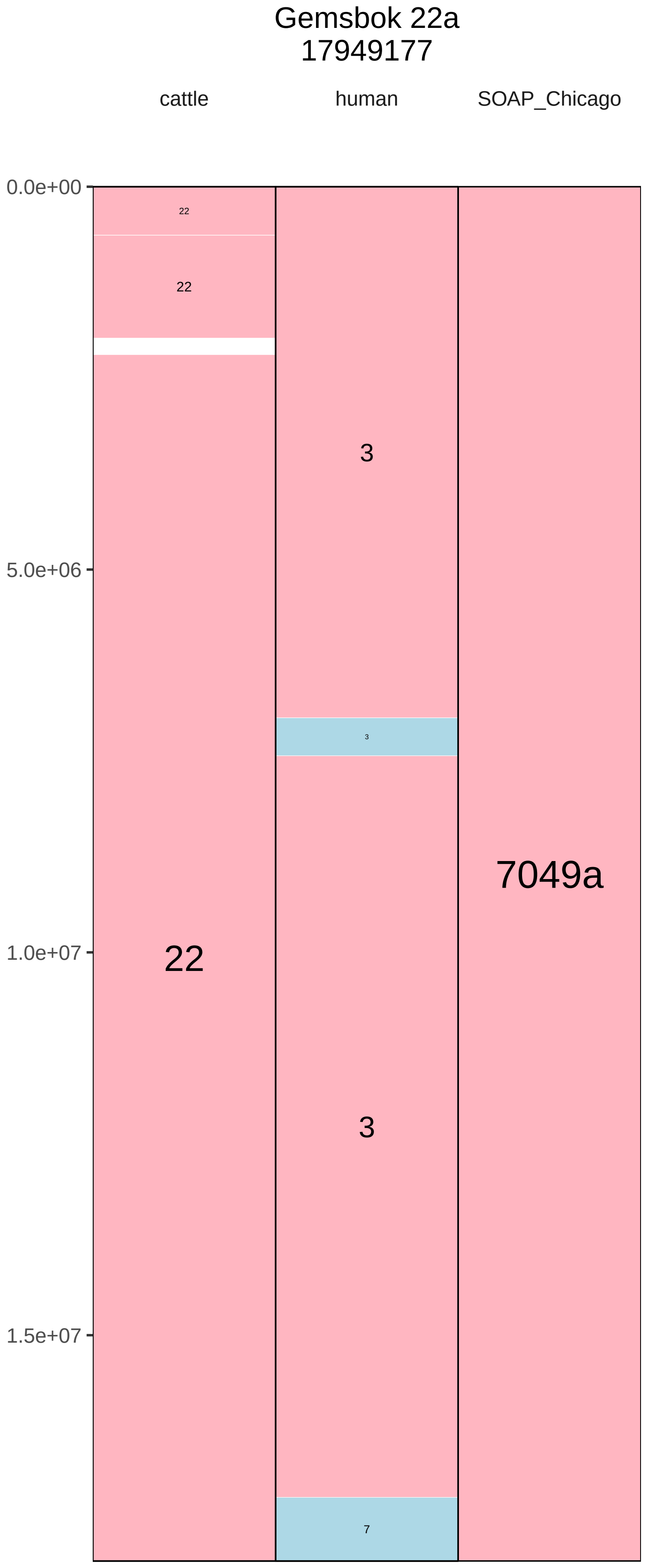
Gemsbok 22a
17949177
cattle
human
SOAP_Chicago
0.0e+00
5.0e+06
1.0e+07
1.5e+07
22
22
22
3
3
3
7
7049a

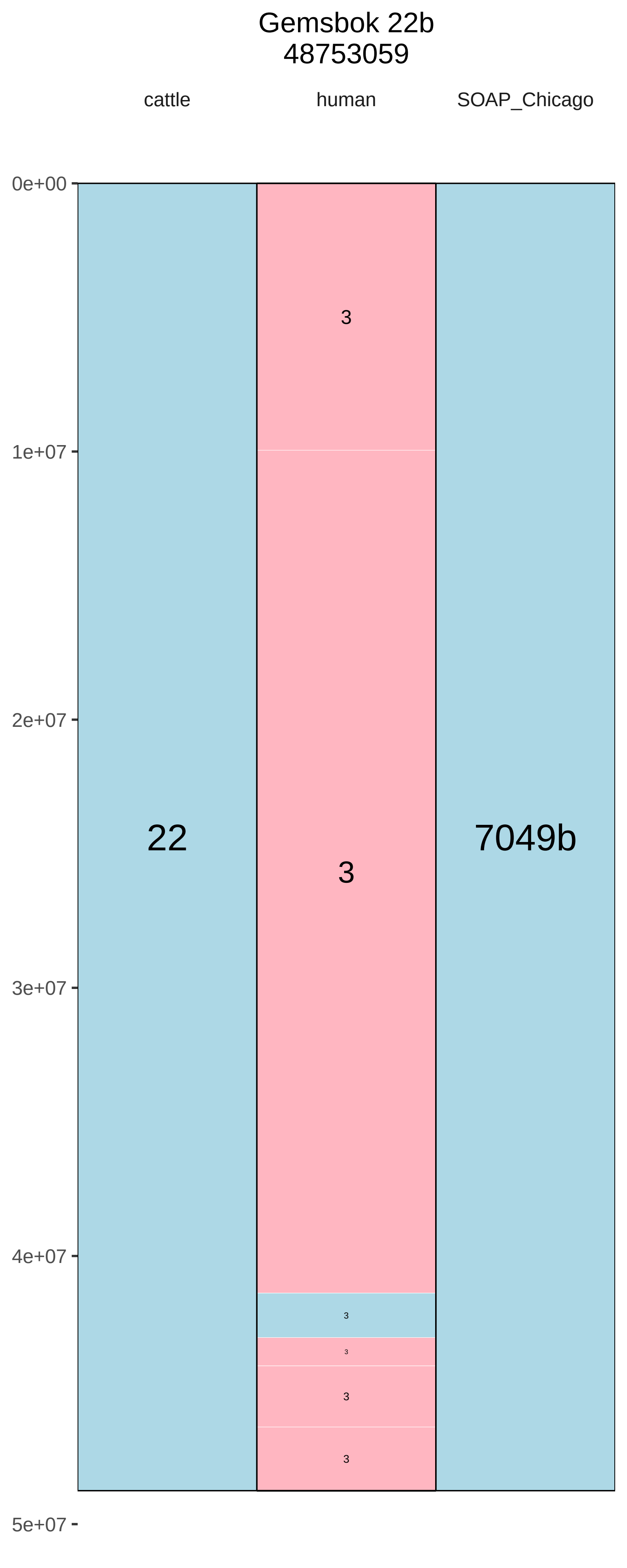
Gemsbok 22b
48753059
cattle
human
SOAP_Chicago
0e+00
1e+07
2e+07
3e+07
4e+07
5e+07
22
3
3
7049b
3
3
3
3

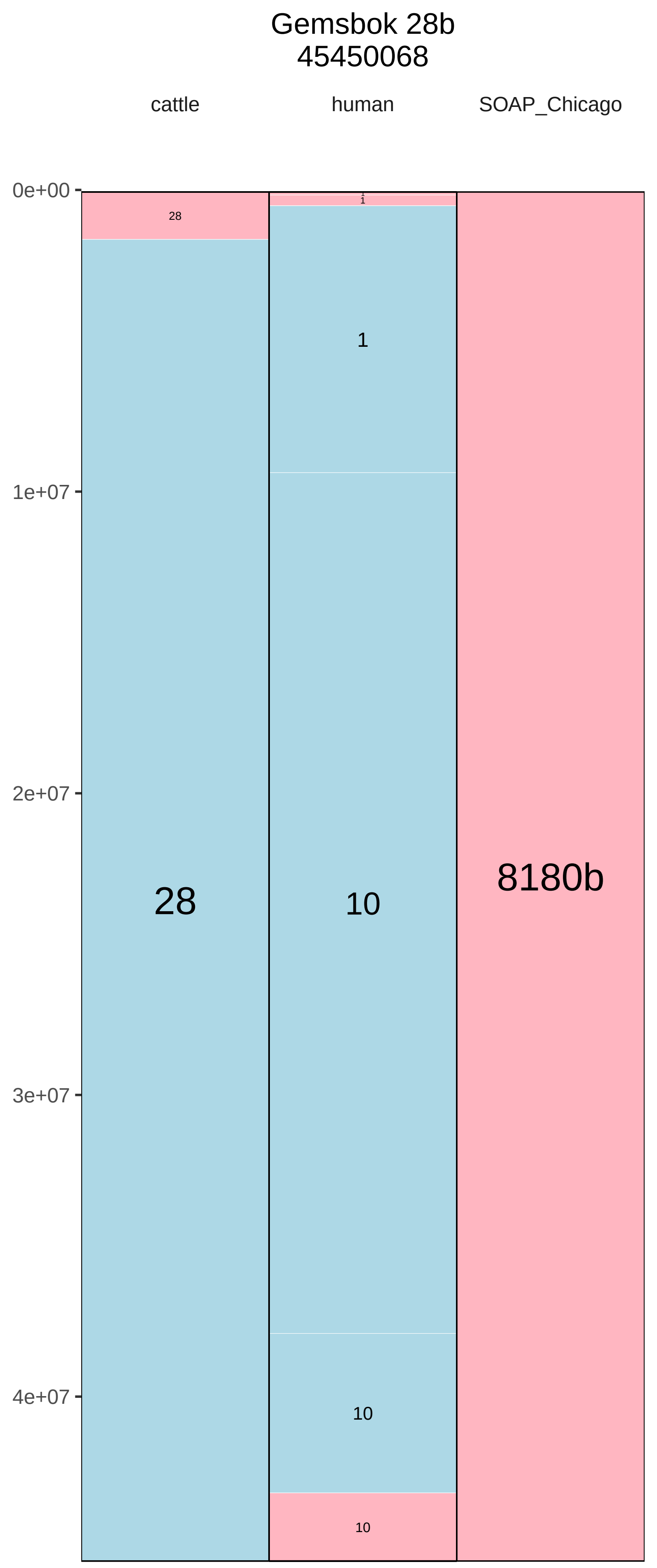

Gemsbok 28b
45450068
cattle
human
SOAP_Chicago
0e+00
1e+07
2e+07
3e+07
4e+07
28
28
1
1
1
10
10
10
8180b

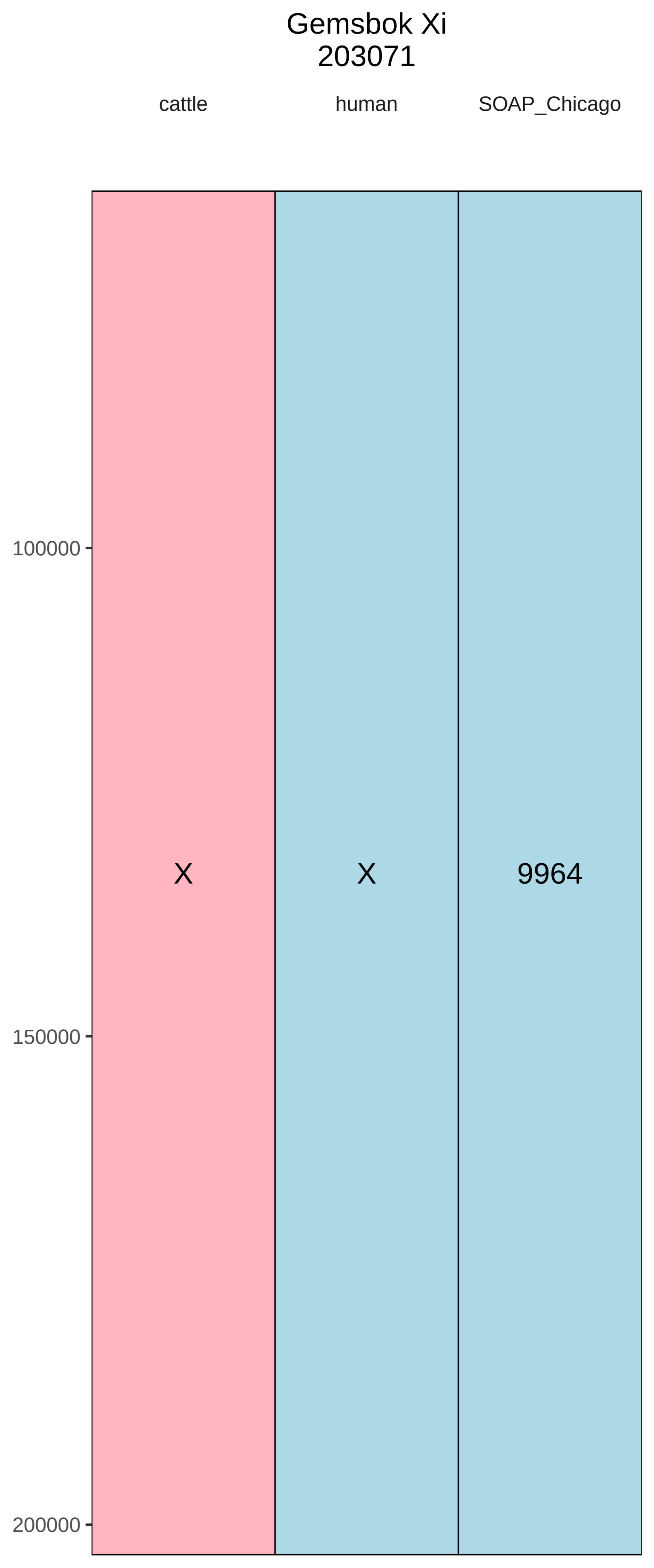
Gemsbok Xi
203071
cattle
human
SOAP_Chicago
100000
150000
200000
X
X
9964

Gemsbok 16b
1214457

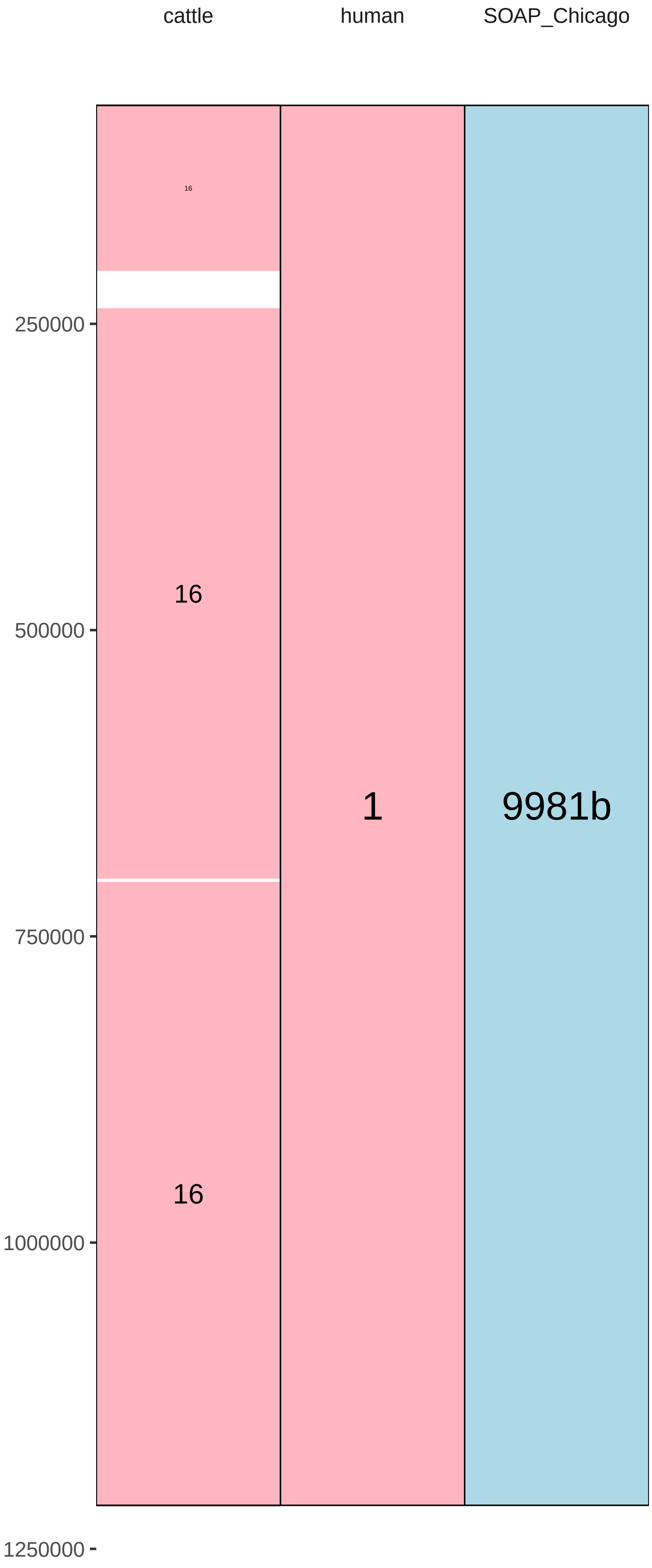

Supplement: Supplemental Files [file giy162_supplemental_files.zip › SupplFigure1.pdf]
