# Supplementary material for: A near-chromosome-scale genome assembly of the gemsbok (Oryx gazella): an iconic antelope of the Kalahari desert
Source: Gigascience. 2019 Jan 16;8(2):giy162. doi: 10.1093/gigascience/giy162 (PMC6351727; doi:10.1093/gigascience/giy162)
Supplement: Response_to_Reviewer_Comments_Original_Submission.pdf [file giy162_response_to_reviewer_comments_original_submission.pdf]

Author's Response To Reviewer Comments

Close

Dear Mr Zauner,

We are very grateful for your time and the time of two referees who reviewed our manuscript. We were very pleased to find that the paper could be accepted for publication in GigaScience just after some minor modifications.

Our detailed responses to reviewers' comments are included below.

We also submit the modified manuscript with all changes highlighted as well as the modified figures, tables and supplementary files.

Sincerely yours,

Marta Farré
Denis Larkin
Harris Lewin

Editor comments: As you will see, both reviewers don't have major issues with the quality of the work, overall, but reviewer 2 doubts whether it is a sufficient advance to merit publication. After some discussion with the editorial team, we feel that it is indeed a borderline case, as usually our data notes present more complete assemblies these days. After some discussion among the editorial team, we sided with the more encouraging advice of reviewer 1. However, we feel that it would be helpful if you can add some additional value to the paper, e.g. by adding the circos plot on chromosome synteny as recommended by reviewer 1.

Reply: We are very grateful that the editorial team finds our manuscript worth of publication in GigaScience. We have included a circos plot showing the chromosome synteny between gemsbok and cattle assemblies as suggested (Figure 4a).

Editor comments: In addition to the reviewers' comments, please note that one feature of most of our genome Data Notes is a phylogeny of some related species, to give the readers some impression of the position of the newly sequenced species. If this is feasible, please consider including this as well.

Reply: We have included a phylogeny showing the relation of our newly sequenced species with other ruminants (cattle, yak and sheep) as well as other mammalian species (horse and human). We added a new section in the manuscript detailing this analysis and its results, and a new figure (Figure 5).

Editor comments: I also note that you mention that visualizations of the different assemblies will be available via the Evolution Highway site - I didn't manage to access this, please make

sure this is accessible when you submit the revised version. I feel it may also be informative to include some of these visualizations (e.g. as screenshots) in the manuscript and briefly discuss them, if it's not redundant with information that is already included in the paper.

Reply: Evolution Highway now contains all the data, but we have also included screenshots of all the chromosomes as Supplementary Figure 1. Moreover, one Evolution Highway chromosome is now part of Figure 4.

Editor comments: Please also re-consider the title - in the light of reviewer 2's comment, I feel it is a bit misleading to label the assembly as "chromosome scale".

Reply: We agree with the reviewer that it might be misleading and have modified the title accordingly. It now reads: “A near-chromosome scale Genome Assembly of the Gemsbok (Oryx gazella): An Iconic Antelope of the Kalahari Desert”.

Reviewer #1: In this work Farré and colleagues present a genomic assembly for the gemsbok, and African ungulate with interesting adaptations. Using a combination of sequencing and bioinformatic methods the authors have created a chromosome level assembly with a high content of BUSCO genes. This assembly will serve as a reference for future studies of the unique adaptations of gemsbok compared to other ungulates. Overall, I think this is a well written manuscript that applies the latest techniques for genome assembly and annotation. My major comment would be that I think a circos plot of chromosome synteny in the gemsbok compared to domestic cattle would greatly add to the manuscript. Especially given the amount of attention given to the Reference-Assisted Chromosome Assembly tool.

Reply: We thank Reviewer 1 for their very encouraging comment, and we completely agree with them. We have created a new circos plot showing the chromosome synteny between the new gemsbok assembly and cattle genome, as shown in Figure 4. Moreover, data from Evolution Highway showing a detailed analysis of synteny between gemsbok, cattle and human are now part of Figure 4 and in Supplementary Figure 1.

As such, we have updated the manuscript. The text now reads:
“Finally, we assessed the genome continuity by identifying homologous synteny blocks (HSBs) between gemsbok and cattle chromosomes (Suppl. Fig. 1). Gemsbok (2n = 56) and cattle (2n = 60) karyotypes differ by two Robertsonian translocations [7], but only one of them is present in the gemsbok assembly (Figure 4). A total of 21 cattle chromosomes aligned to an individual gemsbok fragment, indicating that they represent complete gemsbok chromosomes. Eight cattle chromosomes (BTA1, BTA3, BTA4, BTA11, BTA16, BTA22, BTA28, and BTAX) were syntenic to two or more gemsbok HSBs, suggesting that these HSBs represent chromosomal fragments. The HSBs were physically-assigned to chromosomes based on known syntenic relationships to cattle chromosomes [7].”

Reviewer #1: Along this line, I noted that in the introduction it is stated that gemsbok are

predicted to have 56 chromosomes, but the final assembly only contains 47. Can the authors comment on this discrepancy? Are these "remaining" chromosomes especially small?

Reply: We thank the reviewer for this comment. Indeed, gemsbok have a diploid number (2n) of 56 chromosomes, representing two copies of each of the 28 unique chromosomes that would be expected in the assembly. Our assembly consists of 47 chromosomal fragments, 21 of them representing entire gemsbok chromosomes, and 8 gemsbok chromosomes assembled into two or more fragments, the latter accounting for the difference from the expected 28.

Reviewer #2: The paper presents a genome assembly of the Gemsbok. The methodology is standard, and the genome is of reasonably good quality. I do not have any concerns other than to say the article presents common analyses that many groups, my group included, regularly conduct and do not publish as it is common to mix technologies and assembly strategies. Moreover, there were "problematic" scaffolds which in normal, but as per Data Notes guidelines, I do not consider this to be an exceptional data set rather a now commonplace non-model organism genome. The chromosome level highlighted in the title is presumably based off of some reference genome (never mentioned but required for RACA?), thus the chromosome order is based off a distant relative, compared to something identified with long-reads, where the latter would truly be an "exceptional" data set as per GigaScience guidelines.

Reply: We thank Reviewer 2 for their time in revising our manuscript. We agree that with new technologies it has now become commonplace to sequence and assemble non-model organisms; however, we believe that the implementation of RACA as an evaluation method will be a powerful approach to assist in the assembly and assess the quality of genome assemblies obtained using third generation methodologies. Moreover, having a genome assembled at near-chromosome level will foster research into the unique adaptations that gemsbok has, as well as helping studies of endangered and closely related species, such as the scimitar oryx, for which a high-quality genome is still not available.
As suggested by the reviewer, we have modified the title of the manuscript. It now reads: “A Near-Chromosome Scale Genome Assembly of the Gemsbok (Oryx gazella): An Iconic Antelope of the Kalahari Desert”.

Gemsbok chromosome assignment was done using synteny comparison to cattle chromosomes and following the publication where gemsbok karyotype was established (Gallagher & Womack 1992). This has now been incorporated in the text, and it reads: “The HSBs were physically-assigned to chromosomes based on known syntenic relationships to cattle chromosomes [7].”

Close
