# Supplementary material for: A near-chromosome-scale genome assembly of the gemsbok (Oryx gazella): an iconic antelope of the Kalahari desert
Source: Gigascience. 2019 Jan 16;8(2):giy162. doi: 10.1093/gigascience/giy162 (PMC6351727; doi:10.1093/gigascience/giy162)
Supplement: Reviewer_1_Report_Orginal_Submission -- Joshua Moses Miller — 10/31/2018 Reviewed [file giy162_reviewer_1_report_orginal_submission.pdf]

**Reviewer Report**

**Title: A Near-Chromosome Scale Genome Assembly of the Gemsbok (Oryx gazella): An Iconic Antelope of the Kalahari Desert**

**Version: Original Submission** **Date:** 10/31/2018

**Reviewer name: Joshua Moses Miller**

**Reviewer Comments to Author:**

In this work FarrÃ© and colleagues present a genomic assembly for the gemsbok, and African ungulate with interesting adaptations. Using a combination of sequencing and bioinformatic methods the authors have created a chromosome level assembly with a high content of BUSCO genes. This assembly will serve as a reference for future studies of the unique adaptations of gemsbok compared to other ungulates. Overall, I think this is a well written manuscript that applies the latest techniques for genome assembly and annotation. My major comment would be that I think a circos plot of chromosome synteny in the gemsbok compared to domestic cattle would greatly add to the manuscript. Especially given the amount of attention given to the Reference-Assisted Chromosome Assembly tool.

Along this line, I noted that in the introduction it is stated that gemsbok are predicted to have 56 chromosomes, but the final assembly only contains 47. Can the authors comment on this discrepancy? Are these "remaining" chromosomes especially small?

**Level of Interest**

Please indicate how interesting you found the manuscript: Choose an item.

**Quality of Written English**

Please indicate the quality of language in the manuscript: Choose an item.

**Declaration of Competing Interests**

Please complete a declaration of competing interests, considering the following questions:

- Have you in the past five years received reimbursements, fees, funding, or salary from an organisation that may in any way gain or lose financially from the publication of this manuscript, either now or in the future?
- Do you hold any stocks or shares in an organisation that may in any way gain or lose financially from the publication of this manuscript, either now or in the future?
- Do you hold or are you currently applying for any patents relating to the content of the manuscript?

- Have you received reimbursements, fees, funding, or salary from an organization that holds or has applied for patents relating to the content of the manuscript?
- Do you have any other financial competing interests?
- Do you have any non-financial competing interests in relation to this paper?

If you can answer no to all of the above, write 'I declare that I have no competing interests' below. If your reply is yes to any, please give details below.

I declare that I have no competing interests

I agree to the open peer review policy of the journal. I understand that my name will be included on my report to the authors and, if the manuscript is accepted for publication, my named report including any attachments I upload will be posted on the website along with the authors' responses. I agree for my report to be made available under an Open Access Creative Commons CC-BY license (http://creativecommons.org/licenses/by/4.0/). I understand that any comments which I do not wish to be included in my named report can be included as confidential comments to the editors, which will not be published.

I agree to the open peer review policy of the journal
