# Supplementary material for: A near-chromosome-scale genome assembly of the gemsbok (Oryx gazella): an iconic antelope of the Kalahari desert
Source: Gigascience. 2019 Jan 16;8(2):giy162. doi: 10.1093/gigascience/giy162 (PMC6351727; doi:10.1093/gigascience/giy162)
Supplement: Reviewer_2_Report_Orginal_Submission -- Aaron Shafer — 11/5/2018 Reviewed [file giy162_reviewer_2_report_orginal_submission.pdf]

Reviewer Report

Title: A Near-Chromosome Scale Genome Assembly of the Gemsbok (Oryx gazella): An Iconic Antelope of the Kalahari Desert

Version: Original Submission **Date:** 11/5/2018

Reviewer name: Aaron Shafer

Reviewer Comments to Author:

The paper presents a genome assembly of the Gemsbok. The methodology is standard and the genome is of reasonably good quality. I do not have any concerns other than to say the article presents common analyses that many groups, my group included, regularly conduct and do not publish as it is common to mix technologies and assembly strategies. Moroever, there were "problematic" scaffolds which in normal, but as per Data Notes guidelines, I do not considerr this to be an exceptional data set rather a now commonplace non-model organism genome. The chromosome level highlighted in the title is presumably based off of some reference genome (never mentioned but required for RACA?), thus the chromosome order is based off a distant relative, compared to something identified with long-reads, where the latter would truly be an "exceptional" data set as per GigaScience guidelines.

Level of Interest

Please indicate how interesting you found the manuscript: Choose an item.

Quality of Written English

Please indicate the quality of language in the manuscript: Choose an item.

Declaration of Competing Interests

Please complete a declaration of competing interests, considering the following questions:

- Have you in the past five years received reimbursements, fees, funding, or salary from an organisation that may in any way gain or lose financially from the publication of this manuscript, either now or in the future?
- Do you hold any stocks or shares in an organisation that may in any way gain or lose financially from the publication of this manuscript, either now or in the future?
- Do you hold or are you currently applying for any patents relating to the content of the manuscript?
- Have you received reimbursements, fees, funding, or salary from an organization that holds or has applied for patents relating to the content of the manuscript?
- Do you have any other financial competing interests?
- Do you have any non-financial competing interests in relation to this paper?

If you can answer no to all of the above, write 'I declare that I have no competing interests' below. If your reply is yes to any, please give details below.

I declare that I have no competing interests

I agree to the open peer review policy of the journal. I understand that my name will be included on my report to the authors and, if the manuscript is accepted for publication, my named report including any attachments I upload will be posted on the website along with the authors' responses. I agree for my report to be made available under an Open Access Creative Commons CC-BY license (http://creativecommons.org/licenses/by/4.0/). I understand that any comments which I do not wish to be included in my named report can be included as confidential comments to the editors, which will not be published.

I agree to the open peer review policy of the journal
